# Mass spectrometry analysis of adipose-derived stem cells reveals a significant effect of hypoxia on pathways regulating extracellular matrix

**DOI:** 10.1186/s13287-016-0310-7

**Published:** 2016-04-14

**Authors:** Simone Riis, Allan Stensballe, Jeppe Emmersen, Cristian Pablo Pennisi, Svend Birkelund, Vladimir Zachar, Trine Fink

**Affiliations:** Department of Health Science and Technology, Laboratory for Stem Cell Research, Aalborg University, Fredrik Bajers Vej 3B, Aalborg, 9220 Denmark; Department of Health Science and Technology, Laboratory for Medical Mass Spectrometry, Aalborg University, Aalborg, Denmark

**Keywords:** ASCs, Proteomics, Hypoxia, Serum-free culture, Secretome, ECM, Mass spectrometry

## Abstract

**Background:**

Adipose-derived stem cells (ASCs) are being increasingly recognized for their potential to promote tissue regeneration and wound healing. These effects appear to be partly mediated by paracrine signaling pathways, and are enhanced during hypoxia. Mass spectrometry (MS) is a valuable tool for proteomic profiling of cultured ASCs, which may help to reveal the identity of the factors secreted by the cells under different conditions. However, serum starvation which is essentially required to obtain samples compatible with secretome analysis by MS can have a significant influence on ASCs. Here, we present a novel and optimized culturing approach based on the use of a clinically relevant serum-free formulation, which was used to assess the effects of hypoxia on the ASC proteomic profile.

**Methods:**

Human ASCs from three human donors were expanded in StemPro® MSC SFM XenoFree medium. Cells were cultured for 24 h in serum- and albumin-free supplements in either normoxic (20 %) or hypoxic (1 %) atmospheres, after which the cells and conditioned medium were collected, subfractionated, and analyzed using MS. Prior to analysis, the secreted proteins were further subdivided into a secretome (>30 kDa) and a peptidome (3–30 kDa) fraction.

**Results:**

MS analysis revealed the presence of 342, 98, and 3228 proteins in the normoxic ASC secretome, peptidome, and proteome, respectively. A relatively small fraction of the proteome (9.6 %) was significantly affected by hypoxia, and the most regulated proteins were those involved in extracellular matrix (ECM) synthesis and cell metabolism. No proteins were found to be significantly modulated by hypoxic treatment across all cultures for the secretome and peptidome samples.

**Conclusions:**

This study highlights ECM remodeling as a significant mechanism contributing to the ASC regenerative effect after hypoxic preconditioning, and further underscores considerable inter-individual differences in ASC response to hypoxia. The novel culture paradigm provides a basis for future proteomic studies under conditions that do not induce a stress response, so that the best responders can be accurately identified for prospective therapeutic use.

Data are available via ProteomeXchange with identifier PXD003550.

**Electronic supplementary material:**

The online version of this article (doi:10.1186/s13287-016-0310-7) contains supplementary material, which is available to authorized users.

## Background

Human adipose-derived stem cells (ASCs) have come under scrutiny for their putative use in regenerative medicine based on their immunomodulatory, pro-angiogenic, pro-trophic, and anti-apoptotic properties [1–4]. ASCs are of particular interest for the treatment of chronic wounds as they may restart the healing process by reducing inflammation, supporting ingrowth of new vessels into the hypoxic tissue, and promoting fibroblast and keratinocyte proliferation and migration [5]. While the molecular mechanisms are not fully explained, it is apparent that some of these regenerative properties of ASCs are associated with secreted factors in the form of growth factors, cytokines, and extracellular vesicles [6–9]. In addition to the impact on the inflammatory and proliferative phases of wound healing, it also appears that ASCs have a positive effect on the formation and remodeling of the extracellular matrix (ECM) [10]. This is particularly important as the ECM is degraded or otherwise compromised during the chronic wound process [11].

It has been demonstrated that the regenerative potential of ASCs and other mesenchymal stem cells is enhanced by hypoxic conditioning prior to use [12, 13]. Of interest to wound healing, hypoxic treatment induces an increase in ASC secretion of factors relevant to the inflammation and proliferation phases, such as interleukin (IL)-10, basic fibroblast growth factor (bFGF), vascular endothelial growth factor (VEGF), stromal cell-derived factor 1 (SDF-1), insulin-like growth factor-1 (IGF-1), caspase 9 (CASP9), and bcl-2 associated X protein (BAX) [2, 14–18]; 1 % O_2_ has especially been shown to increase the secretion of paracrine factors [16]. The identified factors, however, represent only a subset of the entire secretome, and it is probable that as yet unidentified factors play an important role in the wound healing mediated by hypoxia-preconditioned ASCs.

To perform a more global characterization of the effect of hypoxia on ASCs, transcriptional profiling using microarrays has been used to assess the effect on the transcription of a large number of pre-selected genes [19]. High-throughput RNA sequencing that allows for detection of unknown transcripts, and that has been shown suitable for discrimination between distinct ASC subpopulations and between cell populations within the stem cell niche [20, 21], would be another option; however, we have not identified any such study. Both microarrays and RNA sequencing have developed into cost-effective and robust techniques. A serious drawback, however, is the concern regarding a lack of correlation between regulation of transcripts and proteins, particularly when it comes to upregulated genes following perturbation of steady-state conditions [22, 23]. As it is increasingly clear that mRNA abundance does not necessarily reflect the corresponding protein abundance, post-translational modifications, or subcellular location, much effort has been dedicated to characterize and quantitate the ASC secretome in particular [24], and also to some extent the proteome [25]. Techniques used to characterize the effect of hypoxia on the ASC protein profile range from enzyme-linked immunosorbent assay (ELISA), Western blotting, and antibody-based arrays (allowing for the simultaneous analysis of relatively few pre-selected proteins), to mass spectrometry (MS)-based methods where a much larger gamut of proteins can be identified and quantitated (see [24], and references therein). Generally, the large-scale study of proteins in cells or tissue can be made by two proteomics strategies, either using one- or two-dimentional gel electrophoresis or non-gel-based techniques. In non-gel-based techniques, sample proteins are enzymatically digested in solution phase and separated using liquid chromatography in line with a tandem MS (LC-MS/MS) system [26]. During MS and tandem MS analysis the exact mass and amino acid sequence information for each peptide are obtained. The proteins in the sample can then be identified using database search algorithms and quantified by comparing the peptides to databases containing protein information based on translated genome and mRNA sequences [27].

In previous studies, where the proteome and secretome of ASCs have been analyzed by MS, cells have been expanded in medium supplemented with fetal calf serum (FCS), followed by extensive washes and culture in serum-free conditions for 24–72 h prior to collection of conditioned media/cell lysis [28–30]. This period of serum starvation has been necessary as the presence of high levels of serum proteins, e.g., albumin, found in serum would mask the presence of low-abundant proteins [31]. However, the process of serum starvation in itself frequently leads to unwanted cell responses, including membrane blebbing and growth arrest [32–34], and has been shown to induce a robust response in ASCs, altering the transcription of more than 100 genes [35]. Several serum-free medium formulations that support the clinically compliant expansion of ASCs are commercially available [36]. In particular, studies have shown that ASCs cultured using StemPro® MSC SFM XenoFree (Thermo Fisher) maintain the essential stem cell properties such as immunophenotype and multilineage differentiation potential [37, 38]. However, the applicability of serum-free medium formulations for the culture of cells used for MS protein analysis of ASCs has not previously been reported.

In this paper, we describe a culture protocol that allows the growth of ASCs under clinically relevant conditions for the production of conditioned media compatible with MS. Growth and viability of ASCs have been assessed in media without FCS and excessive bovine protein contamination. Using the optimal medium formulation for culture and preconditioning of the ASCs we used MS-based proteome analysis to compare the secretome and proteome of cells from three different donors cultured under hypoxic (1 % oxygen) and normoxic (20 % oxygen) conditions.

## Methods

### ASCs

ASCs from three donors were isolated as previously described after written informed consent from the donors and approval by the regional committee on biomedical research ethics of Northern Jutland, Denmark (project no. VN 2005/54) [39–41]. The three different cultures (ASC12, ASC21, and ASC23) have previously been thoroughly characterized by our laboratory both under normoxic and hypoxic conditions, and possess characteristics commensurate with the definition of mesenchymal stem cells [38, 42–47].

### Media

Three different media were used: StemPro–, composed of StemPro® MSC SFM XenoFree (Gibco™, Thermo Fisher, www.thermofisher.com) supplemented with 2 mM l-glutamine (Gibco™), and 100 U/mL penicillin and 0.1 mg/mL streptomycin (Gibco™); StemPro+, (StemPro– supplemented with StemPro supplement); and StemPro E8 (StemPro– supplemented with Essential 8™ Medium supplement (Gibco™)).

### Cell culture

The cells were cultured in polystyrene tissue flasks (CELLSTAR®, Greiner Bio-One, www.gbo.com) coated with CellStart™ CTS™ (Gibco™), in a standard incubator in an atmosphere of 37 °C, 20 % O_2_ and 5 % CO_2_. When replating, TrypLE Select (Gibco™) was used for cell detachment. Unless stated otherwise, ASCs were cultured in StemPro+. For all experiments the ASCs were in passage 6–8.

### Expansion of ASCs in different media and cell counting

To assess the effect of different media on ASC growth, the cells were seeded at a density of 1500 cells/cm^2^ in 48-well plates (CELLSTAR®) in StemPro+, StemPro–, and StemPro E8, and cultured for up to 72 h. At 24, 48, and 72 h the cell number was determined by measuring the amount of DNA using a Quant-iT™ PicoGreen® dsDNA Assay Kit (Invitrogen™). The fluorescence was measured using a Wallac 1420 Victor Multilabel Counter with excitation and emission at 485 nm and 535, respectively; 6.6 pg DNA/cell was used to calculate the cell number. The media were tested in duplicate on each of the three ASC cultures.

### Short-term effect of different media on ASCs

ASCs were seeded in 96-well culture plates at a density 8000 cells/cm^2^. When the culture was 70 % confluent, cells were washed repeatedly with phosphate-buffered saline (PBS). The cells were then incubated for 24 h with either StemPro+, StemPro–, or StemPro E8 media, after which cell morphology and viability were assessed.

For the assessment of cell morphology, phase contrast images were acquired using an Olympus CKX41 microscope (Olympus Life Science) with a PixeLINK PL-A782 camera.

The proportion of viable cells was determined essentially as previously described [48]. In brief, ASCs were stained with 1 μM Yo-PRO-1 (Molecular Probes™), 10 μg/mL PI (Molecular Probes™), and 5 μg/mL Hoechst 33342 (Molecular Probes™), after which images were acquired using an AxioVision software package with a Zeiss Axio Observer.Z1 microscope equipped with an AxioCam MRm camera (Carl Zeiss, Germany). Live, necrotic, and apoptotic cells were counted using Image J 1.47v (NIH, USA), and the proportion of viable cells was calculated as the fraction of Yo-PRO-1 and PI-positive cells relative to the total cell number subtracted from 1. The effect of the media was tested in duplicate on each of the three ASC cultures.

### Statistical analysis of cell growth and viability data

The statistical analysis was performed using SigmaPlot 12.0 (Systat Software, Erkrath, Germany). The normal distribution of each group was assessed by means of the Shapiro-Wilk test. Additionally, variance was tested using an Equal Variance Test. Data are represented as mean ± standard error of the mean (SEM). A *p* value of <0.05 was considered statistically significant. For comparison of more than two groups, a one-way analysis of variance (ANOVA) with Bonferroni’s post hoc test was used.

### Production and fractionation of conditioned media and cell lysate

**Fig. 1:**
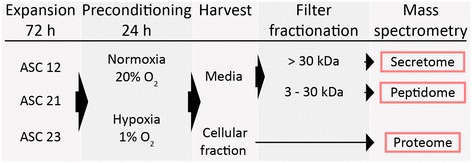
Preparation of samples for mass spectrometric analysis. Following the expansion of ASCs from three donors for 72 h, cells were cultured under either normoxic or hypoxic conditions for 24 h. The conditioned media were harvested and sequentially fractionated through 30-kDa and 3-kDa spin filters to retain the secretome and peptidome fractions, respectively. The cellular fraction was employed for the analysis of the proteome. *ASC* adipose-derived stem cell

For an overview of the steps involved in the production of media and cell lysate for MS, please refer to Fig. 1. For production of conditioned media, ASCs were seeded in T75 tissue culture flasks at a density of 8000 cells/cm^2^, and incubated until approximately 70 % confluence (72 h). The cells were washed thoroughly with PBS to remove any albumin residues and 15 mL fresh StemPro E8 medium was added. Half of the flasks were cultured at 20 % oxygen, the other half at 1 % oxygen. After 24 h, the conditioned medium (CM) was collected, centrifuged, and decanted before protease inhibitors were added (1 tablet per 15 mL medium; Roche Complete Protease inhibitor cocktail, Mini). The resulting CM was first fractionated using spin filters into a high-molecular weight secretome fraction (>30 kDa) using a 30-kDa spinfilter (Millipore, Billerica, MA, USA), and, based on the flow-through, a low-molecular weight peptidome fraction (3–30 kDa), where molecules smaller than 3 kDa were removed using a 3-kDa spinfilter (Millipore). After both filtration steps, the retained proteins trapped on the spin filters were washed twice with 4 mL TEAB buffer (50 mM triethylamonium bicarbonate, pH 8.5), and retained in 500 μL TEAB buffer. The protein content was measured spectrophotometrically by protein OD A280 (Nanodrop; Thermo Science, Wilmington, DE), and the samples were stored at –80 °C for further analysis. All experiments were performed for all three cell lines in two separate experiments, each in duplicate.

After harvesting the CM, the ASCs were washed twice in PBS and the cells collected for proteome analysis using a protease and phosphatase inhibited RIPA buffer and subsequently sonicated to ensure complete lysis. Proteome samples were stored at –80 °C until further analysis.

### Sample preparation

#### Secretome

From each sample, a volume corresponding to 25 μg protein was transferred to an Eppendorf tube, and 50 mM TEAB buffer, pH 8.5, was added to a total volume of 100 μL. The proteins were reduced by the addition of 2 μl 0.5 M tris(2-carboxyethyl)phosphine (Thermo Scientific, Waltham, MA, USA) and incubation for 30 min at 37 °C. Next, the proteins were alkylated by the addition of 8 μl 0.5 M chloroacetamide (Sigma-Aldrich, St. Louis, MO, USA) and incubation for 30 min at 37 °C in the dark. Trypsin (0.5 μg) was added to each sample, and the proteins were digested overnight at 37 °C. The enzymatic procedure was stopped by addition of 5 μl 100 % formic acid. Protein digests were dried by vacuum centrifugation and desalted on PorosR3 nanocolumns and resuspended in 30 μL of a solution containing 2 % acetonitrile and 0.1 % formic acid.

#### Peptidome

The lower molecular weight proteins (3–30 kDa) were concentrated in YM-3 kDa spinfilter (Millipore, Billerica, MA, USA) by centrifugation at 14,000 g at 4 °C. The retained fraction (100 μL) was processed as described for the secretome samples.

#### Proteome

For the proteome analysis of the cells, a total of 25 μL of protein lysate in modified RIPA buffer was mixed with reducing sample buffer (2× Laemmeli; Bio-Rad, Hercules, CA) and isolated by reducing sodium dodecyl sulfate polyacrylamide gel electrophoresis (SDS-PAGE; (Biorad Any kD Mini-PROTEAN® TGX). The electrophoresis was stopped when the protein had entered 1.5 cm into the running gel according to the applied prestained protein marker (SeeBlue Plus2 pre-stained standard; Invitrogen, Carlsbad, CA). The protein load on each gel lane was comparable. The protein was visualized by Coomassie blue (SimplyBlue SafeStain; Invitrogen) and 50 % of each band excised and in-gel digested as described previously [49].

### Mass spectrometry analysis

The protein concentrations in the samples were normalized using A280 on a NanoDrop 1000 (Thermo Scientific, Wilmington, DE, USA), and 5 μg total peptide material was analyzed per LC-MS analysis of the low and high molecular weight proteins. A total of 20 % of each gel band was used for each of the biological replicates.

The samples were analyzed using a UPLC-nanoESI MS/MS setup with an UltiMate™ 3000 RSLC nanopump module. The system was coupled online with an emitter for nanospray ionization (New objective picotip 360-20-10) to a QExactive Plus mass spectrometer (Thermo Scientific, Waltham, USA). The peptide material was loaded onto a C18 reversed phase column (Dionex Acclaim PepMap RSLC C18, 2 μm, 100 Å, 100 μm × 2 cm) and separated using a C18 reversed phase column (Dionex Acclaim PepMap RSLC C18, 2 μm, 75 Å, 75 μm × 50 cm) at 40 °C. The sample was eluted with a gradient of 96 % solvent A (0.1 % FA) and 4 % solvent B (0.1 % FA in ACN), which was increased to 8 % solvent B on a 5-min ramp gradient and subsequently to 30 % solvent B on a 70-min ramp gradient for the proteome samples and 30 min for the secretome samples, at a constant flow rate of 300 nL/min. The mass spectrometer was operated in positive mode, selecting up to 12 precursor ions based on the highest intensity for HCD fragmenting.

### Data analysis

The reproducibility and variation of the amounts of peptide per sample were determined using Progenesis QJ for Proteomics (NonLinear/Waters, UK). A label-free quantitation by normalized XIC was performed of the trypsin-digested samples by searching the data files using MaxQuant 1.5.2.8 against the *Homo sapiens* Uniprot reference proteome database (UPID5640; April 2015). All standard settings were employed with carbamidomethyl (C) as a static modification and oxidation (M) as a variable modification. All proteins are reported at <1 % false discovery rate (FDR) to ensure only high-confidence protein identifications. The result file from MaxQuant was analyzed in Perseus v1.5.1.6. Initially, all reverse hits were removed from further analysis, and the data was log2-transformed. Two unique peptides or more were required for a protein identification and quantitation to ensure high-quality quantitation. Scatter plots were inspected biological-replicate wise. No replicates were removed due to low reproducibility. To further identify replicate outliers, a principle component analysis (PCA) was performed using all the measured protein abundances in all replicates as an input. For the purpose of conducting PCA, missing values (i.e., proteins where a quantitation value was not obtained for a given replicate analysis) were replaced with values from a normal distribution (width 0.3 and down shift 1.8) to simulate signals from low abundant proteins [50].

MS measurements were grouped preservation-method wise, and two-sided *t* tests were performed to identify statistically significant changing proteins. The statistical tests were corrected for multiple hypothesis testing using permutation-based FDR, and the parameters were chosen to provide sufficient input in the subsequent analysis (FDR = 0.05, s0 = 0.1, and 250 randomizations).

The genes coding for the significantly different expressed proteins found by MS were analyzed for statistical over-representation (enrichment) of Gene Ontology (GO) categories in terms of biological process using Cytoscape (version 3.3.0) with the Biological Networks GO plug-in (BINGO, version 3.0.3) [51, 52]. An adjusted *p* value of 0.05 was used as threshold for significance after correcting for multiple testing by Benjamini and Hochberg FDR correction. Over-representation was computed from hypergeometric test. The dataset for each donor was compared against a reference set of complete *Homo sapiens* GO annotations.

## Results

### Optimization of culture media

**Fig. 2:**
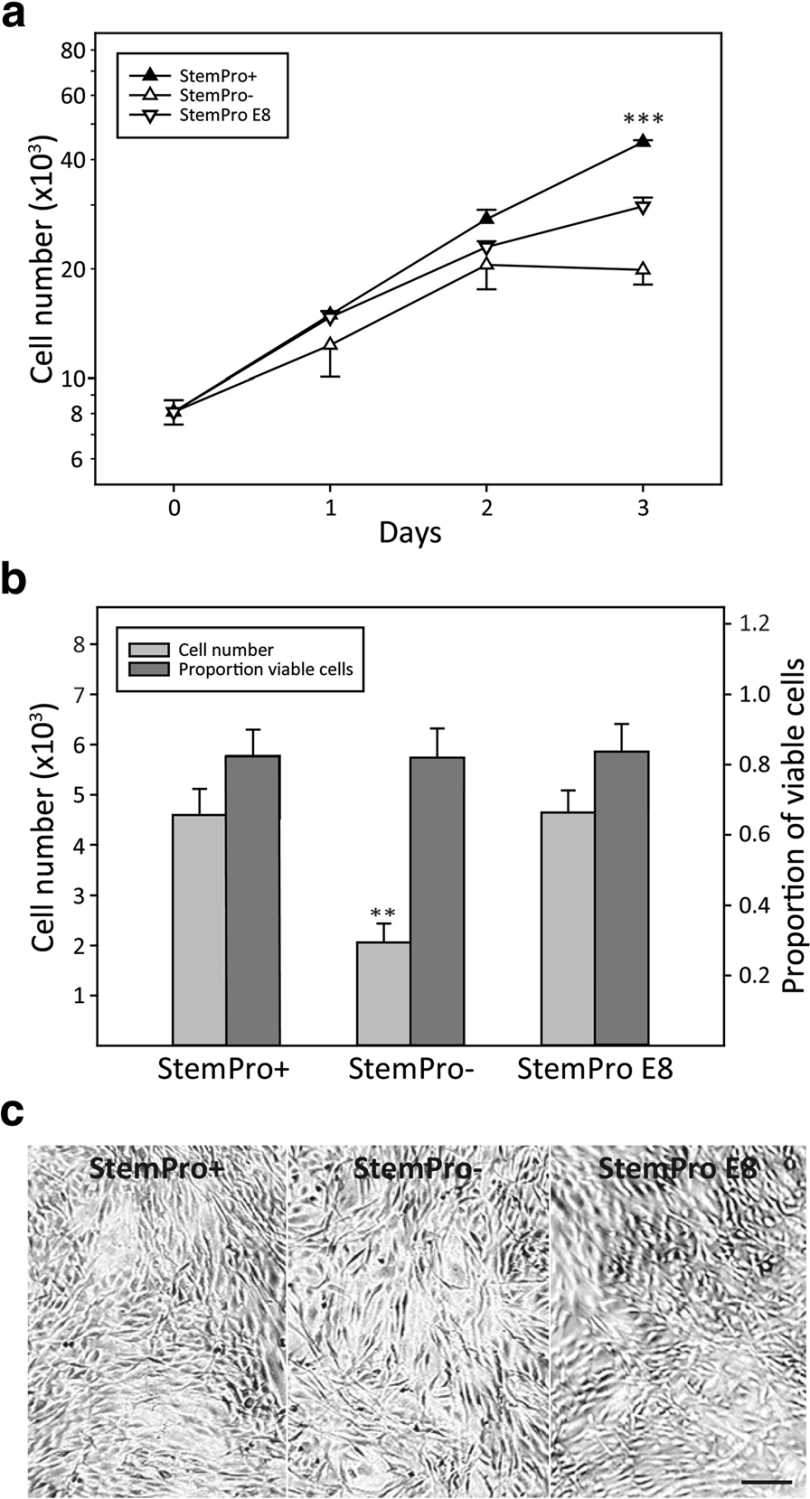
Effects of culture supplements on the growth and viability of the ASCs. **a** Growth curves of ASCs expanded for 3 days in the different media formulations. At day 3, there was a significantly higher number of cells in the StemPro + cultures as compared to the cultures with the other two media formulations (***p < 0.001, n = 6). **b** Cells expanded for 3 days in StemPro + and preconditioned for 24 h in the different media. After preconditioning, the number of cells in StemPro– cultures had significantly decreased in relation to the StemPro + cultures (**p < 0.01, n = 6). The percentage of viable cells was equivalent among the different formulations. **c** Phase contrast microphotographs displaying the morphology of cells after the preconditioning period in different media. The figure shows representative pictures from one donor. *Scale bar* = 200 μm

StemPro + has been shown to support ASC growth [53] but is not fully suitable for secretome analysis by MS due to the presence of large amounts of contaminant proteins, e.g., albumin. Low-protein and albumin-free media formulations include StemPro– and StemPro E8, which may offer an alternative for preconditioning of cells for MS. Initially, to compare the performance of the two media with StemPro+, ASCs were expanded in the three different media for up to 72 h. Analysis of the growth rate (Fig. 2a) revealed that StemPro + was superior in supporting a stable rate of cell growth, while in StemPro– cell growth is significantly inhibited over time. Although the growth rate of the cells in StemProE8 was comparable to StemPro + for the first 48 h, it decreased significantly after 72 h. Next, to assess the effect of short-term culture on cell number, viability, and morphology, ASCs were seeded at a higher density and grown in StemPro + for 72 h and then cultured in low-protein and albumin-free media formulations for 24 h. In terms of cell numbers, StemPro + and StemPro E8 were comparable, and StemPro– underperformed (Fig. 2b). Although analysis of cell viability did not suggest that culture in StemPro– caused compromised cell viability (Fig. 2b), cells cultured in StemPro– displayed a slightly more irregular morphology (Fig. 2c). Based on these results, ASCs were expanded in StemPro + and preconditioned in StemPro E8 for 24 h for the production of the CM and proteome samples for MS analysis.

### Identification of proteins

For ASC12, ASC21, and ASC23 cultured in normoxia and hypoxia, each of the quadruplicate biological replicates of secretome, peptidome, and proteome was processed as described above, separated and analyzed using UPLC-MS/MS. During this procedure, a list of the generated peptide fragments and information about the retention time, the accurate precursor ion mass and its ion intensity were obtained for each peptide. Using reverse-phase UPLC, the peptides were separated according to their hydrophobicity. When reaching the column, the peptides were ionized by electron spray and the mass of the peptide (precursor ion) was determined followed by fragmentation by collision and mass analysis of the resulting fragments. A good and reproducible separation of the peptides as well as a similar sample loading amount as determined by the total TIC was observed by plotting the m/z against the retention time of each peptide using Progenesis QI for Proteomics (data not shown).

The tandem mass spectra were used to search the Uniprot human reference proteome database with isoforms (UP000005640) to uniquely identify the parent proteins. Combining the MS1 spectra of the peptides and the MS2 spectra of their fragments, quantification of each protein was obtained. Validation of the UPLC-MS/MS spectra quantification was performed by plotting the intensities of proteins from each replicate against each other (Additional file 1: Figure S1). From this, a good correlation was found, increasing with increasing protein intensity. This is representative for all proteome samples with an average *R* = 0.98 ± 0.01.

### Effect of hypoxic treatment on the ASC secretome

In total, 342 proteins were identified in the secretome of ASC12, ASC21, or ASC23 by label-free quantitative data analysis using MaxQuant and post-processing in Perseus [27]. GO categories, by which at least three proteins were annotated, are shown in Additional file 2 (Table S1). The resulting GO profile showed that many of these proteins had functions that were related to developmental processes including angiogenesis and vasculature development, ECM and cell adhesion/migration, cell survival and cell death, as well as immune regulation. Interestingly, we did not detect any statistically significant hypoxia-induced up- or downregulation of proteins.

### Effect of hypoxic treatment on the ASC peptidome

In the peptidome fractions, only a total of 98 proteins were identified and quantified based on strict criteria. GO categories, by which at least three proteins were annotated, are shown in Additional file 3 (Table S2). The GO profile confirmed over-representation proteins with essentially the same functions as the proteins in the secretome. Of the 98 identified proteins, two were found significantly upregulated and three significantly downregulated in the ASC 23 cells (Additional file 4: Table S3). However, for the other ASCs we did not observe any significant effect of hypoxia on the MS peptidome profile.

### Effect of hypoxic treatment on the ASC proteome

**Fig. 3:**
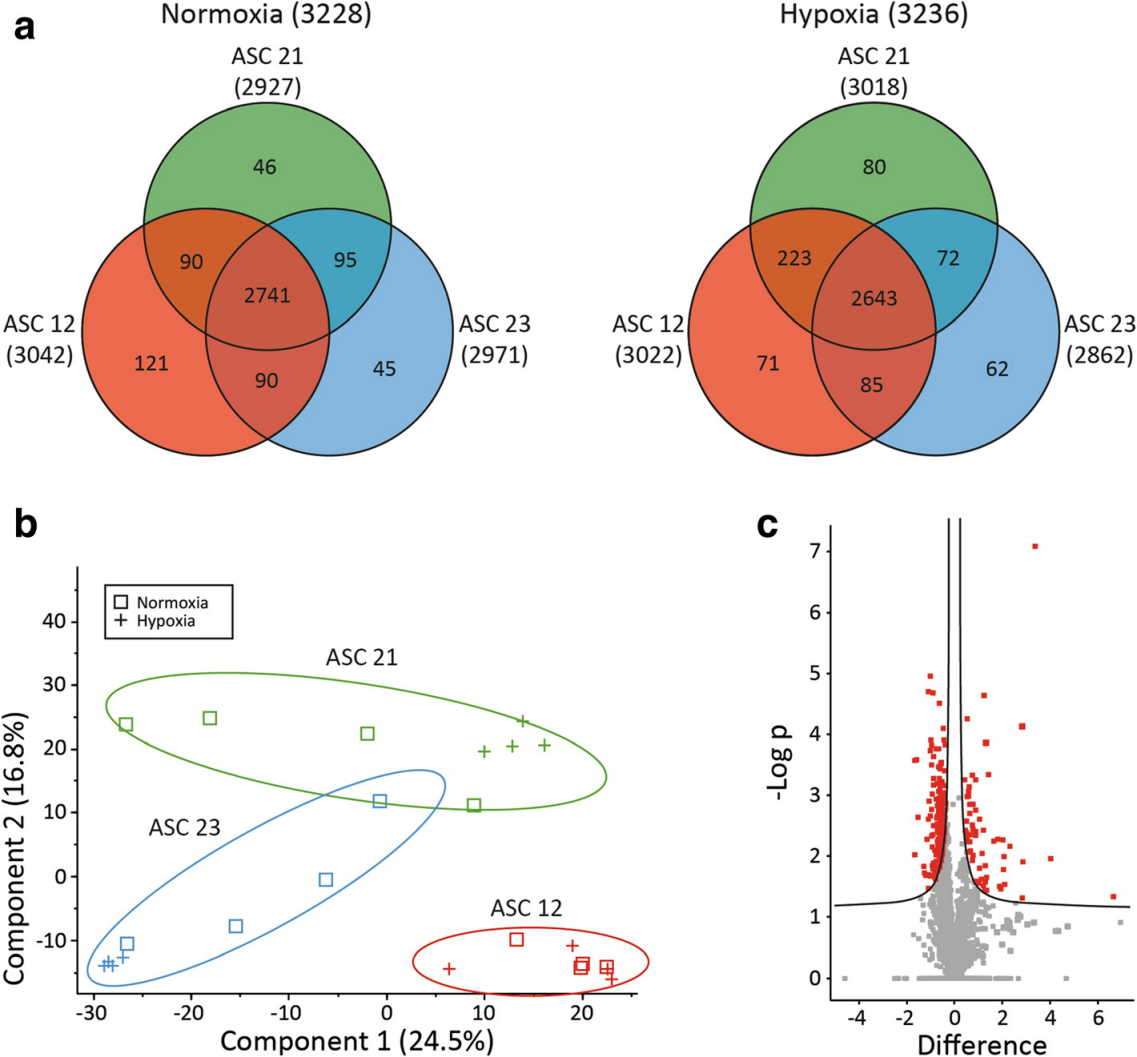
Analysis of the proteome of the ASCs by mass spectrometry. **a** Venn diagrams showing the concurrence in identified proteins in the proteome from the three donors (ASC 12, 21 and 23) exposed to either normoxic or hypoxic preconditioning. **b** Principal component analysis (PCA) of the proteome fractions in all samples. Shown are PCA score plots of principle components 1 and 2 of the protein abundances as measured in the normoxic and hypoxic samples from all three donors in four biological replicates. *Red*, ASC 12; *green*, ASC 21; *blue*, ASC 23; *square*, normoxic; *cross*, hypoxic. **c** Statistical analysis of the difference between label-free samples of the proteome fraction from hypoxic preconditioned ASC 21 against the proteome fraction from normoxic preconditioned ASC21 with a two-sided *t* test. The results are visualized by a scatter plot (volcano plot). The –log *t* test *p* value is plotted against the *t* test difference log2 for each protein. The proteins significantly changed between the samples (*p* < 0.05) are in the right and left upper corners (*red*). *ASC* adipose-derived stem cell

For cells cultured in normoxia, 94 % of proteins (2741 out of a total of 3228) were common to both ASC 12, ASC21, and ASC 23, with 50–100 proteins uniquely expressed by cells from just one donor (Fig. 3a). For the cells cultured in hypoxia, more than 85 % of proteins were detected in all cells. Despite the high degree of overlap between cells from different donors, the PCA revealed that the variations in protein expression between donors were larger than the variations caused by hypoxic treatment (Fig. 3b). The technical reproducibility of the MS setup used has previously been reported to be very high [54]. A representative volcano plot, depicting an analysis of the effect of hypoxia on protein expression, illustrates that the differences in protein levels between cells cultured in hypoxia and normoxia are less than two-fold for most proteins (Fig. 3c), and statistically significant for a only a relatively small fraction of proteins. Among the proteins regulated by hypoxic preconditioning, 235 (7.1 %) proteins were significantly downregulated, while 82 (2.5 %) were significantly upregulated.

### Biological significance of responses to hypoxia

To gain insight into the biological processes in which the differentially regulated proteins are involved, the genes coding for the proteins that were significantly differentially expressed were analyzed in terms of GO.

**Fig. 4:**
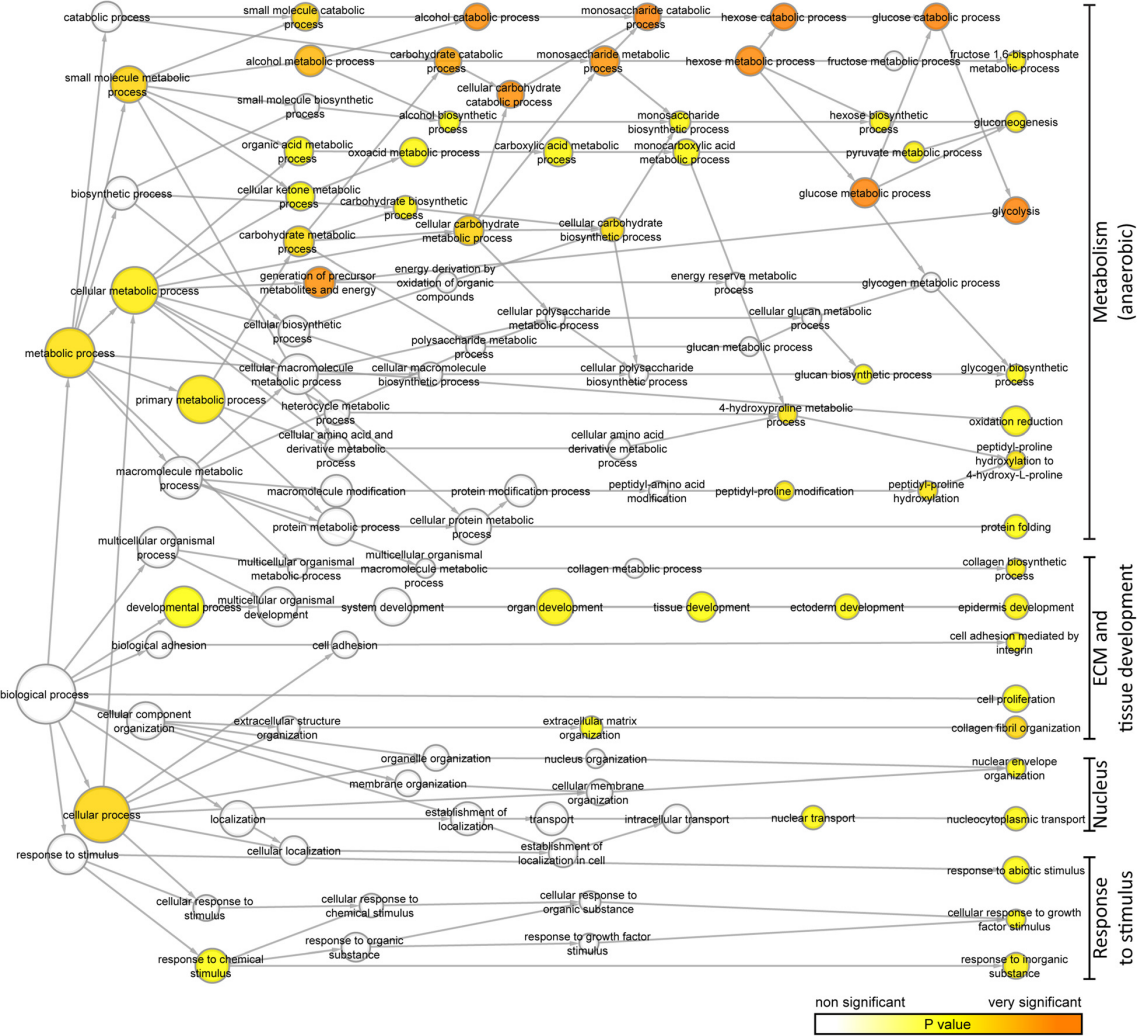
Gene ontology (GO) analysis of genes upregulated by hypoxic preconditioning of ASCs. The analysis was performed in Cytoscape using the BiNGO plug-in version 3.0.3. Presented is a reduced network showing all biological process categories that were significantly over-represented based on the genes corresponding to the upregulated proteins identified from all three donors. The color scale indicates the level of significance of the overrepresented GO category (adjusted *p* < 0.05). The size of the circles is proportional to the number of genes in each category. *ECM* extracellular matrix

The GO categories of the proteins identified as significantly upregulated by hypoxic preconditioning comprise several clusters of biological processes, including metabolic processes, cell adhesion and ECM, developmental processes, and responses to stimuli (Fig. 4). When looking into the specific upregulated processes (Table 1), not surprisingly the metabolic processes encompassed mostly those necessary for anaerobic metabolism. Also, processes related to cell adhesion and ECM were represented. Regarding the ECM and cell adhesion/migration, the upregulated proteins included prolyl 4-hydroxylases 1 and 2 (P4HA1 and 2), procollagen-lysine 2-oxoglutarate 5-dioxygenase 1 (PLOD1), and alpha-1 chains of collagen types 1, 3, and 7.

**Table 1:** Enriched biological processes based on upregulated proteins

Description	Genes involved	*p* value
Metabolism (anaerobic)		
Glycolysis	GPI, LDHA|TPI1, PGK1, ENO2, ALDOA, HK2, PFKP	0.0000
Gluconeogenesis	GPI, TPI1, ENO2	0.0101
Fructose 1,6-bisphosphate metabolic process	ALDOA, PFKP	0.0123
Glycogen biosynthetic process	GYS1, GBE1	0.0285
Oxidation reduction	LDHA, LOX, P4HA1, P4HA2, FTH1, CYP51A1 PLOD2, PLOD1, ERO1L, LOXL2, FTL	0.0245
Protein folding	LRPAP1, PFDN4, HSPBP1, PFDN6, ERO1L	0.0469
ECM and tissue development		
Collagen fibril organization	COL1A1, COL3A1, LOX, P4HA1	0.0010
Peptidyl-proline hydroxylation to 4-hydroxy-l-proline	P4HA1, P4HA2	0.0046
Collagen biosynthetic process	COL1A1, COL3A1	0.0123
Epidermis development	COL1A1, COL3A1, CRABP2, COL7A1, TXNIP, PLOD1	0.0181
Cell proliferation	CDV3, LRPAP1, NUMBL, CD81 ZAK, FTH1, APOA1, CD276	0.0474
Cell adhesion mediated by integrin	ITGA5, ICAM1	0.0255
Response to stimulus		
Response to inorganic substance	COL1A1, FNTA, BSG, TPM1, TXNIP, ACO1, NDRG1	0.0128
Cellular response to growth factor stimulus	COL1A1, EMD	0.0333
Response to abiotic stimulus	IKBIP, COL1A1, COL3A1, FECH ZAK, SLC2A1, TXNIP, ERO1L	0.0333
Nucleus		
Nuclear envelope organization	LMNA, EMD	0.0285
Nucleocytoplasmic transport	LSG1, LMNA, TXNIP, NUTF2, AGFG1	0.0285

*ECM* extracellular matrix

**Fig. 5:**
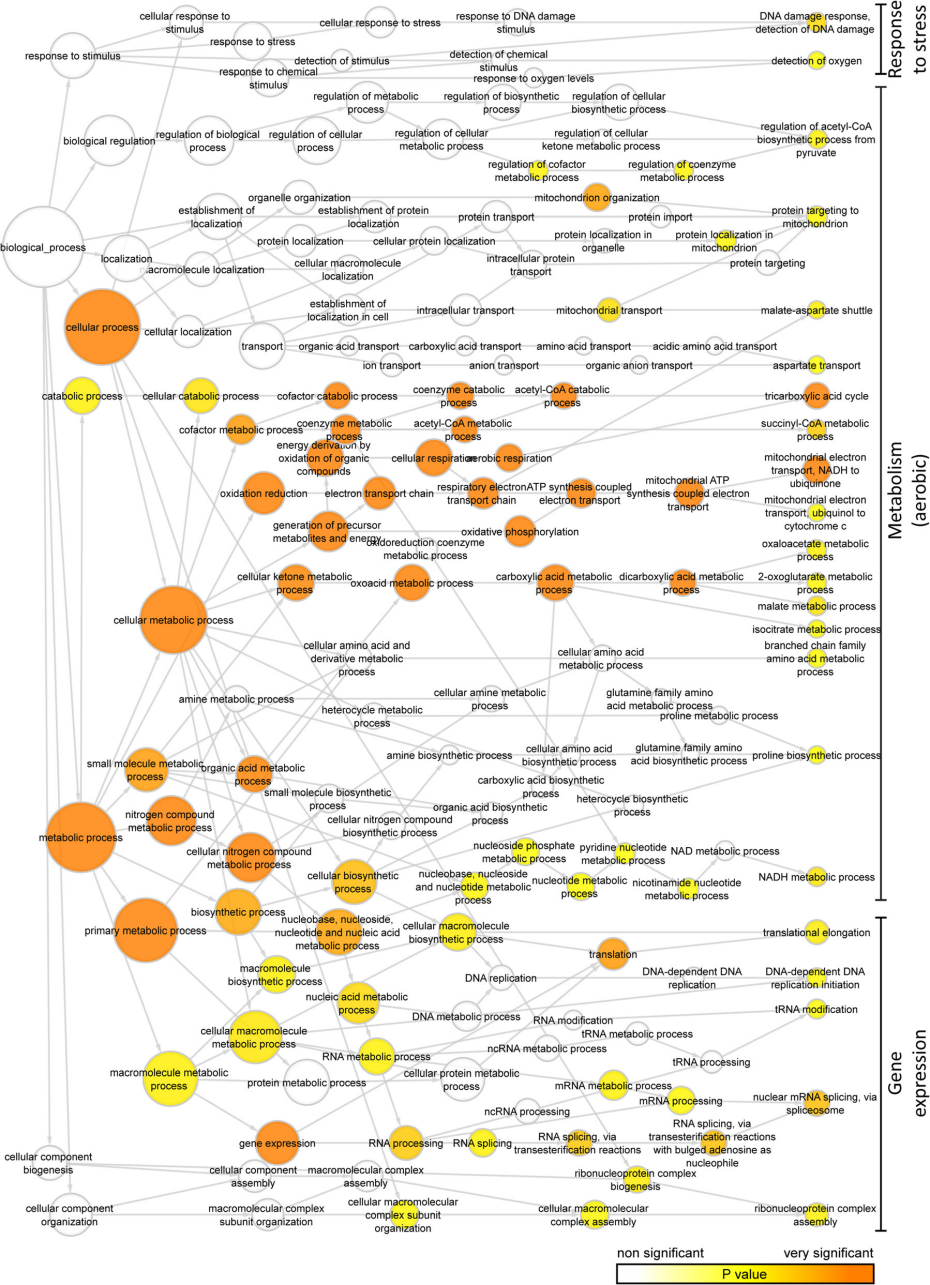
Gene ontology (GO) analysis of genes downregulated by hypoxic preconditioning of ASCs. The analysis was performed in Cytoscape using the BiNGO plug-in version 3.0.3. Presented is a reduced network showing all biological process categories that were significantly over-represented based on the genes corresponding to the downregulated proteins identified from all three donors. The color scale indicates the level of significance of the over-represented GO category (adjusted *p* < 0.05). The size of the circles is proportional to the number of genes in each category

When looking at the GO of proteins that were downregulated by the hypoxic preconditioning, the processes were clustered in terms of metabolism, protein synthesis, cell cycle, and stress response (Fig. 5). A closer look at those clusters (Table 2) revealed that the metabolic processes most downregulated were those related to aerobic metabolism thus complementing the pattern observed for the upregulated metabolic processes. Furthermore, several proteins involved in multiple steps in protein synthesis were downregulated, ranging from mRNA splicing and ribonucleoprotein complex assembly to translation elongation. Finally, the downregulated stress response proteins were related to detection of oxygen and DNA damage response.

**Table 2:** Enriched biological processes based on downregulated proteins

Description	Genes involved	*p* value
Metabolism (aerobic)		
Tricarboxylic acid cycle	CS, FH, SUCLA2, MDH2, IDH3G, SUCLG2 SUCLG1, ACO2, PDHB, IDH3A	0.0000
Succinyl-CoA metabolic process	SUCLA2, SUCLG2, SUCLG1	0.0004
mitochondrial electron transport, NADH to ubiquinone	NDUFA9, NDUFB9, NDUFA8, NDUFB8, NDUFS8, NDUFB10 NDUFS5, NDUFA10, NDUFB3, NDUFS3, DLD, NDUFV1	0.0000
Mitochondrial electron transport, ubiquinol to cytochrome c	UQCRC1, UQCR10	0.0335
2-Oxoglutarate metabolic process	IDH3G, DLD, IDH3A	0.0253
Malate metabolic process	FH, MDH2, ME2	0.0042
Isocitrate metabolic process	IDH3G, IDH3A	0.0335
Branched chain family amino acid metabolic process	HIBADH, HIBCH, BCAT2	0.0298
Proline biosynthetic process	PYCR1, PYCR2	0.0479
NADH metabolic process	MDH2, IDH3G, IDH3A	0.0060
Aspartate transport	SLC25A12, SLC25A13	0.0479
Malate-aspartate shuttle	SLC25A12, SLC25A13	0.0137
Regulation of acetyl-CoA biosynthetic process from pyruvate	PDP1, PDHB, DLD	0.0128
Oxaloacetate metabolic process	CS, MDH2, PCK2	0.0106
Protein targeting to mitochondrion	TOMM40, TOMM34, TIMM44, TOMM22	0.0198
Gene expression		
Nuclear mRNA splicing, via spliceosome	SF3B4, PRPF4, SF3A2, SNRPD1, DHX38 GEMIN5, USP39, WDR77, LSM2	0.0001
Translational elongation	GFM1, RPL21, RPL22, RPL13, RPL27, EEF2, TUFM	0.0099
DNA-dependent DNA replication initiation	MCM7, MCM3, MCM6	0.0253
tRNA modification	QTRT1, SSB, NSUN2	0.0335
Ribonucleoprotein complex assembly	SF3A2, SNRPD1, CIRBP, GEMIN5, USP39, WDR77	0.0147
Response to stress		
Detection of oxygen	SOD2, ENG	0.0335
DNA damage response, detection of DNA damage	MRPS9, PARP1, MRPS35	0.0017

## Discussion

When assessing the secretome of culture cells by MS the usual approach has been to deprive the cells of serum for a given period of time to avoid the presence of interfering proteins. In ASCs, however, serum deprivation has been shown to induce a stress response that might obscure the effect of the variable under experimental assessment, e.g., the oxygen tension [35, 55]. Here, as an alternative, we assessed a commercial serum-free medium that supports the expansion of ASCs. As high abundant proteins in the medium supplements, such as albumin, represent another source of interference which could dominate the mass spectrum and limit the detection of less abundant secreted proteins and peptides [56], two albumin-free formulations compatible with MS protein analysis were evaluated for the preconditioning phase.

To compare the secretome and proteome of ASCs cultured under hypoxic and normoxic conditions, ASCs were preconditioned in the MS-compatible medium for 24 h at 1 % and 20 % oxygen, after which the conditioned media and the cells were harvested. Subsequently, the conditioned media were fractioned into a secretome and a peptidome fraction and the cells were lysed to explore the proteome.

After analysis of the secreted proteins in both the secretome and the peptidome fraction, we identified a plethora of proteins relevant for stem cell maintenance and tissue regeneration. However, we were not able to detect a significant effect of hypoxic preconditioning on the abundance of these. Similarly, a recent report has shown that hypoxic preconditioning of ASCs does not seem to largely affect the secretion of proteins [57]. To decrease the complexity of the secretome, and thereby increase the chance of detecting low abundance proteins, we chose to fraction the conditioned media into two fractions: the secretome and the peptidome. However, this could be inadequate and further fractionation could have increased the sensitivity towards the low abundant proteins. It has been reported that the abundances of proteins in mammalian cells range from 1 to 10^7^ copies per cell [58, 59]. Global LC-MS/MS protein expression studies in cell lines have reported 50 % coverage of all predicted proteins for human cell lines, which is perceived as an excellent proteome coverage with today’s technology [58, 59]. To increase this in the future and enable the detection of quantitative changes for each protein across this range of abundances, extensive fractionation and one or more quantitative strategies are suggested [56]. For targeting of specific very low abundance transcription factors, cytokines, or chemokines, a targeted MS approach will enhance the applicability for detection and quantification [60].

Within the proteome of ASCs a variety of proteins were identified. Among these, a minor proportion was found to be differently expressed in ASCs exposed to hypoxic conditions. Among the most interesting findings, hypoxic conditioning of ASCs increased the presence of proteins involved in regulation of the ECM. Among these proteins were P4HA1 and P4HA2, which are required for collagen synthesis and are essential to the proper three-dimensional folding of newly synthesized procollagen chains [61], and PLOD1, which catalyzes the hydroxylation of lysyl residues in collagen-like peptides on the endoplasmatic reticulum and influences the stability of intermolecular collagen cross-links that provide the tensile strength and mechanical stability of collagen fibrils. Additionally, alpha-1 chains of collagen types 1, 3, and 7 were found to be upregulated. Collagen type 1 alpha chain 1 is a fibril-forming collagen found in most connective tissues and is abundant in the dermis. Collagen type 3 alpha chain 1 is also a fibril-forming collagen and is found in extensible connective tissues such as the skin and the vascular system, frequently in association with type I collagen. Collagen type 7 alpha chain 1 is restricted to the basement zone beneath stratified squamous epithelia. It functions as an anchoring fibril between the external epithelia and the underlying stroma, ensuring skin integrity and stability. It has been found that intradermal administration of mesenchymal stem cells resulted in production and deposition of collagen type 7 at the dermal-epidermal junction [62]. Among these proteins, P4HA1, P4HA2, PLOD 1, and collagen type 1 alpha chain 1 have been shown to be under transcriptional regulation in fibroblasts by hypoxia-inducible factor 1 (HIF-1) [61, 63]. As our preconditioning protocol has previously been shown to stabilize HIF-1 in these ASCs [64], the observed upregulation of the ECM-related genes could be mediated via HIF-1. This multifaceted upregulation of proteins involved in ECM production and modulation could contribute to a restoration of an ECM in chronic wounds that is conducive to healing, and thus partly explains the observed wound healing effects of hypoxic ASCs.

Besides the ECM-relevant proteins, we also found that hypoxic preconditioning significantly affected the metabolism of the ASCs. Proteins involved in anaerobic metabolism were found to be upregulated and proteins involved in aerobic metabolism were found to be downregulated. This is well described in the literature as a basic cellular response to hypoxic conditions, as cells during hypoxia rapidly enter a state of metabolic crisis which requires a fundamental shift in cellular metabolic strategy to facilitate the entering of an adaptive state which supports tissue survival [65]. This is very effective in ASCs, and hypoxic preconditioning has been shown to enhance the survival and regenerative potential of ASCs in vivo [14, 66–71].

Stress-related proteins were also downregulated by hypoxic conditioning. Among these were the poly(ADP-ribose) polymerase 1, which is involved in the regulation of various important cellular processes such as differentiation and proliferation. Others have found that 1 % O_2_ maintains stem cells in an undifferentiated state [72] and we have earlier shown that hypoxia at 1 % O_2_ decreases the proliferation of ASCs [16]. Poly(ADP-ribose) polymerase 1 could be involved in this.

Furthermore, proteins involved in protein translation were also found to be downregulated. Among these were proteins involved in mRNA splicing, ribonucleoprotein complex assembly and translation elongation as splicing factors binding to pre-mRNA, ribosomal proteins and the essential translation elongation factor 2 promoting the GTP-dependent translocation of the nascent protein chain from the A-site to the P-site of the ribosomes. It has previously been shown that moderate hypoxia of 1–0.1 % O_2_ affects gene expression, transcription, mRNA stability, protein synthesis and post-translational modifications [73]. The downregulation of the broad range of proteins related to translation that we observed in this study could therefore provide an explanation for the general decrease in protein synthesis by hypoxia.

Based on our findings and analysis, the explanation of the regenerative effect of ASCs was not to be found within the proteome. Despite the ECM-relevant proteins and a few others, the approach did not reveal differences in secretome abundance of other low abundant wound healing relevant factors. The majority of the proteins identified by MS were 1.11 ± 2.98-fold regulated and only a very small fraction of these was regulated by hypoxia. Earlier, we have shown hypoxic preconditioning to increase the level of VEGF two-fold as measured by ELISA which was found to be statistically significant compared to the normoxic levels of VEGF [64]. The analytical approach applied in this study allows for the discovery of unknown proteins as the data was compared to complete databases of all known proteins and their isoforms; however, the molecular size of multiple growth factors limits the detection by discovery proteomics. The detection of very low abundance cytokines and chemokines could be improved by targeted MS approaches such as MRM or PRM [60]. By combining our novel growth and fractionation approach, novel wound healing candidates could be discovered.

## Conclusions

In this study, we described a new serum-free culturing methodology to obtain conditioned medium from ASCs for MS analysis. Optimal cell growth, viability, and morphology were obtained using StemPro® SFM XenoFree basal medium containing the regular supplements during the expansion phase and, to avoid serum starvation and eliminate albumin interference, Essential 8™ supplements during the conditioning phase. While analysis of the secretome did not reveal any significant hypoxia-induced up- or downregulation of proteins, a relatively small fraction of the proteome was significantly affected by hypoxia. The main effects comprised ECM-relevant proteins ensuring tensile strength, three-dimensional folding, and mechanical stability of collagen fibrils in the dermis ensuring skin integrity and stability. Additionally, we found a switch in metabolism-relevant proteins indicating a change from aerobic to anaerobic metabolism. Although we could not find evidence supporting an enhanced secretion of pro-regenerative proteins by ASCs under hypoxia, this study provides a basis for further studies using proteomic techniques for the characterization of the ASC secretome.

### Availability of data

The mass spectrometry proteomics data have been deposited in the ProteomeXchange Consortium [74] via the PRIDE partner repository with the dataset identifier PXD003550.

## Additional files

Additional file 1: Figure S1. Scatter plots of all samples from the proteome fraction from all three donors. The log2 transformed protein abundance of all proteins obtained are plotted against each other on the *x*-axis and *y*-axis, respectively. Each spot represents the intensity of a protein. Ideally the measurements should yield identical protein abundances, represented by a Pearson’s correlation coefficient (*R*) of 1. (JPG 3314 kb) Additional file 2: Table S1. Over-represented biological processes by gene ontology analysis of proteins identified in the secretome fraction. (DOCX 63 kb) Additional file 3: Table S2. Over-represented biological processes by gene ontology analysis of proteins identified in the peptidome fraction. (DOCX 39 kb) Additional file 4: Table S3. Proteins in the peptidome fraction regulated by hypoxia. (DOCX 16 kb)

## Abbreviations

ASC: adipose-derived stem cell
CM: conditioned medium
ECM: extracellular matrix
ELISA: enzyme-linked immunosorbent assay
FCS: fetal calf serum
FDR: false discovery rate
GO: gene ontology
HIF-1: hypoxia-inducible factor 1
LC-MS/MS: liquid chromatography in line with a tandem mass spectrometry
MS: mass spectrometry
P4HA: prolyl 4-hydroxylase
PBS: phosphate-buffered saline
PCA: protein component analysis
PLOD1: procollagen-lysine 2-oxoglutarate 5-dioxygenase 1
TEAB: triethylammonium bicarbonate
VEGF: vascular endothelial growth factor

## References

[1] Mattar P, Bieback K (2015). Comparing the immunomodulatory properties of bone marrow, adipose tissue, and birth-associated tissue mesenchymal stromal cells. Front Immunol.

[2] Rasmussen JG, Frøbert O, Holst-Hansen C, Kastrup J, Baandrup U, Zachar V (2014). Comparison of human adipose-derived stem cells and bone marrow-derived stem cells in a myocardial infarction model. Cell Transplant.

[3] Sadat S, Gehmert S, Song Y-H, Yen Y, Bai X, Gaiser S (2007). The cardioprotective effect of mesenchymal stem cells is mediated by IGF-I and VEGF. Biochem Biophys Res Commun.

[4] Sawada K, Takedachi M, Yamamoto S, Morimoto C, Ozasa M, Iwayama T (2015). Trophic factors from adipose tissue-derived multi-lineage progenitor cells promote cytodifferentiation of periodontal ligament cells. Biochem Biophys Res Commun.

[5] Hassan WU, Greiser U, Wang W (2014). Role of adipose-derived stem cells in wound healing. Wound Repair Regen.

[6] Kapur SK, Katz AJ (2013). Review of the adipose derived stem cell secretome. Biochimie.

[7] Blazquez R, Sanchez-Margallo FM, de la Rosa O, Dalemans W, Alvarez V, Tarazona R (2014). Immunomodulatory potential of human adipose mesenchymal stem cells derived exosomes on in vitro stimulated T cells. Front Immunol.

[8] Pascucci L, Alessandri G, Dall’Aglio C, Mercati F, Coliolo P, Bazzucchi C (2014). Membrane vesicles mediate pro-angiogenic activity of equine adipose-derived mesenchymal stromal cells. Vet J.

[9] Lee SH, Jin SY, Song JS, Seo KK, Cho KH (2012). Paracrine effects of adipose-derived stem cells on keratinocytes and dermal fibroblasts. Ann Dermatol.

[10] Song YH, Shon SH, Shan M, Stroock AD, Fischbach C. Adipose-derived stem cells increase angiogenesis through matrix metalloproteinase-dependent collagen remodeling. Integr Biol (Camb). 2016;8(2):205-15. doi:10.1039/c5ib00277j. 10.1039/c5ib00277j PMC4755818 26758423

[11] Demidova-Rice TN, Hamblin MR, Herman IM (2012). Acute and impaired wound healing: pathophysiology and current methods for drug delivery, part 1: normal and chronic wounds: biology, causes, and approaches to care. Adv Skin Wound Care.

[12] Zachar V, Duroux M, Emmersen J, Rasmussen JG, Pennisi CP, Yang S (2011). Hypoxia and adipose-derived stem cell-based tissue regeneration and engineering. Expert Opin Biol Ther.

[13] Madrigal M, Rao KS, Riordan NH (2014). A review of therapeutic effects of mesenchymal stem cell secretions and induction of secretory modification by different culture methods. J Transl Med.

[14] Lee EY, Xia Y, Kim W-S, Kim MH, Kim TH, Kim KJ (2009). Hypoxia-enhanced wound-healing function of adipose-derived stem cells: increase in stem cell proliferation and up-regulation of VEGF and bFGF. Wound Repair Regen.

[15] Xu L, Wang X, Wang J, Liu D, Wang Y, Huang Z, et al. Hypoxia-induced secretion of IL-10 from adipose-derived mesenchymal stem cell promotes growth and cancer stem cell properties of Burkitt lymphoma. Tumour Biol. 2015. doi:10.1007/s13277-015-4664-8. 10.1007/s13277-015-4664-8 26695151

[16] Rasmussen JG, Frøbert O, Pilgaard L, Kastrup J, Simonsen U, Zachar V (2011). Prolonged hypoxic culture and trypsinization increase the pro-angiogenic potential of human adipose tissue-derived stem cells. Cytotherapy.

[17] He J, Cai Y, Luo L-M, Liu H-B (2015). Hypoxic adipose mesenchymal stem cells derived conditioned medium protects myocardial infarct in rat. Eur Rev Med Pharmacol Sci.

[18] Qin HH, Filippi C, Sun S, Lehec S, Dhawan A, Hughes RD (2015). Hypoxic preconditioning potentiates the trophic effects of mesenchymal stem cells on co-cultured human primary hepatocytes. Stem Cell Res Ther.

[19] Pilgaard L, Lund P, Duroux M, Lockstone H, Taylor J, Emmersen J (2009). Transcriptional signature of human adipose tissue-derived stem cells (hASCs) preconditioned for chondrogenesis in hypoxic conditions. Exp Cell Res.

[20] Dudakovic A, Camilleri E, Riester SM, Lewallen EA, Kvasha S, Chen X (2014). High-resolution molecular validation of self-renewal and spontaneous differentiation in clinical-grade adipose-tissue derived human mesenchymal stem cells. J Cell Biochem.

[21] Bath C, Muttuvelu D, Emmersen J, Vorum H, Hjortdal J, Zachar V (2013). Transcriptional dissection of human limbal niche compartments by massive parallel sequencing. PLoS One.

[22] Muers M (2011). Gene expression: transcriptome to proteome and back to genome. Nat Rev Genet.

[23] Vogel C, Marcotte EM (2012). Insights into the regulation of protein abundance from proteomic and transcriptomic analyses. Nat Rev Genet.

[24] Kupcova Skalnikova H (2013). Proteomic techniques for characterisation of mesenchymal stem cell secretome. Biochimie.

[25] Kasap M, Yeğenağa I, Akpinar G, Tuncay M, Aksoy A, Karaoz E (2015). Comparative proteome analysis of hAT-MSCs isolated from chronic renal failure patients with differences in their bone turnover status. PLoS One.

[26] Wiśniewski JR, Zougman A, Nagaraj N, Mann M (2009). Universal sample preparation method for proteome analysis. Nat Methods.

[27] Cox J, Hein MY, Luber CA, Paron I, Nagaraj N, Mann M (2014). Accurate proteome-wide label-free quantification by delayed normalization and maximal peptide ratio extraction, termed MaxLFQ. Mol Cell Proteomics.

[28] Frazier TP, Gimble JM, Kheterpal I, Rowan BG. Impact of low oxygen on the secretome of human adipose-derived stromal/stem cell primary cultures. Biochimie. 2013;95(12):2286-96. doi:10.1016/j.biochi.2013.07.011. 10.1016/j.biochi.2013.07.011 23880643

[29] Zvonic S, Lefevre M, Kilroy G, Floyd ZE, DeLany JP, Kheterpal I (2007). Secretome of primary cultures of human adipose-derived stem cells: modulation of serpins by adipogenesis. Mol Cell Proteomics.

[30] Lee MJ, Kim J, Kim MY, Bae Y, Ryu SH, Lee TG (2010). Proteomic analysis of tumor necrosis factor-alpha-induced secretome of human adipose tissue-derived mesenchymal stem cells. J Proteome Res.

[31] Eichelbaum K, Krijgsveld J (2014). Exocytosis and endocytosis.

[32] Kulkarni GV, McCulloch CA (1994). Serum deprivation induces apoptotic cell death in a subset of Balb/c 3 T3 fibroblasts. J Cell Sci.

[33] Endrich MM, Grossenbacher D, Geistlich A, Gehring H (1996). Apoptosis-induced concomitant release of cytosolic proteins and factors which prevent cell death. Biol Cell.

[34] Urs S, Turner B, Tang Y, Rostama B, Small D, Liaw L (2012). Effect of soluble Jagged1-mediated inhibition of Notch signaling on proliferation and differentiation of an adipocyte progenitor cell model. Adipocyte.

[35] Tratwal J, Mathiasen AB, Juhl M, Brorsen SK, Kastrup J, Ekblond A (2015). Influence of vascular endothelial growth factor stimulation and serum deprivation on gene activation patterns of human adipose tissue-derived stromal cells. Stem Cell Res Ther.

[36] Riis S, Zachar V, Boucher S, Vemuri MC, Pennisi CP, Fink T (2015). Critical steps in the isolation and expansion of adipose-derived stem cells for translational therapy. Expert Rev Mol Med.

[37] Patrikoski M, Juntunen M, Boucher S, Campbell A, Vemuri MC, Mannerström B (2013). Development of fully defined xeno-free culture system for the preparation and propagation of cell therapy-compliant human adipose stem cells. Stem Cell Res Ther.

[38] Yang S, Pilgaard L, Chase LG, Boucher S, Vemuri MC, Fink T (2012). Defined xenogeneic-free and hypoxic environment provides superior conditions for long-term expansion of human adipose-derived stem cells. Tissue Eng Part C Methods.

[39] Zachar V, Rasmussen JG, Fink T. Isolation and growth of adipose tissue-derived stem cells. In: Methods in molecular biology. Clifton, N.J.: The Royal Society of Chemistry, vol. 698. 2011. p. 37–49. 10.1007/978-1-60761-999-4_4 21431509

[40] Fink T, Rasmussen J, Lund P, Pilgaard L, Soballe K, Zachar V (2011). Isolation and expansion of adipose-derived stem cells for tissue engineering. Front Biosci (Elite Ed).

[41] Pilgaard L, Lund P, Rasmussen J, Fink T, Zachar V (2008). Comparative analysis of highly defined proteases for the isolation of adipose tissue-derived stem cells. Regen Med.

[42] Pilgaard L, Lund P, Duroux M, Fink T, Ulrich-Vinther M, Søballe K (2009). Effect of oxygen concentration, culture format and donor variability on in vitro chondrogenesis of human adipose tissue-derived stem cells. Regen Med.

[43] Andersen JI, Juhl M, Nielsen T, Emmersen J, Fink T, Zachar V (2014). Uniaxial cyclic strain enhances adipose-derived stem cell fusion with skeletal myocytes. Biochem Biophys Res Commun.

[44] Prasad M, Zachar V, Fink T, Pennisi CP (2014). Moderate hypoxia influences potassium outward currents in adipose-derived stem cells. PLoS One.

[45] Fink T, Lund P, Pilgaard L, Rasmussen JG, Duroux M, Zachar V (2008). Instability of standard PCR reference genes in adipose-derived stem cells during propagation, differentiation and hypoxic exposure. BMC Mol Biol.

[46] Foldberg S, Petersen M, Fojan P, Gurevich L, Fink T, Pennisi CP (2012). Patterned poly(lactic acid) films support growth and spontaneous multilineage gene expression of adipose-derived stem cells. Colloids Surf B Biointerfaces.

[47] Dominici M, Le Blanc K, Mueller I, Slaper-Cortenbach I, Marini F, Krause D (2006). Minimal criteria for defining multipotent mesenchymal stromal cells. The International Society for Cellular Therapy position statement. Cytotherapy.

[48] Pennisi CP, Dolatshahi-Pirouz A, Foss M, Chevallier J, Fink T, Zachar V (2011). Nanoscale topography reduces fibroblast growth, focal adhesion size and migration-related gene expression on platinum surfaces. Colloids Surf B Biointerfaces.

[49] Stensballe A, Andersen S, Jensen ON (2001). Characterization of phosphoproteins from electrophoretic gels by nanoscale Fe(III) affinity chromatography with off-line mass spectrometry analysis. Proteomics.

[50] Deeb SJ, D’Souza RCJ, Cox J, Schmidt-Supprian M, Mann M (2012). Super-SILAC allows classification of diffuse large B-cell lymphoma subtypes by their protein expression profiles. Mol Cell Proteomics.

[51] Maere S, Heymans K, Kuiper M (2005). BiNGO: a Cytoscape plugin to assess overrepresentation of gene ontology categories in biological networks. Bioinformatics.

[52] Shannon P, Markiel A, Ozier O, Baliga NS, Wang JT, Ramage D (2003). Cytoscape: a software environment for integrated models of biomolecular interaction networks. Genome Res.

[53] Riis S, Nielsen FM, Pennisi CP, Zachar V, Fink T (2016). Comparative analysis of media and supplements on initiation and expansion of adipose-derived stem cells. Stem Cells Transl Med.

[54] Bennike TB, Kastaniegaard K, Padurariu S, Gaihede M, Birkelund S, Andersen V (2016). Comparing the proteome of snap frozen, RNAlater preserved, and formalin-fixed paraffin-embedded human tissue samples. EuPA Open Proteomics.

[55] Follin B, Tratwal J, Haack-Sørensen M, Elberg JJ, Kastrup J, Ekblond A (2013). Identical effects of VEGF and serum-deprivation on phenotype and function of adipose-derived stromal cells from healthy donors and patients with ischemic heart disease. J Transl Med.

[56] Hawkridge AM. Practical considerations and current limitations in quantitative mass spectrometry-based proteomics. In: Quantitative proteomics. 2014. p. 1–25.

[57] Kalinina N, Kharlampieva D, Loguinova M, Butenko I, Pobeguts O, Efimenko A (2015). Characterization of secretomes provides evidence for adipose-derived mesenchymal stromal cells subtypes. Stem Cell Res Ther.

[58] Beck M, Schmidt A, Malmstroem J, Claassen M, Ori A, Szymborska A (2011). The quantitative proteome of a human cell line. Mol Syst Biol.

[59] Nagaraj N, Wisniewski JR, Geiger T, Cox J, Kircher M, Kelso J (2011). Deep proteome and transcriptome mapping of a human cancer cell line. Mol Syst Biol.

[60] Gallien S, Kim SY, Domon B (2015). Large-scale targeted proteomics using internal standard triggered-parallel reaction monitoring (IS-PRM). Mol Cell Proteomics.

[61] Gilkes DM, Bajpai S, Chaturvedi P, Wirtz D, Semenza GL (2013). Hypoxia-inducible factor 1 (HIF-1) promotes extracellular matrix remodeling under hypoxic conditions by inducing P4HA1, P4HA2, and PLOD2 expression in fibroblasts. J Biol Chem.

[62] Kühl T, Mezger M, Hausser I, Handgretinger R, Bruckner-Tuderman L, Nyström A (2015). High local concentrations of intradermal MSCs restore skin integrity and facilitate wound healing in dystrophic epidermolysis bullosa. Mol Ther.

[63] Deschene K, Céleste C, Boerboom D, Theoret CL (2012). Hypoxia regulates the expression of extracellular matrix associated proteins in equine dermal fibroblasts via HIF1. J Dermatol Sci.

[64] Rasmussen JG, Riis SE, Frøbert O, Yang S, Kastrup J, Zachar V (2012). Activation of protease-activated receptor 2 induces VEGF independently of HIF-1. PLoS One.

[65] Taylor CT (2008). Mitochondria and cellular oxygen sensing in the HIF pathway. Biochem J.

[66] Efimenko A, Starostina E, Kalinina N, Stolzing A (2011). Angiogenic properties of aged adipose derived mesenchymal stem cells after hypoxic conditioning. J Transl Med.

[67] Stubbs SL, Hsiao ST-F, Peshavariya HM, Lim SY, Dusting GJ, Dilley RJ (2012). Hypoxic preconditioning enhances survival of human adipose-derived stem cells and conditions endothelial cells in vitro. Stem Cells Dev.

[68] Liu L, Gao J, Yuan Y, Chang Q, Liao Y, Lu F (2013). Hypoxia preconditioned human adipose derived mesenchymal stem cells enhance angiogenic potential via secretion of increased VEGF and bFGF. Cell Biol Int.

[69] Rehman J, Traktuev D, Li J, Merfeld-Clauss S, Temm-Grove CJ, Bovenkerk JE (2004). Secretion of angiogenic and antiapoptotic factors by human adipose stromal cells. Circulation.

[70] Hollenbeck ST, Senghaas A, Komatsu I, Zhang Y, Erdmann D, Klitzman B (2012). Tissue engraftment of hypoxic-preconditioned adipose-derived stem cells improves flap viability. Wound Repair Regen.

[71] Park B-S, Kim W-S, Choi J-S, Kim H-K, Won J-H, Ohkubo F (2010). Hair growth stimulated by conditioned medium of adipose-derived stem cells is enhanced by hypoxia: evidence of increased growth factor secretion. Biomed Res.

[72] Lin Q, Lee Y-J, Yun Z (2006). Differentiation arrest by hypoxia. J Biol Chem.

[73] Ebbesen P, Eckardt K-U, Ciampor F, Pettersen EO (2004). Linking measured intercellular oxygen concentration to human cell functions. Acta Oncol.

[74] Vizcaíno JA, Deutsch EW, Wang R, Csordas A, Reisinger F, Ríos D (2014). ProteomeXchange provides globally coordinated proteomics data submission and dissemination. Nat Biotechnol.

