# Supplementary figures and images for: Mass spectrometry analysis of adipose-derived stem cells reveals a significant effect of hypoxia on pathways regulating extracellular matrix
Source: Stem Cell Res Ther. 2016 Apr 14;7:52. doi: 10.1186/s13287-016-0310-7 (PMC4831147; doi:10.1186/s13287-016-0310-7)

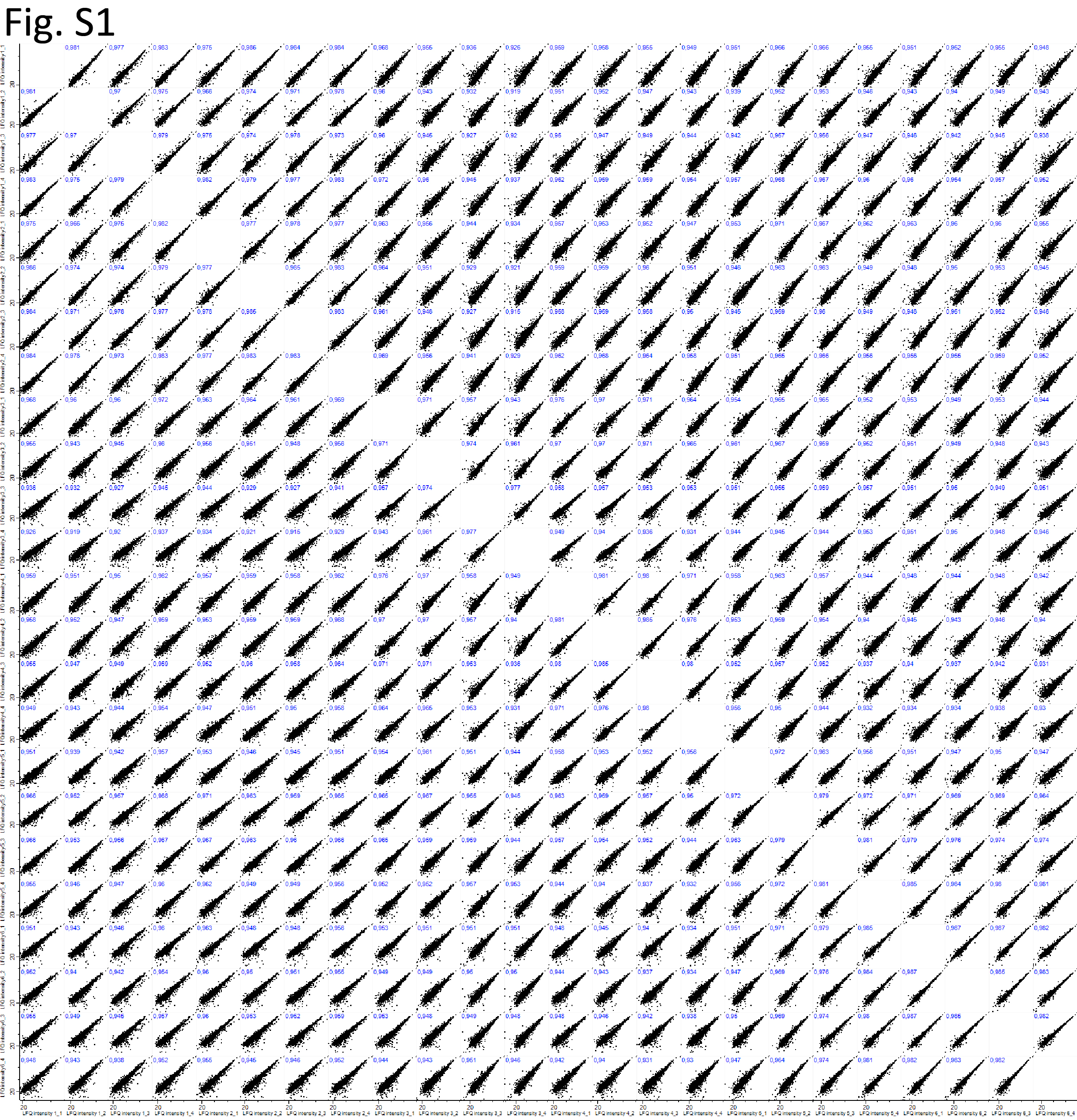

Supplement: Additional file 1: Figure S1. — Scatter plots of all samples from the proteome fraction from all three donors. The log2 transformed protein abundance of all proteins obtained are plotted against each other on the x-axis and y-axis, respectively. Each spot represents the intensity of a protein. Ideally the measurements should yield identical protein abundances, represented by a Pearson’s correlation coefficient (R) of 1. (JPG 3314 kb) [file 13287_2016_310_MOESM1_ESM.jpg]
