# Supplementary material for: Mass spectrometry analysis of adipose-derived stem cells reveals a significant effect of hypoxia on pathways regulating extracellular matrix
Source: Stem Cell Res Ther. 2016 Apr 14;7:52. doi: 10.1186/s13287-016-0310-7 (PMC4831147; doi:10.1186/s13287-016-0310-7)
Supplement: Additional file 2: Table S1. — Over-represented biological processes by gene ontology analysis of proteins identified in the secretome fraction. (DOCX 63 kb) [file 13287_2016_310_MOESM2_ESM.docx]

**Supplemental Table 1. Overrepresented biological processes by gene ontology analysis of proteins identified in the secretome fraction**

| Biological process | Genes coding for identified proteins (Gene names are given as HUGO gene nomenclature) | corr p-value |
| --- | --- | --- |
| system development | APP\|SPON2\|SPARC\|SERPINE2\|CSF1\|COL12A1\|SERPINE1\|TNC\|HP\|XYLT1\|PLOD1\|FGF2\|CLU\|CTGF\|COMP\|ALCAM\|LGALS1\|CDH2\|NAGLU\|BASP1\|ANPEP\|DPYSL2\|ARHGDIA\|DPYSL3\|CFL1\|DAG1\|POSTN\|IGFBP4\|KRT2\|SERPINF1\|KRT1\|TPM1\|MIF\|HSPG2\|KRT9\|DKK3\|MINPP1\|MYL6\|LOX\|NCL\|ADAM9\|PFN1\|SEMA7A\|LTBP2\|THY1\|AEBP1\|NID1\|PRDX3\|PRDX2\|PRDX1\|PDGFC\|PSAP\|NTNG1\|HSPA5\|FN1\|LIF\|INHBA\|COL1A1\|EXT1\|EXT2\|COL1A2\|BMP1\|COL5A1\|COL5A2\|TPP1\|CALR\|FBN1\|YWHAE\|GPI\|NRP1\|COL18A1\|ECM1\|HSP90AB1\|B4GALT1\|C2CD3\|HEXB\|CTSZ\|PCSK9\|LAMC2\|LAMC1\|ADAMTS4\|TIMP2\|SDF4\|ITGAV\|TIMP1\|YWHAG\|CTSB\|DSP\|ACTR3\|ANXA1\|GAA\|MMP2\|BGN\|PLAUR\|APOA1\|DCN\|VCAN\|RCN1\|CDH11\|CDH13\|COL6A3\|CD44\|TAGLN\|COL15A1\|LAMA2\|C1S\|SEMA3C\|GSTP1\|LAMA4\|THBS1\|FSTL3\|SRPX2\|LMNA\|SPOCK1\|GSN\|VEGFC\|LAMB1\|KRT10\|SOD2\|COL3A1\|AXL\|COL7A1\|KRT14\|PTX3\|AGRN\|FMOD | 9.91E-24 |
| anatomical structure development | APP\|SPON2\|SPARC\|SERPINE2\|CSF1\|COL12A1\|SERPINE1\|TNC\|HP\|XYLT1\|PLOD1\|FGF2\|CLU\|CTGF\|COMP\|ALCAM\|LGALS1\|CDH2\|NAGLU\|BASP1\|ANPEP\|DPYSL2\|ARHGDIA\|DPYSL3\|CFL1\|DAG1\|POSTN\|IGFBP4\|KRT2\|SERPINF1\|KRT1\|TPM1\|MIF\|HSPG2\|KRT9\|DKK3\|MINPP1\|MYL6\|LOX\|NCL\|ADAM9\|PFN1\|FBN2\|SEMA7A\|LTBP2\|THY1\|AEBP1\|NID1\|PRDX3\|PRDX2\|PRDX1\|PDGFC\|PSAP\|NTNG1\|HSPA5\|FN1\|LIF\|INHBA\|COL1A1\|EXT1\|EXT2\|COL1A2\|BMP1\|COL5A1\|COL5A2\|TPP1\|CALR\|FBN1\|YWHAE\|GPI\|NRP1\|COL18A1\|ECM1\|HSP90AB1\|B4GALT1\|C2CD3\|HEXB\|CTSZ\|HBB\|PCSK9\|LAMC2\|LAMC1\|ADAMTS4\|TIMP2\|SDF4\|ITGAV\|TIMP1\|YWHAG\|CTSB\|DSP\|ACTR3\|ANXA1\|GAA\|MMP2\|BGN\|PLAUR\|APOA1\|DCN\|RCN3\|VCAN\|RCN1\|CDH11\|CDH13\|COL6A3\|CD44\|TAGLN\|GRN\|COL15A1\|LAMA2\|C1S\|SEMA3C\|GSTP1\|LAMA4\|THBS1\|FSTL3\|SRPX2\|LMNA\|SPOCK1\|GSN\|VEGFC\|LAMB1\|KRT10\|SOD2\|COL3A1\|AXL\|COL7A1\|KRT14\|PTX3\|AGRN\|FMOD | 1.73E-22 |
| multicellular organismal development | APP\|SPON2\|SPARC\|SERPINE2\|CSF1\|COL12A1\|SERPINE1\|TNC\|HP\|XYLT1\|PLOD1\|FGF2\|CLU\|CTGF\|COMP\|ALCAM\|LGALS1\|CDH2\|NAGLU\|BASP1\|ANPEP\|DPYSL2\|ARHGDIA\|DPYSL3\|CFL1\|DAG1\|POSTN\|IGFBP4\|KRT2\|SERPINF1\|KRT1\|TPM1\|ADAM10\|PCOLCE\|MIF\|HSPG2\|KRT9\|DKK3\|MINPP1\|MYL6\|LOX\|NCL\|ADAM9\|PFN1\|FBN2\|SEMA7A\|LTBP2\|THY1\|AEBP1\|NID1\|PRDX3\|PRDX2\|PRDX1\|PDGFC\|PSAP\|IGFBP7\|NTNG1\|HSPA5\|FN1\|LIF\|INHBA\|COL1A1\|EXT1\|EXT2\|COL1A2\|BMP1\|COL5A1\|COL5A2\|TPP1\|CALR\|FBN1\|YWHAE\|GPI\|NRP1\|COL18A1\|ECM1\|HSP90AB1\|B4GALT1\|C2CD3\|HEXB\|CTSZ\|HBB\|PCSK9\|LAMC2\|LAMC1\|ADAMTS4\|TIMP2\|SDF4\|ITGAV\|TIMP1\|YWHAG\|CTSB\|DSP\|ACTR3\|ANXA1\|TPI1\|GAA\|MMP2\|BGN\|PLAUR\|APOA1\|DCN\|VCAN\|RCN1\|CDH11\|CDH13\|COL6A3\|ANGPTL4\|CD44\|TAGLN\|GRN\|COL15A1\|LAMA2\|C1S\|LRP1\|SEMA3C\|GSTP1\|LAMA4\|THBS1\|FSTL3\|SRPX2\|OLFML3\|LMNA\|SPOCK1\|GSN\|VEGFC\|LAMB1\|KRT10\|SOD2\|COL3A1\|AXL\|COL7A1\|KRT14\|PTX3\|AGRN\|FMOD | 3.21E-21 |
| developmental process | APP\|SPON2\|SPARC\|SERPINE2\|CSF1\|COL12A1\|SERPINE1\|TNC\|HP\|XYLT1\|PLOD1\|FGF2\|CLU\|CTGF\|LOXL2\|COMP\|ALCAM\|LGALS1\|CDH2\|NAGLU\|BASP1\|ANPEP\|DPYSL2\|ARHGDIA\|DPYSL3\|CFL1\|DAG1\|POSTN\|IGFBP4\|KRT2\|SERPINF1\|KRT1\|TPM1\|ADAM10\|PCOLCE\|MIF\|HSPG2\|KRT9\|DKK3\|MINPP1\|MYL6\|LOX\|PAPPA\|NCL\|ADAM9\|PFN1\|FBN2\|SEMA7A\|LTBP2\|THY1\|AEBP1\|NID1\|PRDX3\|PRDX2\|PRDX1\|PDGFC\|PSAP\|IGFBP7\|NTNG1\|HSPA5\|FN1\|LIF\|INHBA\|COL1A1\|EXT1\|EXT2\|TF\|COL1A2\|BMP1\|COL5A1\|COL5A2\|TPP1\|CALR\|FBN1\|YWHAE\|GPI\|NRP1\|COL18A1\|ECM1\|HSP90AB1\|B4GALT1\|C2CD3\|HEXB\|CTSZ\|HBB\|PCSK9\|LAMC2\|LAMC1\|ADAMTS4\|TIMP2\|SDF4\|ITGAV\|TIMP1\|CTSC\|YWHAG\|CTSB\|DSP\|ACTR3\|ANXA1\|TPI1\|ATP6AP1\|GAA\|MMP2\|BGN\|PLAUR\|APOA1\|DCN\|RCN3\|VCAN\|RCN1\|CDH11\|CDH13\|COL6A3\|ANGPTL4\|CD44\|TAGLN\|GRN\|COL15A1\|LAMA2\|C1S\|LRP1\|SEMA3C\|GSTP1\|LAMA4\|THBS1\|FSTL3\|SRPX2\|OLFML3\|LMNA\|SPOCK1\|GSN\|VEGFC\|LAMB1\|KRT10\|SOD2\|COL3A1\|AXL\|COL7A1\|KRT14\|PTX3\|AGRN\|FMOD | 7.07E-21 |
| cell adhesion | LGALS3BP\|APP\|SPON2\|NRP1\|COL18A1\|SRPX\|SPON1\|B4GALT1\|COL12A1\|CLSTN1\|TNC\|HP\|LAMC2\|LAMC1\|HAPLN1\|CTGF\|LOXL2\|WISP2\|COMP\|ISLR\|ALCAM\|CDH2\|DAG1\|EMILIN1\|ITGAV\|DSP\|POSTN\|ACTN1\|RPSA\|HSPG2\|VCAN\|COL6A2\|COL6A1\|CDH11\|ADAM9\|CDH13\|COL6A3\|TKT\|CD44\|COL15A1\|COL11A1\|THY1\|AEBP1\|NID1\|THBS2\|NID2\|THBS1\|FBLN5\|SRPX2\|SPOCK1\|IGFBP7\|FN1\|LAMB1\|MFAP4\|COL3A1\|BMP1\|COL5A1\|PTK7\|COL7A1\|ZYX\|ITGBL1 | 7.04E-20 |
| biological adhesion | LGALS3BP\|APP\|SPON2\|NRP1\|COL18A1\|SRPX\|SPON1\|B4GALT1\|COL12A1\|CLSTN1\|TNC\|HP\|LAMC2\|LAMC1\|HAPLN1\|CTGF\|LOXL2\|WISP2\|COMP\|ISLR\|ALCAM\|CDH2\|DAG1\|EMILIN1\|ITGAV\|DSP\|POSTN\|ACTN1\|RPSA\|HSPG2\|VCAN\|COL6A2\|COL6A1\|CDH11\|ADAM9\|CDH13\|COL6A3\|TKT\|CD44\|COL15A1\|COL11A1\|THY1\|AEBP1\|NID1\|THBS2\|NID2\|THBS1\|FBLN5\|SRPX2\|SPOCK1\|IGFBP7\|FN1\|LAMB1\|MFAP4\|COL3A1\|BMP1\|COL5A1\|PTK7\|COL7A1\|ZYX\|ITGBL1 | 7.04E-20 |
| organ development | APP\|CSF1\|SERPINE1\|TNC\|HP\|PLOD1\|FGF2\|CLU\|CTGF\|COMP\|CDH2\|BASP1\|ANPEP\|DPYSL2\|CFL1\|DAG1\|POSTN\|KRT2\|SERPINF1\|KRT1\|TPM1\|MIF\|HSPG2\|KRT9\|DKK3\|MINPP1\|MYL6\|LOX\|NCL\|ADAM9\|PFN1\|THY1\|AEBP1\|NID1\|PRDX3\|PRDX2\|HSPA5\|FN1\|LIF\|INHBA\|COL1A1\|EXT1\|EXT2\|COL1A2\|BMP1\|COL5A1\|COL5A2\|FBN1\|YWHAE\|GPI\|NRP1\|COL18A1\|ECM1\|HSP90AB1\|B4GALT1\|C2CD3\|CTSZ\|PCSK9\|LAMC2\|LAMC1\|SDF4\|ITGAV\|TIMP1\|CTSB\|DSP\|ANXA1\|GAA\|MMP2\|BGN\|PLAUR\|APOA1\|DCN\|RCN1\|CDH13\|COL6A3\|CD44\|TAGLN\|COL15A1\|LAMA2\|SEMA3C\|LAMA4\|THBS1\|FSTL3\|SRPX2\|LMNA\|GSN\|VEGFC\|LAMB1\|KRT10\|SOD2\|COL3A1\|AXL\|COL7A1\|KRT14\|PTX3\|AGRN\|FMOD | 1.54E-18 |
| extracellular matrix organization | APP\|COL18A1\|POSTN\|B4GALT1\|LUM\|COL11A1\|COL12A1\|LAMC1\|NID1\|DCN\|CTGF\|COL1A1\|CST3\|COL3A1\|CCDC80\|COL1A2\|LOX\|COL5A1\|COL6A2\|PXDN\|COL5A2\|SERPINH1\|EMILIN1\|TGFBI | 1.11E-16 |
| extracellular structure organization | APP\|COL18A1\|B4GALT1\|COL11A1\|COL12A1\|TNC\|LAMC1\|NID1\|CTGF\|CST3\|CDH2\|SERPINH1\|EMILIN1\|POSTN\|LUM\|DCN\|COL1A1\|COL3A1\|CCDC80\|COL1A2\|LOX\|COL5A1\|COL6A2\|PXDN\|COL5A2\|TGFBI\|AGRN | 3.68E-15 |
| multicellular organismal process | APP\|SPON2\|SPARC\|SERPINE2\|CSF1\|COL12A1\|SERPINE1\|TNC\|HP\|XYLT1\|PARK7\|PLOD1\|FGF2\|CLU\|CTGF\|COMP\|EFEMP2\|ALCAM\|PNP\|LGALS1\|EFEMP1\|CDH2\|NAGLU\|BASP1\|ANPEP\|DPYSL2\|ARHGDIA\|DPYSL3\|CFL1\|DAG1\|POSTN\|TPM4\|IGFBP4\|KRT2\|SERPINF1\|TPM2\|KRT1\|TPM1\|ADAM10\|PCOLCE\|MIF\|HSPG2\|KRT9\|DKK3\|PROCR\|MINPP1\|MYL6\|LOX\|NCL\|SERPING1\|ADAM9\|PFN1\|FBN2\|SEMA7A\|LTBP2\|THY1\|AEBP1\|NID1\|PRDX3\|PRDX2\|PRDX1\|PDGFC\|PSAP\|SERPINH1\|IGFBP7\|NTNG1\|CRTAP\|HSPA5\|FN1\|LIF\|INHBA\|COL1A1\|EXT1\|EXT2\|TF\|COL1A2\|BMP1\|COL5A1\|COL5A2\|TPP1\|CALR\|TGFBI\|FBN1\|YWHAE\|GPI\|NRP1\|COL18A1\|ECM1\|HSP90AB1\|B4GALT1\|C2CD3\|HEXB\|CTSZ\|HBB\|PCSK9\|LAMC2\|LAMC1\|ADAMTS4\|TIMP2\|SDF4\|ITGAV\|TIMP1\|YWHAG\|CTSB\|DSP\|ACTR3\|ANXA1\|TPI1\|MMP1\|GAA\|MMP2\|ANXA5\|ATP6AP2\|MMP3\|BGN\|PLAUR\|APOA1\|YWHAZ\|DCN\|VCAN\|RCN1\|CDH11\|CDH13\|COL6A3\|ANGPTL4\|ALDOA\|CD44\|TAGLN\|GRN\|COL15A1\|LAMA2\|C1S\|LRP1\|SEMA3C\|GSTP1\|COL11A1\|LAMA4\|THBS1\|FSTL3\|SRPX2\|OLFML3\|LMNA\|SPOCK1\|LDLR\|GSN\|LUM\|VEGFC\|LAMB1\|KRT10\|SOD2\|COL3A1\|AXL\|COL7A1\|KRT14\|PTX3\|AGRN\|FMOD | 3.68E-15 |
| tissue development | APP\|NRP1\|COL18A1\|ECM1\|B4GALT1\|C2CD3\|CSF1\|SERPINE1\|CTSZ\|TNC\|HP\|LAMC2\|PLOD1\|LAMC1\|FGF2\|CTGF\|BASP1\|CFL1\|DAG1\|CTSB\|DSP\|POSTN\|ANXA1\|KRT2\|GAA\|KRT1\|TPM1\|PLAUR\|MIF\|DCN\|KRT9\|MINPP1\|MYL6\|RCN1\|ADAM9\|PFN1\|CD44\|SEMA3C\|LMNA\|GSN\|LIF\|VEGFC\|KRT10\|INHBA\|COL1A1\|EXT1\|EXT2\|COL3A1\|COL1A2\|BMP1\|COL5A1\|COL7A1\|KRT14\|COL5A2\|AGRN | 7.42E-15 |
| response to wounding | NRP1\|ITIH4\|B4GALT1\|C1S\|C1R\|SERPINE1\|CFI\|TNC\|FGF2\|CLU\|THBS1\|CTGF\|C3\|PRDX2\|EFEMP2\|LGALS1\|CTSB\|DSP\|ANXA1\|GSN\|IGFBP4\|KRT1\|ANXA5\|TPM1\|FN1\|PLAUR\|MIF\|SOD2\|PRDX6\|YWHAZ\|DCN\|PROCR\|TF\|COL3A1\|VCAN\|LOX\|COL5A1\|SERPING1\|PTX3\|MASP1\|FMOD\|CFB\|CD44 | 1.81E-12 |
| blood vessel development | GPI\|NRP1\|COL18A1\|COL15A1\|B4GALT1\|SEMA3C\|LAMA4\|THY1\|FGF2\|THBS1\|CTGF\|SRPX2\|CDH2\|ANPEP\|ITGAV\|MMP2\|FN1\|LIF\|BGN\|VEGFC\|HSPG2\|COL1A1\|COL3A1\|COL1A2\|LOX\|COL5A1\|NCL\|CDH13\|CD44 | 2.14E-11 |
| anatomical structure morphogenesis | APP\|SPON2\|GPI\|NRP1\|COL18A1\|B4GALT1\|C2CD3\|CSF1\|SERPINE1\|CTSZ\|TNC\|HP\|HBB\|LAMC1\|FGF2\|CLU\|CTGF\|COMP\|ALCAM\|CDH2\|ANPEP\|CFL1\|DAG1\|DSP\|GAA\|MMP2\|TPM1\|BGN\|PLAUR\|MIF\|HSPG2\|DCN\|DKK3\|RCN3\|VCAN\|NCL\|CDH13\|PFN1\|CD44\|FBN2\|COL15A1\|SEMA3C\|THY1\|THBS1\|SRPX2\|NTNG1\|GSN\|HSPA5\|FN1\|LIF\|VEGFC\|LAMB1\|INHBA\|SOD2\|COL1A1\|EXT1\|EXT2\|COL1A2\|BMP1\|COL5A1\|AXL\|COL5A2\|TPP1\|PTX3\|FMOD | 3.14E-11 |
| vasculature development | GPI\|NRP1\|COL18A1\|COL15A1\|B4GALT1\|SEMA3C\|LAMA4\|THY1\|FGF2\|THBS1\|CTGF\|SRPX2\|CDH2\|ANPEP\|ITGAV\|MMP2\|FN1\|LIF\|BGN\|VEGFC\|HSPG2\|COL1A1\|COL3A1\|COL1A2\|LOX\|COL5A1\|NCL\|CDH13\|CD44 | 4.00E-11 |
| response to stress | LGALS3BP\|SPON2\|NRP1\|COL18A1\|PGLYRP2\|HSP90AB1\|B4GALT1\|SERPINE1\|TNC\|HP\|XYLT1\|PCSK9\|HSPB1\|PLOD2\|PARK7\|PLOD1\|FGF2\|CLU\|CTGF\|EFEMP2\|LGALS1\|CTSD\|CTSB\|DSP\|HSP90AA1\|ANXA1\|TPM4\|DCD\|IGFBP4\|MMP2\|KRT1\|ANXA5\|TPM1\|PLAUR\|ACTN4\|MIF\|YWHAZ\|DCN\|PROCR\|VCAN\|LOX\|PXDN\|SERPING1\|ADAM9\|ANGPTL4\|MASP1\|PFN1\|CFB\|CD44\|ITIH4\|VCP\|C1S\|C1R\|CFI\|THBS1\|HSP90B1\|C3\|PRDX3\|CST3\|PRDX2\|PRDX1\|SERPINH1\|IGFBP7\|GSN\|HSPA5\|FN1\|INHBA\|SOD2\|PRDX6\|SOD3\|TICRR\|COL1A1\|TF\|COL3A1\|COL5A1\|ALB\|TPP1\|PTX3\|FMOD | 5.06E-10 |
| cellular component movement | YWHAE\|APP\|SPON2\|NRP1\|B4GALT1\|SEMA3C\|HSPB1\|LAMC1\|FGF2\|THBS1\|CTGF\|ALCAM\|SRPX2\|CDH2\|ARHGDIA\|CFL1\|ENPP2\|SPOCK1\|CTHRC1\|ACTR3\|ANXA1\|TPM4\|KRT2\|TPM1\|FN1\|PLAUR\|VEGFC\|APOA1\|MIF\|EXT1\|VCAN\|COL5A1\|CAPZA1\|CDH13\|VIM\|CD44 | 1.02E-09 |
| response to chemical stimulus | COL18A1\|HSP90AB1\|SERPINE1\|TNC\|HP\|XYLT1\|PCSK9\|PLOD3\|HSPB1\|PLOD2\|PARK7\|PLOD1\|FGF2\|CLU\|CTGF\|PNP\|LGALS1\|DPYSL2\|CFL1\|DAG1\|ENPP2\|SDF4\|CTSC\|CTSB\|ACTR3\|HSP90AA1\|TPM4\|SERPINF1\|MMP2\|KRT1\|ANXA5\|TPM1\|MMP3\|PLAUR\|ADAM10\|ACTN4\|MIF\|YWHAZ\|DCN\|LOX\|COL6A2\|PXDN\|ADAM9\|CDH13\|ANGPTL4\|PFN1\|CD44\|VCP\|C1S\|SEMA3C\|THBS1\|HSP90B1\|PRDX3\|PRDX2\|PRDX1\|SERPINH1\|IGFBP7\|LDLR\|GSN\|HSPA5\|VEGFC\|GGH\|SOD2\|PRDX6\|SOD3\|COL1A1\|TF\|COL3A1\|KRT14 | 1.35E-09 |
| regulation of cellular component movement | NRP1\|COL18A1\|B4GALT1\|SERPINE2\|CSF1\|LAMA2\|LAMA4\|SERPINE1\|HP\|THY1\|FGF2\|THBS1\|SRPX2\|DAG1\|ENPP2\|ACTN1\|IGFBP3\|TPM1\|VEGFC\|ADAM10\|LAMB1\|ACTN4\|TF\|ADAM9\|CDH13 | 2.17E-09 |
| collagen fibril organization | COL1A1\|COL3A1\|COL1A2\|LOX\|COL5A1\|LUM\|COL11A1\|COL12A1\|COL5A2\|SERPINH1 | 7.83E-09 |
| cell-substrate adhesion | ACTN1\|FN1\|HP\|LAMB1\|THY1\|LAMC1\|NID1\|NID2\|FBLN5\|CTGF\|COL3A1\|DAG1\|ITGBL1\|ADAM9\|ITGAV\|CD44 | 1.56E-08 |
| regulation of cell adhesion | GSN\|SERPINE2\|CSF1\|LAMA2\|LAMA4\|SERPINE1\|TPM1\|ADAM10\|NID1\|THBS1\|FSTL3\|COL1A1\|LGALS1\|CCDC80\|ARHGDIA\|ADAM9\|CDH13\|EMILIN1\|TGFBI | 1.66E-08 |
| carbohydrate catabolic process | GPI\|PGLYRP2\|TPI1\|MDH1\|MDH2\|HEXB\|GAA\|TALDO1\|ENO1\|GNS\|LDHB\|LDHA\|PKM\|PGLS\|ALDOA\|GAPDH | 4.09E-08 |
| glucose catabolic process | LDHB\|GPI\|LDHA\|TPI1\|PKM\|MDH1\|MDH2\|TALDO1\|ENO1\|PGLS\|ALDOA\|GAPDH | 5.05E-08 |
| regulation of localization | NRP1\|COL18A1\|B4GALT1\|SERPINE2\|CSF1\|LAMA2\|LRP1\|LAMA4\|SERPINE1\|TNC\|HP\|PCSK9\|THY1\|FGF2\|THBS1\|C3\|SRPX2\|CDH2\|DPYSL2\|PRDX1\|ARHGDIA\|DAG1\|ENPP2\|FLNA\|ITGAV\|ANXA1\|ACTN1\|IGFBP3\|TPM1\|LIF\|VEGFC\|ADAM10\|APOA1\|LAMB1\|ACTN4\|MIF\|INHBA\|TF\|ADAM9\|CDH13\|PTX3\|CALR | 6.70E-08 |
| skeletal system development | ECM1\|SPARC\|HEXB\|COL12A1\|LTBP2\|AEBP1\|FGF2\|FSTL3\|CTGF\|COMP\|ADAMTS4\|PRDX1\|DSP\|POSTN\|IGFBP4\|MMP2\|COL1A1\|EXT1\|EXT2\|MINPP1\|COL3A1\|COL1A2\|BMP1\|COL5A2\|CDH11\|TPP1\|FBN1 | 7.64E-08 |
| protein maturation | C2CD3\|C1S\|C1R\|KRT1\|CFI\|ATP6AP2\|PCSK9\|CLU\|C3\|TF\|SERPINH1\|SERPING1\|CPE\|CALR\|MASP1\|CFB | 1.20E-07 |
| regulation of developmental process | NRP1\|ECM1\|B4GALT1\|SERPINE2\|CSF1\|LAMA2\|LAMA4\|SERPINE1\|HP\|XYLT1\|THY1\|FGF2\|CLU\|THBS1\|FSTL3\|C3\|CST3\|PRDX2\|PNP\|LGALS1\|SRPX2\|CDH2\|DPYSL2\|ARHGDIA\|TIMP2\|ITGAV\|YWHAG\|ACTR3\|SERPINF1\|IGFBP3\|KRT1\|FN1\|LIF\|VEGFC\|INHBA\|SOD2\|TF\|BMP1\|ADAM9\|CALR\|ANGPTL4\|ALDOA\|AGRN | 2.26E-07 |
| regulation of cell migration | COL18A1\|SERPINE2\|CSF1\|LAMA2\|IGFBP3\|LAMA4\|SERPINE1\|TPM1\|HP\|VEGFC\|ADAM10\|LAMB1\|THY1\|FGF2\|THBS1\|TF\|SRPX2\|DAG1\|ENPP2\|ADAM9\|CDH13 | 2.43E-07 |
| regulation of programmed cell death | YWHAE\|APP\|NRP1\|COL18A1\|VCP\|B4GALT1\|GSTP1\|SERPINE1\|HP\|PCSK9\|HSPB1\|FGF2\|CLU\|THBS1\|CTGF\|HSP90B1\|PRDX3\|COMP\|PRDX2\|LGALS1\|PRDX1\|ARHGDIA\|CFL1\|PSAP\|CTSB\|TXNDC5\|PDIA3\|ANXA1\|HSPA5\|ACTN1\|IGFBP3\|ANXA5\|ACTN4\|MIF\|INHBA\|SOD2\|PRDX6\|YWHAZ\|TF\|DDAH2\|ALB\|CDH13\|CALR\|ANGPTL4\|CD44 | 2.92E-07 |
| blood vessel morphogenesis | GPI\|NRP1\|COL18A1\|COL15A1\|B4GALT1\|SEMA3C\|MMP2\|FN1\|LIF\|BGN\|VEGFC\|THY1\|HSPG2\|FGF2\|THBS1\|CTGF\|SRPX2\|CDH2\|ANPEP\|NCL\|CDH13 | 3.17E-07 |
| hexose catabolic process | LDHB\|GPI\|LDHA\|TPI1\|PKM\|MDH1\|MDH2\|TALDO1\|ENO1\|PGLS\|ALDOA\|GAPDH | 3.17E-07 |
| regulation of cell death | YWHAE\|APP\|NRP1\|COL18A1\|VCP\|B4GALT1\|GSTP1\|SERPINE1\|HP\|PCSK9\|HSPB1\|FGF2\|CLU\|THBS1\|CTGF\|HSP90B1\|PRDX3\|COMP\|PRDX2\|LGALS1\|PRDX1\|ARHGDIA\|CFL1\|PSAP\|CTSB\|TXNDC5\|PDIA3\|ANXA1\|HSPA5\|ACTN1\|IGFBP3\|ANXA5\|ACTN4\|MIF\|INHBA\|SOD2\|PRDX6\|YWHAZ\|TF\|DDAH2\|ALB\|CDH13\|CALR\|ANGPTL4\|CD44 | 3.41E-07 |
| response to organic substance | VCP\|HSP90AB1\|C1S\|SERPINE1\|HP\|XYLT1\|PCSK9\|PLOD3\|HSPB1\|CLU\|THBS1\|CTGF\|HSP90B1\|PRDX3\|PRDX2\|LGALS1\|DPYSL2\|CFL1\|DAG1\|SERPINH1\|SDF4\|IGFBP7\|LDLR\|CTSC\|CTSB\|ACTR3\|HSP90AA1\|GSN\|HSPA5\|SERPINF1\|ANXA5\|MMP3\|ADAM10\|GGH\|MIF\|DCN\|COL1A1\|TF\|COL3A1\|LOX\|COL6A2\|ADAM9\|CDH13\|PFN1\|CD44 | 3.48E-07 |
| negative regulation of biological process | YWHAE\|NRP1\|COL18A1\|ECM1\|HSP90AB1\|B4GALT1\|SERPINE2\|SERPINE1\|HP\|XYLT1\|PCSK9\|HSPB1\|ENO1\|FGF2\|CLU\|WISP2\|COMP\|LGALS1\|YWHAQ\|BASP1\|ARHGDIA\|CFL1\|DAG1\|TIMP2\|ITGAV\|TIMP1\|CTSB\|ANXA1\|ACTN1\|SERPINF1\|IGFBP3\|KRT1\|ANXA5\|TPM1\|ADAM10\|APOA1\|ACTN4\|MIF\|YWHAZ\|DKK3\|PSMA5\|PSMA6\|PSMA3\|DDAH2\|SERPING1\|CDH13\|ANGPTL4\|MASP1\|CD44\|LRP1\|GSTP1\|HTRA1\|THY1\|THBS1\|FSTL3\|HSP90B1\|PRDX3\|CST3\|PRDX2\|NT5E\|FLNA\|IGFBP7\|IGFBP6\|TXNDC5\|GSN\|HSPA5\|LIF\|INHBA\|SOD2\|PRDX6\|COL1A1\|TF\|COL3A1\|CAPZA1\|ALB\|TPP1\|CALR\|TGFBI | 3.48E-07 |
| monosaccharide catabolic process | LDHB\|GPI\|LDHA\|TPI1\|PKM\|MDH1\|MDH2\|TALDO1\|ENO1\|PGLS\|ALDOA\|GAPDH | 4.10E-07 |
| activation of plasma proteins involved in acute inflammatory response | C3\|TF\|C1S\|C1R\|KRT1\|CFI\|SERPING1\|MASP1\|CLU\|CFB | 4.38E-07 |
| cellular carbohydrate catabolic process | GPI\|TPI1\|MDH1\|MDH2\|GAA\|TALDO1\|ENO1\|LDHB\|LDHA\|PKM\|PGLS\|ALDOA\|GAPDH | 4.55E-07 |
| regulation of apoptosis | YWHAE\|APP\|NRP1\|COL18A1\|VCP\|B4GALT1\|GSTP1\|SERPINE1\|HP\|PCSK9\|HSPB1\|CLU\|THBS1\|CTGF\|HSP90B1\|PRDX3\|COMP\|PRDX2\|LGALS1\|PRDX1\|ARHGDIA\|CFL1\|PSAP\|CTSB\|TXNDC5\|PDIA3\|ANXA1\|HSPA5\|ACTN1\|IGFBP3\|ANXA5\|ACTN4\|MIF\|INHBA\|SOD2\|PRDX6\|YWHAZ\|TF\|DDAH2\|ALB\|CDH13\|CALR\|ANGPTL4\|CD44 | 5.06E-07 |
| protein maturation by peptide bond cleavage | C1S\|C1R\|KRT1\|CFI\|ATP6AP2\|PCSK9\|CLU\|C3\|TF\|SERPING1\|CPE\|MASP1\|CFB | 5.06E-07 |
| glycolysis | LDHB\|GPI\|LDHA\|TPI1\|PKM\|MDH1\|MDH2\|ENO1\|ALDOA\|GAPDH | 5.10E-07 |
| angiogenesis | GPI\|NRP1\|COL18A1\|COL15A1\|B4GALT1\|MMP2\|FN1\|VEGFC\|THY1\|HSPG2\|FGF2\|THBS1\|CTGF\|SRPX2\|ANPEP\|NCL\|CDH13 | 7.03E-07 |
| protein metabolic process | APP\|FKBP10\|HSP90AB1\|B4GALT1\|C2CD3\|CD81\|RPLP1\|HEXB\|CTSZ\|HP\|PCSK9\|PLOD3\|PLOD2\|PLOD1\|FGF2\|CLU\|LOXL1\|LOXL2\|EEF1B2\|ADAMTS4\|UCHL1\|CTSL\|ANPEP\|CFL1\|RPLP2\|QSOX1\|CTSD\|CTSC\|RPS12\|CTSB\|DSP\|MANBA\|HSP90AA1\|ANXA1\|MMP1\|IGFBP3\|MMP2\|KRT1\|ATP6AP2\|MMP3\|BGN\|PLAUR\|ADAM10\|RPSA\|DCN\|PSMA5\|ADAM19\|PSMA6\|PSMA3\|LOX\|EEF1D\|NCL\|ERP29\|SERPING1\|ADAM9\|FKBP9\|MASP1\|TKT\|PPIB\|PPIA\|CFB\|PPIC\|VCP\|C1S\|C1R\|CFI\|HTRA1\|AEBP1\|NID1\|NID2\|THBS1\|HSP90B1\|C3\|HPX\|PRDX2\|SERPINH1\|IGHA1\|LDLR\|HSPA5\|FN1\|INHBA\|TICRR\|TF\|COL3A1\|BMP1\|PTK7\|AXL\|QPCT\|CPE\|TPP1\|CALR\|P4HB | 7.24E-07 |
| regulation of protein processing | C3\|SERPINE2\|SERPINE1\|SERPING1\|MASP1\|THBS1 | 7.35E-07 |
| regulation of protein maturation by peptide bond cleavage | C3\|SERPINE2\|SERPINE1\|SERPING1\|MASP1\|THBS1 | 7.35E-07 |
| cell-matrix adhesion | ACTN1\|HP\|THY1\|NID1\|NID2\|FBLN5\|CTGF\|COL3A1\|DAG1\|ITGBL1\|ADAM9\|ITGAV\|CD44 | 8.06E-07 |
| cell differentiation | YWHAE\|APP\|SPON2\|NRP1\|COL18A1\|B4GALT1\|SERPINE2\|CSF1\|TNC\|XYLT1\|PCSK9\|LAMC1\|FGF2\|CLU\|CTGF\|ALCAM\|LGALS1\|CDH2\|BASP1\|ANPEP\|DPYSL2\|ARHGDIA\|CFL1\|TIMP2\|SDF4\|TIMP1\|YWHAG\|DSP\|ACTR3\|ANXA1\|KRT2\|SERPINF1\|TPM1\|MIF\|VCAN\|RCN1\|PAPPA\|NCL\|ADAM9\|ANGPTL4\|CD44\|SEMA7A\|COL15A1\|C1S\|SEMA3C\|LAMA4\|THY1\|FSTL3\|PRDX3\|LMNA\|PSAP\|NTNG1\|GSN\|FN1\|LIF\|VEGFC\|LAMB1\|INHBA\|SOD2\|COL1A1\|EXT1\|EXT2\|BMP1\|KRT14\|PTX3\|CALR\|AGRN | 9.78E-07 |
| regulation of locomotion | COL18A1\|SERPINE2\|CSF1\|LAMA2\|IGFBP3\|LAMA4\|SERPINE1\|TPM1\|HP\|VEGFC\|ADAM10\|LAMB1\|THY1\|FGF2\|THBS1\|TF\|SRPX2\|DAG1\|ENPP2\|ADAM9\|CDH13 | 1.02E-06 |
| negative regulation of protein maturation by peptide bond cleavage | SERPINE2\|SERPINE1\|SERPING1\|MASP1\|THBS1 | 1.02E-06 |
| regulation of protein metabolic process | YWHAE\|APP\|VCP\|HSP90AB1\|SERPINE2\|C2CD3\|CSF1\|CD81\|SERPINE1\|HP\|PCSK9\|HSPB1\|FGF2\|THBS1\|CTGF\|C3\|HPX\|FLNA\|ITGAV\|TIMP1\|HSPA5\|IGFBP3\|ATP6AP2\|LIF\|PLAUR\|VEGFC\|APOA1\|MIF\|PSMA5\|PSMA6\|PSMA3\|SERPING1\|ADAM9\|CALR\|MASP1\|CD44 | 1.04E-06 |
| cellular developmental process | YWHAE\|APP\|SPON2\|NRP1\|COL18A1\|B4GALT1\|SERPINE2\|C2CD3\|CSF1\|TNC\|XYLT1\|PCSK9\|LAMC1\|FGF2\|CLU\|CTGF\|ALCAM\|LGALS1\|CDH2\|BASP1\|ANPEP\|DPYSL2\|ARHGDIA\|CFL1\|TIMP2\|SDF4\|TIMP1\|YWHAG\|DSP\|ACTR3\|ANXA1\|KRT2\|SERPINF1\|TPM1\|MIF\|VCAN\|RCN1\|PAPPA\|NCL\|ADAM9\|ANGPTL4\|CD44\|SEMA7A\|COL15A1\|C1S\|SEMA3C\|LAMA4\|THY1\|FSTL3\|PRDX3\|LMNA\|PSAP\|NTNG1\|GSN\|FN1\|LIF\|VEGFC\|LAMB1\|INHBA\|SOD2\|COL1A1\|EXT1\|EXT2\|BMP1\|KRT14\|PTX3\|CALR\|AGRN | 1.12E-06 |
| positive regulation of biological process | YWHAE\|APP\|NRP1\|COL18A1\|ECM1\|HSP90AB1\|B4GALT1\|SERPINE2\|CSF1\|CD81\|SERPINE1\|HP\|HBB\|PCSK9\|LAMC1\|FGF2\|CLU\|CTGF\|PNP\|LGALS1\|DPYSL2\|ARHGDIA\|CFL1\|EMILIN1\|TIMP1\|PDIA3\|ACTR3\|HSP90AA1\|ANXA1\|SERPINF1\|IGFBP3\|CLEC11A\|KRT1\|TPM1\|ATP6AP2\|ADAM10\|APOA1\|ACTN4\|MIF\|PSMA5\|PSMA6\|PSMA3\|CCDC80\|EEF1D\|SERPING1\|ADAM9\|CDH13\|ANGPTL4\|MASP1\|TKT\|CFB\|CD44\|GRN\|VCP\|C1S\|LRP1\|C1R\|CFI\|THY1\|NID1\|THBS1\|FSTL3\|C3\|PRDX3\|HPX\|SRPX2\|PRDX1\|PDGFC\|PSAP\|FLNA\|HSPA5\|LIF\|VEGFC\|LAMB1\|INHBA\|SOD2\|TF\|BMP1\|PTX3\|CALR\|AGRN | 1.14E-06 |
| negative regulation of cell death | GSTP1\|SERPINE1\|HSPB1\|FGF2\|CLU\|THBS1\|HSP90B1\|PRDX3\|COMP\|PRDX2\|ARHGDIA\|CFL1\|CTSB\|TXNDC5\|ANXA1\|HSPA5\|ANXA5\|MIF\|SOD2\|PRDX6\|YWHAZ\|TF\|DDAH2\|ALB\|CDH13\|ANGPTL4\|CD44 | 1.14E-06 |
| protein processing | C2CD3\|C1S\|C1R\|KRT1\|CFI\|ATP6AP2\|PCSK9\|CLU\|C3\|TF\|SERPING1\|CPE\|MASP1\|CFB | 1.16E-06 |
| acute inflammatory response | ITIH4\|B4GALT1\|C1S\|C1R\|KRT1\|CFI\|FN1\|CLU\|C3\|TF\|SERPING1\|MASP1\|CFB | 1.19E-06 |
| nervous system development | YWHAE\|APP\|SPON2\|NRP1\|SERPINE2\|C2CD3\|HEXB\|HP\|XYLT1\|PCSK9\|LAMC1\|FGF2\|CLU\|ALCAM\|LGALS1\|CDH2\|NAGLU\|DPYSL2\|ARHGDIA\|DPYSL3\|CFL1\|TIMP2\|SDF4\|YWHAG\|ACTR3\|SERPINF1\|VCAN\|RCN1\|NCL\|PFN1\|CD44\|SEMA7A\|C1S\|SEMA3C\|GSTP1\|THY1\|PDGFC\|PSAP\|SPOCK1\|NTNG1\|GSN\|HSPA5\|LIF\|VEGFC\|LAMB1\|INHBA\|SOD2\|EXT1\|TPP1\|PTX3\|CALR\|AGRN | 1.23E-06 |
| negative regulation of cellular process | YWHAE\|NRP1\|COL18A1\|HSP90AB1\|B4GALT1\|SERPINE2\|SERPINE1\|HP\|XYLT1\|PCSK9\|HSPB1\|ENO1\|FGF2\|CLU\|WISP2\|COMP\|LGALS1\|YWHAQ\|BASP1\|ARHGDIA\|CFL1\|DAG1\|TIMP2\|ITGAV\|TIMP1\|CTSB\|ANXA1\|ACTN1\|SERPINF1\|IGFBP3\|ANXA5\|TPM1\|ADAM10\|APOA1\|ACTN4\|MIF\|YWHAZ\|DKK3\|PSMA5\|PSMA6\|PSMA3\|DDAH2\|CDH13\|ANGPTL4\|CD44\|LRP1\|GSTP1\|HTRA1\|THY1\|THBS1\|FSTL3\|HSP90B1\|PRDX3\|CST3\|PRDX2\|IGFBP7\|IGFBP6\|TXNDC5\|GSN\|HSPA5\|LIF\|INHBA\|SOD2\|PRDX6\|COL1A1\|TF\|CAPZA1\|ALB\|TPP1\|CALR\|TGFBI | 1.52E-06 |
| epidermis development | DSP\|ANXA1\|KRT2\|KRT1\|LAMC2\|KRT10\|PLOD1\|INHBA\|KRT9\|CTGF\|COL1A1\|COL3A1\|COL1A2\|COL5A1\|COL7A1\|KRT14\|COL5A2\|ADAM9 | 1.84E-06 |
| alcohol catabolic process | LDHB\|GPI\|LDHA\|TPI1\|PKM\|MDH1\|MDH2\|TALDO1\|ENO1\|PGLS\|ALDOA\|GAPDH | 1.84E-06 |
| regulation of anatomical structure morphogenesis | ACTR3\|NRP1\|ECM1\|B4GALT1\|CSF1\|SERPINF1\|SERPINE1\|KRT1\|FN1\|HP\|XYLT1\|THY1\|FGF2\|THBS1\|C3\|CST3\|TF\|SRPX2\|CDH2\|ARHGDIA\|ADAM9\|ANGPTL4\|ALDOA | 2.02E-06 |
| response to stimulus | APP\|SPON2\|PGLYRP2\|SERPINE1\|TNC\|HP\|XYLT1\|PLOD3\|PLOD2\|ENO1\|PARK7\|PLOD1\|FGF2\|CLU\|CTGF\|IGHG1\|EFEMP2\|PNP\|LGALS1\|DPYSL2\|CFL1\|DAG1\|ENPP2\|PTGDS\|TPM4\|IGFBP4\|SERPINF1\|KRT1\|TPM1\|ADAM10\|ACTN4\|HLA-A\|MIF\|PROCR\|LOX\|SERPING1\|ADAM9\|MASP1\|PFN1\|CFB\|SEMA7A\|VCP\|CFI\|HSP90B1\|C3\|PRDX3\|PRDX2\|LDHA\|PRDX1\|SERPINH1\|IGFBP7\|IGHA1\|HSPA5\|FN1\|LIF\|GGH\|PRG4\|INHBA\|PRDX6\|COL1A1\|TF\|COL5A1\|ALB\|TPP1\|TGFBI\|LGALS3BP\|GPI\|NRP1\|COL18A1\|HSP90AB1\|B4GALT1\|HEXB\|PCSK9\|HSPB1\|SDF4\|CTSD\|CTSC\|CTSB\|DSP\|ACTR3\|HSP90AA1\|ANXA1\|DCD\|GAA\|MMP2\|ANXA5\|MMP3\|PLAUR\|YWHAZ\|DCN\|VCAN\|COL6A2\|PXDN\|CDH13\|ANGPTL4\|CD44\|ITIH4\|C1S\|SEMA3C\|C1R\|COL11A1\|THBS1\|CST3\|LDLR\|GSN\|VEGFC\|SOD2\|SOD3\|TICRR\|COL3A1\|AXL\|KRT14\|PTX3\|FMOD | 2.81E-06 |
| neurogenesis | YWHAE\|APP\|SPON2\|NRP1\|SERPINE2\|C1S\|XYLT1\|PCSK9\|THY1\|LAMC1\|FGF2\|CLU\|ALCAM\|LGALS1\|CDH2\|DPYSL2\|ARHGDIA\|PSAP\|TIMP2\|YWHAG\|NTNG1\|ACTR3\|GSN\|SERPINF1\|LIF\|VEGFC\|LAMB1\|SOD2\|EXT1\|VCAN\|NCL\|PTX3\|CALR\|AGRN\|CD44 | 2.81E-06 |
| complement activation | C3\|C1S\|C1R\|KRT1\|CFI\|SERPING1\|MASP1\|CLU\|CFB | 3.22E-06 |
| regulation of multicellular organismal process | APP\|NRP1\|ECM1\|B4GALT1\|SERPINE2\|CSF1\|LAMA2\|LAMA4\|SERPINE1\|TNC\|XYLT1\|PCSK9\|THY1\|FGF2\|THBS1\|FSTL3\|CTGF\|C3\|CST3\|PRDX2\|PNP\|LGALS1\|SRPX2\|CDH2\|DPYSL2\|ARHGDIA\|ANXA6\|TIMP2\|PTGDS\|YWHAG\|ACTR3\|SERPINF1\|GAA\|IGFBP3\|KRT1\|ANXA5\|TPM1\|ATP6AP2\|LIF\|VEGFC\|APOA1\|MIF\|INHBA\|TF\|BMP1\|CALR\|ANGPTL4\|AGRN | 4.41E-06 |
| wound healing | DSP\|GSN\|B4GALT1\|SERPINE1\|ANXA5\|TPM1\|PLAUR\|FGF2\|DCN\|PROCR\|TF\|COL3A1\|EFEMP2\|LOX\|COL5A1\|SERPING1\|FMOD\|CD44 | 4.56E-06 |
| anti-apoptosis | ANXA1\|HSPA5\|GSTP1\|ANXA5\|HSPB1\|SOD2\|CLU\|YWHAZ\|THBS1\|HSP90B1\|COMP\|PRDX2\|TF\|DDAH2\|ARHGDIA\|CFL1\|CDH13\|TXNDC5 | 4.56E-06 |
| response to reactive oxygen species | PRDX3\|COL1A1\|PRDX2\|TF\|PRDX1\|SERPINE1\|PXDN\|TPM1\|ADAM9\|PARK7\|SOD2\|PRDX6 | 5.02E-06 |
| ectoderm development | DSP\|ANXA1\|KRT2\|KRT1\|LAMC2\|KRT10\|PLOD1\|INHBA\|KRT9\|CTGF\|COL1A1\|COL3A1\|COL1A2\|COL5A1\|COL7A1\|KRT14\|COL5A2\|ADAM9 | 5.52E-06 |
| regulation of cellular component organization | NRP1\|SERPINE1\|HP\|XYLT1\|PCSK9\|THY1\|CLU\|THBS1\|C3\|CST3\|LGALS1\|CDH2\|ARHGDIA\|CFL1\|ACTR3\|ANXA1\|GSN\|TPM1\|FN1\|APOA1\|ACTN4\|MIF\|COL5A1\|CAPZA1\|ADAM9\|CDH13\|TPP1\|PTX3\|CALR\|ALDOA\|AGRN | 5.52E-06 |
| regulation of biological quality | APP\|GPI\|NRP1\|CSF1\|HEXB\|SERPINE1\|TNC\|HP\|HBB\|PCSK9\|ENO1\|PARK7\|FGF2\|EFEMP2\|CFL1\|DAG1\|QSOX1\|TIMP1\|YWHAG\|PDIA3\|ACTR3\|GAA\|ANXA5\|TPM1\|ATP6AP2\|PLAUR\|ADAM10\|APOA1\|YWHAZ\|PDIA4\|PROCR\|NCL\|SERPING1\|ANGPTL4\|ALDOA\|SLC3A2\|HSP90B1\|PRDX3\|HPX\|PRDX2\|PRDX1\|FLNA\|LDLR\|TXNDC5\|GSN\|TXNL1\|FN1\|VEGFC\|INHBA\|SOD2\|PRDX6\|TF\|COL3A1\|COL1A2\|CAPZA1\|ALB\|CPE\|TPP1\|CALR\|P4HB\|AGRN | 5.64E-06 |
| anatomical structure formation involved in morphogenesis | GPI\|NRP1\|COL18A1\|COL15A1\|B4GALT1\|SEMA3C\|HP\|THY1\|FGF2\|THBS1\|CTGF\|SRPX2\|ANPEP\|CFL1\|MMP2\|TPM1\|FN1\|VEGFC\|HSPG2\|COL1A1\|EXT2\|NCL\|CDH13\|PFN1\|CD44 | 6.26E-06 |
| negative regulation of apoptosis | GSTP1\|SERPINE1\|HSPB1\|CLU\|THBS1\|HSP90B1\|PRDX3\|COMP\|PRDX2\|ARHGDIA\|CFL1\|TXNDC5\|ANXA1\|HSPA5\|ANXA5\|MIF\|SOD2\|PRDX6\|YWHAZ\|TF\|DDAH2\|ALB\|CDH13\|ANGPTL4\|CD44 | 6.49E-06 |
| organ morphogenesis | NRP1\|COL18A1\|C2CD3\|CSF1\|SEMA3C\|CTSZ\|TNC\|HP\|THY1\|FGF2\|CTGF\|COMP\|CFL1\|DAG1\|DSP\|GAA\|MMP2\|TPM1\|LIF\|VEGFC\|LAMB1\|INHBA\|DCN\|COL1A1\|EXT2\|COL1A2\|BMP1\|COL5A1\|AXL\|COL5A2\|PTX3\|PFN1\|FMOD\|CD44 | 6.90E-06 |
| negative regulation of programmed cell death | GSTP1\|SERPINE1\|HSPB1\|CLU\|THBS1\|HSP90B1\|PRDX3\|COMP\|PRDX2\|ARHGDIA\|CFL1\|TXNDC5\|ANXA1\|HSPA5\|ANXA5\|MIF\|SOD2\|PRDX6\|YWHAZ\|TF\|DDAH2\|ALB\|CDH13\|ANGPTL4\|CD44 | 8.12E-06 |
| inflammatory response | ITIH4\|ANXA1\|B4GALT1\|C1S\|IGFBP4\|C1R\|KRT1\|CFI\|FN1\|MIF\|PRDX6\|CLU\|YWHAZ\|THBS1\|C3\|PRDX2\|TF\|SERPING1\|PTX3\|MASP1\|CFB\|CD44 | 1.36E-05 |
| generation of neurons | YWHAE\|APP\|SPON2\|NRP1\|SERPINE2\|XYLT1\|PCSK9\|THY1\|LAMC1\|CLU\|ALCAM\|LGALS1\|CDH2\|DPYSL2\|ARHGDIA\|PSAP\|TIMP2\|YWHAG\|NTNG1\|ACTR3\|SERPINF1\|LIF\|VEGFC\|LAMB1\|SOD2\|EXT1\|VCAN\|NCL\|PTX3\|CALR\|AGRN\|CD44 | 1.36E-05 |
| negative regulation of cell adhesion | COL1A1\|LGALS1\|SERPINE2\|ARHGDIA\|SERPINE1\|ADAM10\|CDH13\|TGFBI\|THBS1 | 1.40E-05 |
| cellular response to reactive oxygen species | PRDX3\|PRDX2\|TF\|PRDX1\|PXDN\|TPM1\|SOD2\|PRDX6 | 1.93E-05 |
| positive regulation of developmental process | ACTR3\|NRP1\|ECM1\|B4GALT1\|SERPINE2\|CSF1\|SERPINF1\|IGFBP3\|SERPINE1\|LIF\|VEGFC\|INHBA\|FGF2\|CLU\|THBS1\|C3\|TF\|PNP\|SRPX2\|BMP1\|ARHGDIA\|ADAM9\|ANGPTL4 | 1.93E-05 |
| peptide cross-linking | DSP\|COL3A1\|ANXA1\|FN1\|BGN\|THBS1\|DCN | 1.93E-05 |
| hydrogen peroxide metabolic process | PRDX3\|PRDX2\|PRDX1\|PXDN\|PARK7\|SOD2\|PRDX6 | 1.93E-05 |
| ossification | DSP\|ECM1\|SPARC\|MMP2\|FSTL3\|CTGF\|COL1A1\|EXT1\|EXT2\|MINPP1\|BMP1\|COL5A2\|CDH11 | 1.93E-05 |
| cell redox homeostasis | PRDX3\|PDIA3\|PRDX2\|PRDX1\|TXNL1\|QSOX1\|P4HB\|PRDX6\|PDIA4\|TXNDC5 | 2.01E-05 |
| response to hydrogen peroxide | PRDX3\|COL1A1\|PRDX2\|TF\|PRDX1\|PXDN\|ADAM9\|PARK7\|SOD2\|PRDX6 | 2.01E-05 |
| collagen biosynthetic process | COL1A1\|COL3A1\|COL5A1\|SERPINH1 | 2.47E-05 |
| defense response | LGALS3BP\|SPON2\|ITIH4\|PGLYRP2\|B4GALT1\|C1S\|C1R\|SERPINE1\|CFI\|HP\|CLU\|THBS1\|C3\|CST3\|PRDX2\|PRDX1\|ANXA1\|DCD\|IGFBP4\|KRT1\|FN1\|MIF\|INHBA\|PRDX6\|YWHAZ\|TF\|SERPING1\|PTX3\|MASP1\|PFN1\|CFB\|CD44 | 2.98E-05 |
| cell migration | YWHAE\|NRP1\|B4GALT1\|SEMA3C\|KRT2\|FN1\|VEGFC\|APOA1\|MIF\|LAMC1\|FGF2\|THBS1\|CTGF\|VCAN\|CDH2\|COL5A1\|CFL1\|CDH13\|CD44\|CTHRC1 | 3.33E-05 |
| positive regulation of cell adhesion | LGALS1\|CCDC80\|CSF1\|TPM1\|ADAM9\|CDH13\|EMILIN1\|NID1\|THBS1\|FSTL3 | 3.40E-05 |
| response to inorganic substance | GSN\|SERPINE1\|TPM1\|TNC\|GGH\|PARK7\|SOD2\|PRDX6\|THBS1\|SOD3\|PRDX3\|COL1A1\|PRDX2\|TF\|PRDX1\|PXDN\|KRT14\|ADAM9 | 4.50E-05 |
| localization of cell | YWHAE\|NRP1\|B4GALT1\|SEMA3C\|KRT2\|FN1\|VEGFC\|APOA1\|MIF\|LAMC1\|FGF2\|THBS1\|CTGF\|VCAN\|SRPX2\|CDH2\|COL5A1\|CFL1\|CDH13\|CD44\|CTHRC1 | 4.67E-05 |
| cell motility | YWHAE\|NRP1\|B4GALT1\|SEMA3C\|KRT2\|FN1\|VEGFC\|APOA1\|MIF\|LAMC1\|FGF2\|THBS1\|CTGF\|VCAN\|SRPX2\|CDH2\|COL5A1\|CFL1\|CDH13\|CD44\|CTHRC1 | 4.67E-05 |
| regulation of immune system process | CSF1\|C1S\|C1R\|SERPINE1\|CFI\|THY1\|CLU\|THBS1\|FSTL3\|C3\|HPX\|PRDX2\|PNP\|KRT1\|LIF\|VEGFC\|ADAM10\|APOA1\|MIF\|INHBA\|COL3A1\|SERPING1\|MASP1\|CFB\|CD44 | 4.72E-05 |
| negative regulation of cellular component movement | ACTN1\|IGFBP3\|SERPINE1\|TPM1\|DAG1\|HP\|ACTN4\|THY1\|FGF2\|THBS1 | 4.80E-05 |
| collagen metabolic process | COL1A1\|COL3A1\|COL5A1\|MMP1\|MMP2\|MMP3\|SERPINH1 | 4.84E-05 |
| positive regulation of cellular process | YWHAE\|APP\|NRP1\|COL18A1\|ECM1\|HSP90AB1\|B4GALT1\|SERPINE2\|CSF1\|CD81\|SERPINE1\|HP\|HBB\|PCSK9\|LAMC1\|FGF2\|CLU\|CTGF\|PNP\|LGALS1\|DPYSL2\|ARHGDIA\|CFL1\|EMILIN1\|TIMP1\|PDIA3\|ACTR3\|HSP90AA1\|ANXA1\|SERPINF1\|IGFBP3\|CLEC11A\|TPM1\|ADAM10\|APOA1\|ACTN4\|MIF\|PSMA5\|PSMA6\|PSMA3\|CCDC80\|EEF1D\|ADAM9\|CDH13\|TKT\|CD44\|GRN\|VCP\|THY1\|NID1\|THBS1\|FSTL3\|C3\|PRDX3\|HPX\|SRPX2\|PRDX1\|PDGFC\|PSAP\|FLNA\|HSPA5\|LIF\|VEGFC\|LAMB1\|INHBA\|SOD2\|TF\|PTX3\|CALR\|AGRN | 5.06E-05 |
| glycosaminoglycan metabolic process | EXT1\|EXT2\|ITIH4\|PGLYRP2\|HEXB\|BGN\|XYLT1\|GNS\|DCN | 5.15E-05 |
| positive regulation of angiogenesis | C3\|TF\|ECM1\|SRPX2\|SERPINE1\|ANGPTL4\|FGF2\|THBS1 | 5.30E-05 |
| immune effector process | C1S\|C1R\|KRT1\|CFI\|CLU\|YWHAZ\|C3\|PRDX2\|PRDX1\|SERPING1\|PTX3\|MASP1\|CFB | 5.46E-05 |
| carbohydrate metabolic process | GPI\|ITIH4\|PGLYRP2\|B4GALT1\|HEXB\|HEXA\|XYLT1\|SLC3A2\|ENO1\|GNS\|LDHB\|LDHA\|GANAB\|PGLS\|LDLR\|MANBA\|TPI1\|MDH1\|MDH2\|GAA\|BGN\|TALDO1\|DCN\|EXT1\|EXT2\|PKM\|ALDOA\|GAPDH | 6.32E-05 |
| bone development | DSP\|ECM1\|SPARC\|MMP2\|FSTL3\|CTGF\|COL1A1\|EXT1\|EXT2\|MINPP1\|BMP1\|COL5A2\|CDH11 | 6.37E-05 |
| regulation of response to stimulus | SERPINE2\|C1S\|C1R\|SERPINE1\|CFI\|XYLT1\|THY1\|CLU\|THBS1\|C3\|HPX\|PRDX2\|NT5E\|PRDX1\|PTGDS\|SERPINF1\|KRT1\|VEGFC\|ADAM10\|APOA1\|MIF\|TF\|COL3A1\|SERPING1\|CDH13\|MASP1\|CFB\|CD44 | 6.92E-05 |
| immune system process | SPON2\|GPI\|SEMA7A\|PGLYRP2\|B4GALT1\|CSF1\|C1S\|SEMA3C\|C1R\|CFI\|THY1\|CLU\|THBS1\|FSTL3\|C3\|PRDX3\|PRDX2\|IGHG1\|PNP\|PRDX1\|ENPP2\|TIMP1\|IGHA1\|CTSC\|KRT1\|LIF\|ADAM10\|PRG4\|HLA-A\|MIF\|INHBA\|SOD2\|YWHAZ\|PROCR\|PXDN\|SERPING1\|ADAM9\|PTX3\|CALR\|MASP1\|CFB | 7.23E-05 |
| regulation of cell proliferation | NRP1\|COL18A1\|ECM1\|GRN\|SPARC\|B4GALT1\|CSF1\|CD81\|SERPINE1\|HP\|LAMC1\|FGF2\|CLU\|THBS1\|CTGF\|WISP2\|PRDX3\|PNP\|PDGFC\|TIMP2\|IGFBP7\|TIMP1\|IGFBP6\|ANXA1\|SERPINF1\|IGFBP3\|CLEC11A\|LIF\|VEGFC\|ADAM10\|LAMB1\|MIF\|INHBA\|SOD2\|TF\|CDH13\|CALR\|TKT | 7.74E-05 |
| cellular process | APP\|SPON2\|SPON1\|PGLYRP2\|SERPINE2\|COL12A1\|SERPINE1\|XYLT1\|ENO1\|ISLR\|ALCAM\|PNP\|LGALS1\|ANPEP\|DPYSL2\|DPYSL3\|DAG1\|PTGDS\|RPS12\|KRT2\|ACTN1\|SERPINF1\|TALDO1\|ACTN4\|MIF\|HSPG2\|KRT9\|MYL6\|CCDC80\|PAPPA\|PFN1\|THY1\|PRDX3\|HPX\|LDHB\|PRDX2\|LDHA\|PRDX1\|SERPINH1\|IGHA1\|NTNG1\|FN1\|LIF\|PRDX6\|COL1A1\|EXT1\|EXT2\|TF\|COL1A2\|HNRNPK\|PTK7\|ITGBL1\|CALR\|GPI\|NRP1\|COL18A1\|SRPX\|FKBP10\|C2CD3\|RPLP1\|HEXB\|PCSK9\|UCHL1\|TIMP2\|RPLP2\|SDF4\|QSOX1\|TIMP1\|CTSD\|CTSB\|MANBA\|ANXA1\|TPI1\|GAA\|BGN\|PSMA5\|PSMA6\|PSMA3\|PXDN\|ANGPTL4\|TKT\|ALDOA\|PTMA\|CD44\|COL11A1\|FSTL3\|CST3\|NT5E\|SRPX2\|LMNA\|FLNA\|LDLR\|GSN\|MDH1\|MDH2\|VEGFC\|COL3A1\|AXL\|QPCT\|CAPZA1\|HNRNPA2B1\|KRT14\|ZYX\|AGRN\|FMOD\|CSF1\|CD81\|CLSTN1\|TNC\|HP\|PLOD3\|PLOD2\|PARK7\|PLOD1\|FGF2\|CLU\|LOXL1\|CTGF\|LOXL2\|COMP\|EEF1B2\|CDH2\|BASP1\|ARHGDIA\|CFL1\|ENPP2\|PDIA3\|POSTN\|TPM4\|IGFBP4\|IGFBP3\|TPM1\|ADAM10\|RPSA\|PDIA4\|MINPP1\|LOX\|NCL\|ERP29\|FSCN1\|ADAM9\|FKBP9\|PPIB\|PPIA\|PPIC\|SEMA7A\|VCP\|LTBP2\|AEBP1\|NID1\|NID2\|FBLN5\|HSP90B1\|PSAP\|IGFBP7\|TXNDC5\|HSPA5\|RARRES2\|GGH\|INHBA\|BMP1\|COL5A1\|COL5A2\|ALB\|CPE\|TPP1\|TGFBI\|YWHAE\|LGALS3BP\|MOXD1\|HSP90AB1\|B4GALT1\|HSPB1\|LAMC2\|LAMC1\|HAPLN1\|WISP2\|YWHAQ\|EMILIN1\|ITGAV\|PGLS\|YWHAG\|DSP\|ACTR3\|HSP90AA1\|ATP6AP1\|PLAUR\|APOA1\|REXO2\|YWHAZ\|DCN\|VCAN\|RCN1\|PKM\|EEF1D\|DDAH2\|COL6A2\|COL6A1\|CDH11\|CDH13\|COL6A3\|GAPDH\|COL15A1\|C1S\|LRP1\|SEMA3C\|LAMA4\|SLC3A2\|THBS2\|THBS1\|SPOCK1\|CTHRC1\|LUM\|TXNL1\|LAMB1\|SOD2\|SOD3\|TICRR\|MFAP4\|PSAT1\|COL7A1\|PTX3\|P4HB\|VIM | 7.89E-05 |
| humoral immune response | C3\|GPI\|C1S\|C1R\|KRT1\|CFI\|SERPING1\|MASP1\|CLU\|CFB | 7.96E-05 |
| regulation of cell development | ACTR3\|NRP1\|B4GALT1\|SERPINE2\|SERPINF1\|IGFBP3\|XYLT1\|VEGFC\|THY1\|FGF2\|LGALS1\|CDH2\|DPYSL2\|ARHGDIA\|TIMP2\|CALR\|AGRN\|YWHAG | 8.09E-05 |
| multicellular organismal macromolecule metabolic process | COL1A1\|COL3A1\|COL5A1\|MMP1\|MMP2\|MMP3\|SERPINH1 | 8.47E-05 |
| positive regulation of cellular component movement | COL18A1\|CSF1\|SERPINE1\|VEGFC\|ADAM10\|LAMB1\|ACTN4\|FGF2\|THBS1\|TF\|SRPX2\|ADAM9\|CDH13 | 9.75E-05 |
| skin development | COL1A1\|DSP\|COL3A1\|COL1A2\|COL5A1\|COL5A2\|KRT9 | 1.03E-04 |
| response to oxidative stress | TPM4\|SERPINE1\|KRT1\|TPM1\|PARK7\|SOD2\|PRDX6\|CLU\|PRDX3\|COL1A1\|PRDX2\|TF\|PRDX1\|PXDN\|ADAM9 | 1.09E-04 |
| epithelium development | DSP\|NRP1\|COL18A1\|ANXA1\|B4GALT1\|C2CD3\|CSF1\|SEMA3C\|KRT2\|CTSZ\|TNC\|HP\|VEGFC\|FGF2\|BASP1\|KRT14\|CFL1\|DAG1\|ADAM9\|PFN1\|CD44 | 1.17E-04 |
| catabolic process | GPI\|MOXD1\|VCP\|PGLYRP2\|HEXB\|PCSK9\|ENO1\|GNS\|HSP90B1\|PRDX3\|LDHB\|PRDX2\|LDHA\|UCHL1\|NT5E\|PNP\|PRDX1\|ENPP2\|PGLS\|CTSD\|LDLR\|CTSB\|MANBA\|TPI1\|MDH1\|HSPA5\|MDH2\|GAA\|TALDO1\|ADAM10\|PRDX6\|PSMA5\|PSMA6\|PSMA3\|PKM\|DDAH2\|NCL\|PXDN\|ADAM9\|TPP1\|ALDOA\|GAPDH | 1.19E-04 |
| cellular component organization | APP\|SPON2\|NRP1\|COL18A1\|B4GALT1\|C2CD3\|HEXB\|COL12A1\|TNC\|HP\|LAMC2\|LAMC1\|CLU\|CTGF\|ALCAM\|CDH2\|CFL1\|DAG1\|EMILIN1\|ITGAV\|CTSD\|DSP\|POSTN\|HSP90AA1\|ACTN1\|GAA\|ANXA5\|TPM1\|APOA1\|ACTN4\|MIF\|YWHAZ\|DCN\|KRT9\|VCAN\|CCDC80\|LOX\|NCL\|COL6A2\|PXDN\|FSCN1\|CDH13\|ANGPTL4\|PFN1\|ALDOA\|CD44\|VCP\|LRP1\|COL11A1\|THY1\|NID1\|THBS1\|HSP90B1\|PRDX3\|CST3\|LMNA\|PSAP\|SERPINH1\|FLNA\|LDLR\|TXNDC5\|NTNG1\|GSN\|LUM\|FN1\|LAMB1\|SOD2\|TICRR\|COL1A1\|EXT1\|COL3A1\|COL1A2\|COL5A1\|CAPZA1\|KRT14\|COL5A2\|TPP1\|PTX3\|CALR\|TGFBI\|AGRN\|FMOD | 1.37E-04 |
| positive regulation of cell proliferation | NRP1\|COL18A1\|ECM1\|GRN\|B4GALT1\|CSF1\|CD81\|LAMC1\|FGF2\|CLU\|CTGF\|PRDX3\|PNP\|PDGFC\|TIMP1\|CLEC11A\|LIF\|VEGFC\|ADAM10\|LAMB1\|MIF\|TF\|CDH13\|CALR\|TKT | 1.55E-04 |
| aging | GSN\|ATP6AP1\|LRP1\|SERPINF1\|SERPINE1\|MIF\|SOD2\|DCN\|CTGF\|LOXL2\|TF\|CALR\|CTSC | 1.70E-04 |
| aminoglycan metabolic process | EXT1\|EXT2\|ITIH4\|PGLYRP2\|HEXB\|BGN\|XYLT1\|GNS\|DCN | 1.70E-04 |
| glucose metabolic process | GPI\|TPI1\|MDH1\|MDH2\|GAA\|TALDO1\|ENO1\|LDHB\|LDHA\|PKM\|PGLS\|ALDOA\|GAPDH | 1.80E-04 |
| immune response | SPON2\|GPI\|SEMA7A\|PGLYRP2\|C1S\|SEMA3C\|C1R\|CFI\|CLU\|THBS1\|C3\|PRDX2\|IGHG1\|PNP\|PRDX1\|ENPP2\|IGHA1\|CTSC\|KRT1\|LIF\|PRG4\|HLA-A\|MIF\|YWHAZ\|PROCR\|PXDN\|SERPING1\|PTX3\|MASP1\|CFB | 1.80E-04 |
| negative regulation of plasminogen activation | SERPINE2\|SERPINE1\|THBS1 | 1.80E-04 |
| cellular response to hydrogen peroxide | PRDX3\|PRDX2\|TF\|PRDX1\|PXDN\|PRDX6 | 1.80E-04 |
| regulation of cellular protein metabolic process | YWHAE\|APP\|VCP\|HSP90AB1\|SERPINE2\|CSF1\|CD81\|HP\|PCSK9\|HSPB1\|FGF2\|THBS1\|CTGF\|HPX\|ITGAV\|TIMP1\|HSPA5\|IGFBP3\|LIF\|VEGFC\|APOA1\|MIF\|PSMA5\|PSMA6\|PSMA3\|ADAM9\|CALR\|CD44 | 1.95E-04 |
| muscle organ development | APP\|TAGLN\|LAMA2\|SEMA3C\|GAA\|TPM1\|LIF\|TNC\|AEBP1\|DCN\|MYL6\|RCN1\|LMNA\|COL6A3\|AGRN\|CTSB | 1.95E-04 |
| nicotinamide nucleotide metabolic process | LDHB\|PNP\|TPI1\|MDH1\|MDH2\|TALDO1\|PGLS | 2.00E-04 |
| regulation of angiogenesis | C3\|TF\|ECM1\|SRPX2\|SERPINF1\|SERPINE1\|KRT1\|ANGPTL4\|FGF2\|THBS1 | 2.04E-04 |
| locomotion | YWHAE\|NRP1\|B4GALT1\|SEMA3C\|CD81\|KRT2\|FN1\|PLAUR\|VEGFC\|APOA1\|MIF\|LAMC1\|FGF2\|THBS1\|CTGF\|VCAN\|SRPX2\|CDH2\|COL5A1\|CFL1\|ENPP2\|CDH13\|CD44\|CTHRC1 | 2.04E-04 |
| cellular response to oxidative stress | PRDX3\|PRDX2\|TF\|PRDX1\|PXDN\|TPM1\|SOD2\|PRDX6 | 2.10E-04 |
| positive regulation of cell migration | COL18A1\|TF\|SRPX2\|CSF1\|SERPINE1\|VEGFC\|ADAM10\|ADAM9\|CDH13\|LAMB1\|FGF2\|THBS1 | 2.21E-04 |
| multicellular organismal metabolic process | COL1A1\|COL3A1\|COL5A1\|MMP1\|MMP2\|MMP3\|SERPINH1 | 2.30E-04 |
| regulation of cell-substrate adhesion | COL1A1\|LGALS1\|CCDC80\|CSF1\|CDH13\|EMILIN1\|NID1\|THBS1 | 2.35E-04 |
| tissue morphogenesis | NRP1\|C2CD3\|CSF1\|SEMA3C\|GAA\|CTSZ\|TPM1\|TNC\|HP\|VEGFC\|FGF2\|COL1A1\|EXT2\|COL1A2\|CFL1\|DAG1\|PFN1\|CD44 | 2.35E-04 |
| proteolysis | VCP\|C1S\|C1R\|CFI\|CTSZ\|HP\|HTRA1\|PCSK9\|AEBP1\|HSP90B1\|ADAMTS4\|UCHL1\|CTSL\|ANPEP\|CTSD\|CTSC\|CTSB\|HSPA5\|MMP1\|MMP2\|MMP3\|ADAM10\|PSMA5\|ADAM19\|PSMA6\|PSMA3\|BMP1\|QPCT\|ADAM9\|CPE\|TPP1\|MASP1\|CFB | 2.35E-04 |
| regulation of phosphorus metabolic process | YWHAE\|APP\|CSF1\|LRP1\|CD81\|HP\|THY1\|FGF2\|THBS1\|CTGF\|HSP90B1\|PRDX3\|HPX\|PRDX2\|UCHL1\|SRPX2\|YWHAG\|HSPA5\|IGFBP3\|LIF\|VEGFC\|APOA1\|MIF\|INHBA\|RCN1\|ADAM9\|CD44 | 2.87E-04 |
| regulation of phosphate metabolic process | YWHAE\|APP\|CSF1\|LRP1\|CD81\|HP\|THY1\|FGF2\|THBS1\|CTGF\|HSP90B1\|PRDX3\|HPX\|PRDX2\|UCHL1\|SRPX2\|YWHAG\|HSPA5\|IGFBP3\|LIF\|VEGFC\|APOA1\|MIF\|INHBA\|RCN1\|ADAM9\|CD44 | 2.87E-04 |
| regulation of nervous system development | ACTR3\|NRP1\|SERPINE2\|SERPINF1\|XYLT1\|VEGFC\|THY1\|FGF2\|LGALS1\|CDH2\|DPYSL2\|ARHGDIA\|TIMP2\|CALR\|AGRN\|YWHAG | 2.95E-04 |
| pyridine nucleotide metabolic process | LDHB\|PNP\|TPI1\|MDH1\|MDH2\|TALDO1\|PGLS | 3.08E-04 |
| hydrogen peroxide catabolic process | PRDX3\|PRDX2\|PRDX1\|PXDN\|PRDX6 | 3.17E-04 |
| metabolic process | APP\|PGLYRP2\|CD81\|HP\|LOXL3\|XYLT1\|PLOD3\|LOXL4\|PLOD2\|ENO1\|PARK7\|PLOD1\|FGF2\|CLU\|LOXL1\|CTGF\|LOXL2\|EEF1B2\|PNP\|NAGLU\|ANPEP\|DPYSL2\|DPYSL3\|CFL1\|ENPP2\|PTGDS\|RPS12\|IGFBP4\|IGFBP3\|KRT1\|TALDO1\|ADAM10\|RPSA\|MIF\|ADAM19\|MINPP1\|LOX\|NCL\|ERP29\|SERPING1\|ADAM9\|FKBP9\|MASP1\|PPIB\|PPIA\|CFB\|PPIC\|VCP\|CFI\|HTRA1\|AEBP1\|NID1\|GNS\|NID2\|HSP90B1\|C3\|PRDX3\|HPX\|LDHB\|PRDX2\|LDHA\|PRDX1\|NEU1\|PSAP\|SERPINH1\|IGHA1\|HSPA5\|RARRES2\|FN1\|GGH\|INHBA\|PRDX6\|COL1A1\|EXT1\|EXT2\|TF\|HNRNPK\|BMP1\|COL5A1\|PTK7\|CPE\|TPP1\|CALR\|GPI\|FKBP10\|MOXD1\|HSP90AB1\|B4GALT1\|C2CD3\|RPLP1\|HEXB\|HEXA\|CTSZ\|PCSK9\|ADAMTS4\|UCHL1\|CTSL\|RPLP2\|QSOX1\|MAN1A1\|PGLS\|CTSD\|CTSC\|CTSB\|DSP\|MANBA\|HSP90AA1\|ANXA1\|TPI1\|ATP6AP1\|MMP1\|GAA\|MMP2\|ATP6AP2\|MMP3\|BGN\|PLAUR\|APOA1\|REXO2\|DCN\|PSMA5\|PSMA6\|PSMA3\|PKM\|EEF1D\|DDAH2\|PXDN\|TKT\|ALDOA\|PTMA\|GAPDH\|ITIH4\|C1S\|LRP1\|C1R\|GSTP1\|SLC3A2\|THBS1\|NT5E\|GANAB\|FLNA\|LDLR\|MDH1\|MDH2\|TXNL1\|SOD2\|SOD3\|TICRR\|COL3A1\|AXL\|PSAT1\|QPCT\|HNRNPA2B1\|P4HB\|AGRN | 3.55E-04 |
| regulation of cell differentiation | NRP1\|B4GALT1\|SERPINE2\|CSF1\|XYLT1\|THY1\|FGF2\|CLU\|FSTL3\|PRDX2\|PNP\|LGALS1\|CDH2\|DPYSL2\|ARHGDIA\|TIMP2\|ITGAV\|YWHAG\|ACTR3\|SERPINF1\|IGFBP3\|LIF\|VEGFC\|INHBA\|SOD2\|CALR\|AGRN | 3.83E-04 |
| complement activation, classical pathway | C3\|C1S\|C1R\|CFI\|SERPING1\|CLU | 3.87E-04 |
| cell-substrate junction assembly | ACTN1\|KRT14\|HP\|LAMC2\|THY1\|LAMC1 | 3.87E-04 |
| positive regulation of locomotion | COL18A1\|TF\|SRPX2\|CSF1\|SERPINE1\|VEGFC\|ADAM10\|ADAM9\|CDH13\|LAMB1\|FGF2\|THBS1 | 3.97E-04 |
| activation of immune response | C3\|C1S\|C1R\|KRT1\|CFI\|SERPING1\|THY1\|MASP1\|CLU\|CFB | 4.32E-04 |
| hexose metabolic process | GPI\|TPI1\|B4GALT1\|MDH1\|MDH2\|GAA\|TALDO1\|ENO1\|LDHB\|LDHA\|PKM\|PGLS\|ALDOA\|GAPDH | 4.32E-04 |
| muscle structure development | APP\|TAGLN\|LAMA2\|SEMA3C\|GAA\|TPM1\|LIF\|TNC\|AEBP1\|DCN\|MYL6\|RCN1\|LGALS1\|LMNA\|COL6A3\|AGRN\|CTSB | 4.79E-04 |
| regulation of cell growth | NRP1\|IGFBP4\|IGFBP3\|HTRA1\|ADAM10\|ENO1\|INHBA\|FGF2\|CTGF\|WISP2\|CDH13\|IGFBP7\|IGFBP6\|TKT\|CD44 | 4.79E-04 |
| negative regulation of protein metabolic process | YWHAE\|HSP90AB1\|SERPINE2\|IGFBP3\|SERPINE1\|THBS1\|PSMA5\|PSMA6\|PSMA3\|SERPING1\|FLNA\|ITGAV\|CALR\|TIMP1\|MASP1 | 4.79E-04 |
| positive regulation of immune system process | C1S\|C1R\|SERPINE1\|KRT1\|CFI\|VEGFC\|ADAM10\|MIF\|THY1\|CLU\|THBS1\|C3\|HPX\|PNP\|SERPING1\|MASP1\|CFB | 4.95E-04 |
| humoral immune response mediated by circulating immunoglobulin | C3\|C1S\|C1R\|CFI\|SERPING1\|CLU | 5.48E-04 |
| cell development | APP\|SPON2\|NRP1\|COL18A1\|B4GALT1\|SEMA3C\|TNC\|THY1\|LAMC1\|CLU\|ALCAM\|LMNA\|CFL1\|PSAP\|TIMP1\|NTNG1\|GSN\|TPM1\|FN1\|LIF\|LAMB1\|SOD2\|EXT1\|VCAN\|RCN1\|NCL\|PTX3\|AGRN\|CD44 | 5.49E-04 |
| monosaccharide metabolic process | GPI\|TPI1\|B4GALT1\|MDH1\|MDH2\|HEXB\|GAA\|TALDO1\|ENO1\|LDHB\|LDHA\|PKM\|PGLS\|ALDOA\|GAPDH | 5.50E-04 |
| positive regulation of response to stimulus | C1S\|C1R\|SERPINE1\|KRT1\|CFI\|VEGFC\|ADAM10\|THY1\|CLU\|THBS1\|C3\|HPX\|TF\|SERPING1\|CDH13\|MASP1\|CFB | 5.52E-04 |
| innate immune response | SPON2\|PGLYRP2\|C1S\|C1R\|KRT1\|CFI\|MIF\|CLU\|C3\|PRDX1\|SERPING1\|MASP1\|CFB | 5.52E-04 |
| regulation of plasminogen activation | SERPINE2\|SERPINE1\|THBS1 | 5.52E-04 |
| striated muscle tissue development | APP\|MYL6\|RCN1\|SEMA3C\|GAA\|LMNA\|TPM1\|TNC\|AGRN\|DCN\|CTSB | 5.64E-04 |
| regulation of catalytic activity | APP\|ECM1\|VCP\|CSF1\|LRP1\|CD81\|SERPINE1\|GDI2\|TNC\|HP\|PCSK9\|THY1\|FGF2\|THBS1\|CTGF\|HSP90B1\|PRDX3\|CST3\|PRDX2\|UCHL1\|PSAP\|FLNA\|YWHAG\|CTSB\|HSPA5\|TPM2\|TPM1\|APOA1\|SOD2\|PSMA5\|PSMA6\|TF\|PSMA3\|RCN1\|ADAM9\|TPP1\|ANGPTL4 | 5.64E-04 |
| regulation of response to external stimulus | SERPINE2\|SERPINF1\|SERPINE1\|KRT1\|XYLT1\|VEGFC\|ADAM10\|THBS1\|C3\|TF\|NT5E\|SERPING1\|CDH13\|MASP1 | 5.64E-04 |
| positive regulation of cell-substrate adhesion | CCDC80\|CSF1\|CDH13\|EMILIN1\|NID1\|THBS1 | 6.25E-04 |
| regulation of peptidase activity | CST3\|TF\|ECM1\|VCP\|HSPA5\|SERPINE1\|PSAP\|HP\|THBS1\|CTGF | 7.18E-04 |
| oxygen and reactive oxygen species metabolic process | PRDX3\|PRDX2\|PRDX1\|PXDN\|PARK7\|SOD2\|PRDX6\|SOD3 | 7.67E-04 |
| multi-organism process | YWHAE\|APP\|GRN\|PGLYRP2\|CD81\|HEXB\|SERPINE1\|HSPB1\|FBLN1\|ENO1\|CLU\|PRDX3\|HPX\|PRDX2\|ANPEP\|CFL1\|IGFBP7\|ITGAV\|LDLR\|DCD\|MMP1\|LIF\|HLA-A\|DCN\|TF\|PSMA3\|HNRNPK\|PAPPA\|ALB\|ZYX\|PTX3\|VIM\|PPIA | 8.46E-04 |
| positive regulation of protein metabolic process | VCP\|CSF1\|HSPA5\|CD81\|LIF\|HP\|VEGFC\|MIF\|FGF2\|THBS1\|CTGF\|C3\|PSMA5\|HPX\|PSMA6\|PSMA3\|ADAM9\|CD44 | 8.62E-04 |
| negative regulation of cell migration | IGFBP3\|SERPINE1\|TPM1\|DAG1\|HP\|THY1\|FGF2\|THBS1 | 9.40E-04 |
| negative regulation of cellular component organization | NRP1\|GSN\|HP\|XYLT1\|APOA1\|THY1\|CLU\|THBS1\|CST3\|LGALS1\|CAPZA1\|ARHGDIA\|TPP1 | 1.01E-03 |
| muscle tissue development | APP\|MYL6\|RCN1\|SEMA3C\|GAA\|LMNA\|TPM1\|TNC\|AGRN\|DCN\|CTSB | 1.03E-03 |
| oxidoreduction coenzyme metabolic process | LDHB\|PNP\|TPI1\|MDH1\|MDH2\|TALDO1\|PGLS | 1.09E-03 |
| macromolecule metabolic process | APP\|PGLYRP2\|CD81\|HP\|XYLT1\|PLOD3\|PLOD2\|PLOD1\|FGF2\|CLU\|LOXL1\|CTGF\|LOXL2\|EEF1B2\|ANPEP\|CFL1\|RPS12\|IGFBP4\|IGFBP3\|KRT1\|ADAM10\|RPSA\|ADAM19\|LOX\|NCL\|ERP29\|SERPING1\|ADAM9\|FKBP9\|MASP1\|PPIB\|PPIA\|CFB\|PPIC\|VCP\|CFI\|HTRA1\|AEBP1\|NID1\|GNS\|NID2\|HSP90B1\|C3\|HPX\|PRDX2\|SERPINH1\|IGHA1\|HSPA5\|FN1\|INHBA\|COL1A1\|EXT1\|EXT2\|TF\|HNRNPK\|BMP1\|COL5A1\|PTK7\|CPE\|TPP1\|CALR\|FKBP10\|HSP90AB1\|B4GALT1\|C2CD3\|RPLP1\|HEXB\|CTSZ\|PCSK9\|ADAMTS4\|UCHL1\|CTSL\|RPLP2\|QSOX1\|CTSD\|CTSC\|CTSB\|DSP\|MANBA\|HSP90AA1\|ANXA1\|MMP1\|GAA\|MMP2\|ATP6AP2\|MMP3\|BGN\|PLAUR\|APOA1\|DCN\|PSMA5\|PSMA6\|PSMA3\|EEF1D\|TKT\|PTMA\|ITIH4\|C1S\|LRP1\|C1R\|THBS1\|NT5E\|FLNA\|LDLR\|TICRR\|COL3A1\|AXL\|QPCT\|HNRNPA2B1\|P4HB\|AGRN | 1.11E-03 |
| regulation of humoral immune response | C3\|HPX\|SERPING1\|MASP1 | 1.11E-03 |
| regulation of hydrolase activity | ECM1\|VCP\|HSPA5\|LRP1\|TPM2\|SERPINE1\|GDI2\|TPM1\|TNC\|HP\|APOA1\|THY1\|THBS1\|CTGF\|HSP90B1\|CST3\|TF\|RCN1\|PSAP\|ANGPTL4 | 1.20E-03 |
| monocyte activation | CSF1\|ADAM10\|ADAM9 | 1.22E-03 |
| regulation of complement activation | C3\|SERPING1\|MASP1 | 1.22E-03 |
| primary metabolic process | APP\|PGLYRP2\|CD81\|HP\|XYLT1\|PLOD3\|PLOD2\|ENO1\|PLOD1\|FGF2\|CLU\|LOXL1\|CTGF\|LOXL2\|EEF1B2\|PNP\|ANPEP\|DPYSL2\|DPYSL3\|CFL1\|ENPP2\|PTGDS\|RPS12\|IGFBP4\|IGFBP3\|KRT1\|TALDO1\|ADAM10\|RPSA\|MIF\|ADAM19\|LOX\|NCL\|ERP29\|SERPING1\|ADAM9\|FKBP9\|MASP1\|PPIB\|PPIA\|CFB\|PPIC\|VCP\|CFI\|HTRA1\|AEBP1\|NID1\|GNS\|NID2\|HSP90B1\|C3\|HPX\|LDHB\|PRDX2\|LDHA\|PSAP\|SERPINH1\|IGHA1\|HSPA5\|RARRES2\|FN1\|GGH\|INHBA\|PRDX6\|EXT1\|EXT2\|TF\|HNRNPK\|BMP1\|PTK7\|CPE\|TPP1\|CALR\|GPI\|FKBP10\|MOXD1\|HSP90AB1\|B4GALT1\|C2CD3\|RPLP1\|HEXB\|HEXA\|CTSZ\|PCSK9\|ADAMTS4\|UCHL1\|CTSL\|RPLP2\|QSOX1\|PGLS\|CTSD\|CTSC\|CTSB\|DSP\|MANBA\|HSP90AA1\|ANXA1\|TPI1\|ATP6AP1\|MMP1\|GAA\|MMP2\|ATP6AP2\|MMP3\|BGN\|PLAUR\|APOA1\|REXO2\|DCN\|PSMA5\|PSMA6\|PSMA3\|PKM\|EEF1D\|DDAH2\|TKT\|ALDOA\|PTMA\|GAPDH\|ITIH4\|C1S\|LRP1\|C1R\|SLC3A2\|THBS1\|NT5E\|GANAB\|LDLR\|MDH1\|MDH2\|SOD2\|TICRR\|COL3A1\|AXL\|PSAT1\|QPCT\|HNRNPA2B1\|P4HB | 1.29E-03 |
| positive regulation of cellular protein metabolic process | VCP\|CSF1\|HSPA5\|CD81\|LIF\|HP\|VEGFC\|MIF\|FGF2\|THBS1\|CTGF\|PSMA5\|HPX\|PSMA6\|PSMA3\|ADAM9\|CD44 | 1.30E-03 |
| regulation of neurogenesis | ACTR3\|NRP1\|SERPINE2\|SERPINF1\|XYLT1\|VEGFC\|THY1\|LGALS1\|CDH2\|DPYSL2\|ARHGDIA\|TIMP2\|CALR\|YWHAG | 1.36E-03 |
| regulation of molecular function | APP\|ECM1\|VCP\|CSF1\|LRP1\|CD81\|SERPINE1\|GDI2\|TNC\|HP\|PCSK9\|THY1\|PARK7\|FGF2\|THBS1\|CTGF\|HSP90B1\|PRDX3\|CST3\|PRDX2\|UCHL1\|PSAP\|FLNA\|YWHAG\|CTSB\|HSPA5\|TPM2\|TPM1\|APOA1\|ACTN4\|MIF\|SOD2\|PSMA5\|PSMA6\|TF\|PSMA3\|RCN1\|ADAM9\|TPP1\|ANGPTL4 | 1.39E-03 |
| small molecule catabolic process | GPI\|MOXD1\|TPI1\|MDH1\|MDH2\|TALDO1\|ENO1\|LDHB\|LDHA\|NT5E\|PNP\|PKM\|DDAH2\|PGLS\|ALDOA\|GAPDH | 1.40E-03 |
| negative regulation of locomotion | IGFBP3\|SERPINE1\|TPM1\|DAG1\|HP\|THY1\|FGF2\|THBS1 | 1.46E-03 |
| positive regulation of blood vessel endothelial cell migration | SRPX2\|VEGFC\|FGF2\|THBS1 | 1.50E-03 |
| polysaccharide metabolic process | EXT1\|EXT2\|ITIH4\|PGLYRP2\|HEXB\|GAA\|BGN\|XYLT1\|GNS\|DCN | 1.51E-03 |
| positive regulation of endocytosis | C3\|SERPINE1\|PCSK9\|PTX3\|ACTN4\|CALR | 1.51E-03 |
| neuron development | NTNG1\|APP\|SPON2\|NRP1\|LIF\|LAMB1\|THY1\|LAMC1\|SOD2\|CLU\|EXT1\|VCAN\|ALCAM\|NCL\|PSAP\|PTX3\|AGRN\|CD44 | 1.60E-03 |
| regulation of transport | ANXA1\|B4GALT1\|LRP1\|SERPINE1\|LIF\|TNC\|PCSK9\|VEGFC\|APOA1\|ACTN4\|MIF\|THY1\|INHBA\|FGF2\|THBS1\|C3\|DPYSL2\|PRDX1\|FLNA\|ADAM9\|CDH13\|ITGAV\|PTX3\|CALR | 1.70E-03 |
| positive regulation of nitric oxide biosynthetic process | HSP90AA1\|HSP90AB1\|HBB\|PTX3\|SOD2 | 1.78E-03 |
| regulation of growth | APP\|NRP1\|CSF1\|IGFBP4\|IGFBP3\|HTRA1\|ADAM10\|ENO1\|INHBA\|FGF2\|CTGF\|WISP2\|CDH13\|IGFBP7\|IGFBP6\|TKT\|GAS6\|AGRN\|CD44 | 1.84E-03 |
| negative regulation of hydrolase activity | CST3\|ECM1\|HSPA5\|SERPINE1\|APOA1\|ANGPTL4\|THBS1 | 2.25E-03 |
| positive regulation of chemotaxis | TF\|SERPINE1\|VEGFC\|ADAM10\|CDH13\|THBS1 | 2.26E-03 |
| regulation of vesicle-mediated transport | C3\|ANXA1\|B4GALT1\|SERPINE1\|PCSK9\|CDH13\|PTX3\|ACTN4\|CALR\|FGF2 | 2.38E-03 |
| cellular response to chemical stimulus | VCP\|HSPA5\|SERPINE1\|TPM1\|HP\|PCSK9\|PLOD3\|MIF\|SOD2\|PRDX6\|HSP90B1\|PRDX3\|COL1A1\|PRDX2\|TF\|LGALS1\|PRDX1\|PXDN\|CDH13\|IGFBP7 | 2.41E-03 |
| positive regulation of immune response | C3\|HPX\|C1S\|C1R\|KRT1\|CFI\|SERPING1\|THY1\|MASP1\|CLU\|CFB | 2.56E-03 |
| cellular homeostasis | PDIA3\|APP\|HEXB\|GAA\|TXNL1\|HP\|PARK7\|SOD2\|PRDX6\|PDIA4\|HSP90B1\|PRDX3\|HPX\|PRDX2\|TF\|PRDX1\|NCL\|QSOX1\|CALR\|P4HB\|ALDOA\|TXNDC5 | 2.69E-03 |
| homeostatic process | APP\|CSF1\|HEXB\|SERPINE1\|HP\|PCSK9\|PARK7\|HSP90B1\|PRDX3\|HPX\|PRDX2\|PRDX1\|QSOX1\|TIMP1\|LDLR\|TXNDC5\|PDIA3\|GAA\|TXNL1\|APOA1\|INHBA\|SOD2\|PRDX6\|PDIA4\|TF\|NCL\|TPP1\|CALR\|ANGPTL4\|P4HB\|ALDOA | 2.77E-03 |
| regulation of coagulation | TF\|SERPINE2\|SERPINE1\|KRT1\|ANXA5\|THBS1 | 2.87E-03 |
| negative regulation of peptidase activity | CST3\|ECM1\|HSPA5\|SERPINE1\|THBS1 | 2.97E-03 |
| tube morphogenesis | NRP1\|B4GALT1\|C2CD3\|CSF1\|CTSZ\|HP\|HBB\|SOD2\|FGF2\|CFL1\|DAG1\|PFN1\|CD44 | 3.06E-03 |
| negative regulation of response to stimulus | PRDX2\|NT5E\|COL3A1\|SERPINF1\|SERPINE1\|XYLT1\|SERPING1\|MIF\|MASP1\|CD44 | 3.18E-03 |
| positive regulation of epithelial cell proliferation | GRN\|B4GALT1\|VEGFC\|LAMB1\|LAMC1\|FGF2 | 3.20E-03 |
| negative regulation of developmental process | NRP1\|ECM1\|SERPINF1\|SERPINE1\|LIF\|XYLT1\|THY1\|INHBA\|SOD2\|THBS1\|FSTL3\|CST3\|PRDX2\|ARHGDIA\|ITGAV\|CALR | 3.54E-03 |
| leukocyte mediated immunity | C3\|C1S\|C1R\|PRDX1\|CFI\|SERPING1\|CLU\|YWHAZ | 3.54E-03 |
| lymphocyte mediated immunity | C3\|C1S\|C1R\|PRDX1\|CFI\|SERPING1\|CLU | 3.54E-03 |
| fibril organization | CST3\|COL3A1\|COL5A1 | 3.55E-03 |
| morphogenesis of an epithelium | NRP1\|C2CD3\|CSF1\|SEMA3C\|CTSZ\|TNC\|HP\|VEGFC\|FGF2\|CFL1\|DAG1\|PFN1\|CD44 | 3.75E-03 |
| odontogenesis | COL1A1\|DSP\|COL1A2\|CSF1\|LAMB1\|INHBA\|FMOD | 3.85E-03 |
| skeletal muscle tissue development | APP\|MYL6\|RCN1\|TNC\|AGRN\|DCN\|CTSB | 4.22E-03 |
| regulation of biological process | APP\|SPARC\|SERPINE2\|CSF1\|CD81\|SERPINE1\|TNC\|HP\|XYLT1\|ENO1\|PARK7\|FGF2\|CLU\|CTGF\|COMP\|ALCAM\|PNP\|LGALS1\|CDH2\|BASP1\|DPYSL2\|ARHGDIA\|DPYSL3\|CFL1\|DAG1\|ENPP2\|PTGDS\|PDIA3\|IGFBP4\|ACTN1\|SERPINF1\|IGFBP3\|TPM2\|CLEC11A\|KRT1\|TPM1\|ADAM10\|ACTN4\|MIF\|DKK3\|PDIA4\|CCDC80\|SERPING1\|ADAM9\|MASP1\|PFN1\|PPIA\|CFB\|PPIC\|VCP\|CFI\|HTRA1\|THY1\|AEBP1\|NID1\|HSP90B1\|C3\|PRDX3\|HPX\|PRDX2\|PRDX1\|PDGFC\|PSAP\|IGFBP7\|IGFBP6\|TXNDC5\|HSPA5\|FN1\|LIF\|INHBA\|PRDX6\|COL1A1\|EXT1\|EXT2\|TF\|COL1A2\|HNRNPK\|TFCP2\|BMP1\|COL5A1\|PTK7\|ALB\|TPP1\|CALR\|TGFBI\|YWHAE\|LGALS3BP\|NRP1\|COL18A1\|ECM1\|HSP90AB1\|B4GALT1\|C2CD3\|HEXB\|GDI2\|HBB\|PCSK9\|HSPB1\|LAMC1\|WISP2\|UCHL1\|YWHAQ\|ANXA6\|TIMP2\|QSOX1\|EMILIN1\|ITGAV\|TIMP1\|YWHAG\|CTSB\|ACTR3\|HSP90AA1\|ANXA1\|GAA\|ANXA5\|ATP6AP2\|PLAUR\|APOA1\|YWHAZ\|PSMA5\|RCN3\|PSMA6\|PSMA3\|RCN1\|EEF1D\|DDAH2\|CDH13\|ANGPTL4\|TKT\|ALDOA\|GAS6\|CD44\|GRN\|COL15A1\|LAMA2\|C1S\|LRP1\|C1R\|GSTP1\|LAMA4\|THBS1\|FSTL3\|CST3\|NT5E\|SRPX2\|FLNA\|SPOCK1\|GSN\|TXNL1\|VEGFC\|LAMB1\|SOD2\|TICRR\|MFAP4\|COL3A1\|AXL\|CAPZA1\|ZYX\|PTX3\|P4HB\|AGRN | 4.35E-03 |
| positive regulation of behavior | TF\|SERPINE1\|VEGFC\|ADAM10\|CDH13\|THBS1 | 4.42E-03 |
| regulation of phosphorylation | APP\|CSF1\|HSPA5\|LRP1\|CD81\|IGFBP3\|LIF\|VEGFC\|APOA1\|MIF\|THY1\|INHBA\|FGF2\|THBS1\|CTGF\|PRDX3\|HPX\|PRDX2\|UCHL1\|SRPX2\|ADAM9\|CD44\|YWHAG | 4.42E-03 |
| regulation of proteolysis | VCP\|HSP90AB1\|SERPINE2\|C2CD3\|PLAUR\|ADAM9\|TIMP1 | 4.52E-03 |
| regulation of nitric oxide biosynthetic process | HSP90AA1\|HSP90AB1\|HBB\|PTX3\|SOD2 | 4.52E-03 |
| tube development | NRP1\|B4GALT1\|C2CD3\|CSF1\|CTSZ\|LIF\|HP\|HBB\|SOD2\|FGF2\|CTGF\|LOX\|CFL1\|DAG1\|PFN1\|CD44 | 4.52E-03 |
| positive regulation of phosphate metabolic process | HPX\|CD81\|LIF\|HP\|VEGFC\|MIF\|FGF2\|THBS1\|CD44\|CTGF | 4.83E-03 |
| positive regulation of phosphorus metabolic process | HPX\|CD81\|LIF\|HP\|VEGFC\|MIF\|FGF2\|THBS1\|CD44\|CTGF | 4.83E-03 |
| skeletal muscle organ development | APP\|MYL6\|RCN1\|TNC\|AGRN\|DCN\|CTSB | 4.87E-03 |
| positive regulation of transport | ANXA1\|LRP1\|SERPINE1\|PCSK9\|VEGFC\|ACTN4\|MIF\|THY1\|INHBA\|C3\|DPYSL2\|FLNA\|ADAM9\|PTX3\|CALR | 4.89E-03 |
| negative regulation of catalytic activity | ECM1\|HSPA5\|SERPINE1\|PCSK9\|APOA1\|THY1\|THBS1\|PRDX3\|PSMA5\|CST3\|PSMA6\|UCHL1\|PSMA3\|FLNA\|ANGPTL4\|YWHAG | 4.93E-03 |
| biological regulation | APP\|SPARC\|SERPINE2\|CSF1\|CD81\|SERPINE1\|TNC\|HP\|XYLT1\|ENO1\|PARK7\|FGF2\|CLU\|CTGF\|COMP\|EFEMP2\|ALCAM\|PNP\|LGALS1\|CDH2\|BASP1\|DPYSL2\|ARHGDIA\|DPYSL3\|CFL1\|DAG1\|ENPP2\|PTGDS\|PDIA3\|IGFBP4\|ACTN1\|SERPINF1\|IGFBP3\|TPM2\|CLEC11A\|KRT1\|TPM1\|ADAM10\|ACTN4\|MIF\|DKK3\|PDIA4\|PROCR\|CCDC80\|NCL\|SERPING1\|ADAM9\|MASP1\|PFN1\|PPIA\|CFB\|PPIC\|VCP\|CFI\|HTRA1\|THY1\|AEBP1\|NID1\|HSP90B1\|C3\|PRDX3\|HPX\|PRDX2\|PRDX1\|PDGFC\|PSAP\|IGFBP7\|IGFBP6\|TXNDC5\|HSPA5\|FN1\|LIF\|INHBA\|PRDX6\|COL1A1\|EXT1\|EXT2\|TF\|COL1A2\|HNRNPK\|TFCP2\|BMP1\|COL5A1\|PTK7\|ALB\|CPE\|TPP1\|CALR\|TGFBI\|YWHAE\|LGALS3BP\|GPI\|NRP1\|COL18A1\|ECM1\|HSP90AB1\|B4GALT1\|C2CD3\|HEXB\|GDI2\|HBB\|PCSK9\|HSPB1\|LAMC1\|WISP2\|UCHL1\|YWHAQ\|ANXA6\|TIMP2\|QSOX1\|EMILIN1\|ITGAV\|TIMP1\|YWHAG\|CTSB\|ACTR3\|HSP90AA1\|ANXA1\|GAA\|ANXA5\|ATP6AP2\|PLAUR\|APOA1\|YWHAZ\|PSMA5\|RCN3\|PSMA6\|PSMA3\|RCN1\|EEF1D\|DDAH2\|CDH13\|ANGPTL4\|TKT\|ALDOA\|GAS6\|CD44\|GRN\|COL15A1\|LAMA2\|C1S\|LRP1\|C1R\|GSTP1\|LAMA4\|SLC3A2\|THBS1\|FSTL3\|CST3\|NT5E\|SRPX2\|FLNA\|SPOCK1\|LDLR\|GSN\|TXNL1\|VEGFC\|LAMB1\|SOD2\|TICRR\|MFAP4\|COL3A1\|AXL\|CAPZA1\|ZYX\|PTX3\|P4HB\|AGRN | 5.70E-03 |
| immunoglobulin mediated immune response | C3\|C1S\|C1R\|CFI\|SERPING1\|CLU | 5.77E-03 |
| regulation of chemotaxis | TF\|SERPINE1\|VEGFC\|ADAM10\|CDH13\|THBS1 | 6.38E-03 |
| B cell mediated immunity | C3\|C1S\|C1R\|CFI\|SERPING1\|CLU | 6.38E-03 |
| actin filament-based process | GSN\|TPM1\|HP\|ACTN4\|MIF\|HSP90B1\|MYL6\|CAPZA1\|CFL1\|FSCN1\|FLNA\|CALR\|PFN1\|ALDOA | 6.67E-03 |
| response to protein stimulus | ACTR3\|VCP\|HSP90AA1\|HSP90AB1\|HSPA5\|SERPINH1\|HSPB1\|CLU\|CTSB | 7.01E-03 |
| negative regulation of molecular function | ECM1\|HSPA5\|SERPINE1\|PCSK9\|APOA1\|THY1\|PARK7\|THBS1\|PRDX3\|PSMA5\|CST3\|PRDX2\|PSMA6\|UCHL1\|PSMA3\|FLNA\|ANGPTL4\|YWHAG | 7.01E-03 |
| regulation of endocytosis | C3\|SERPINE1\|PCSK9\|CDH13\|PTX3\|ACTN4\|CALR | 7.01E-03 |
| cell junction organization | DSP\|ACTN1\|KRT14\|HP\|LAMC2\|THY1\|LAMC1 | 7.01E-03 |
| neuron maturation | APP\|NCL\|PSAP\|AGRN | 7.01E-03 |
| urogenital system development | BASP1\|SERPINF1\|TNC\|PCSK9\|TPP1\|MIF\|NID1\|CD44\|DCN\|FBN1 | 7.01E-03 |
| pentose-phosphate shunt | TPI1\|TALDO1\|PGLS | 7.12E-03 |
| apoptotic cell clearance | LRP1\|ITGAV\|THBS1 | 7.12E-03 |
| regulation of neuron differentiation | ACTR3\|NRP1\|LGALS1\|CDH2\|DPYSL2\|ARHGDIA\|TIMP2\|XYLT1\|THY1\|CALR\|YWHAG | 7.61E-03 |
| protein folding | FKBP10\|HSP90AA1\|HSP90AB1\|ERP29\|QSOX1\|CALR\|FKBP9\|PPIB\|PPIA\|PPIC\|HSP90B1 | 7.61E-03 |
| cellular carbohydrate metabolic process | GPI\|TPI1\|B4GALT1\|MDH1\|MDH2\|HEXB\|GAA\|TALDO1\|ENO1\|EXT1\|LDHB\|EXT2\|LDHA\|PKM\|PGLS\|ALDOA\|LDLR\|GAPDH | 7.90E-03 |
| cell junction assembly | ACTN1\|KRT14\|HP\|LAMC2\|THY1\|LAMC1 | 8.21E-03 |
| muscle cell development | APP\|RCN1\|LMNA\|TPM1\|TNC\|AGRN | 9.03E-03 |
| regulation of endopeptidase activity | TF\|VCP\|HSPA5\|SERPINE1\|PSAP\|HP\|THBS1\|CTGF | 9.05E-03 |
| positive regulation of protein modification process | HSPA5\|CD81\|LIF\|HP\|VEGFC\|MIF\|FGF2\|CTGF\|PSMA5\|HPX\|PSMA6\|PSMA3\|CD44 | 9.34E-03 |
| regulation of macrophage differentiation | CSF1\|LIF\|INHBA | 9.60E-03 |
| substrate adhesion-dependent cell spreading | FN1\|LAMB1\|LAMC1 | 9.60E-03 |
| NADPH regeneration | TPI1\|TALDO1\|PGLS | 9.60E-03 |
| regulation of blood vessel endothelial cell migration | SRPX2\|VEGFC\|FGF2\|THBS1 | 9.65E-03 |
| NAD metabolic process | LDHB\|PNP\|MDH1\|MDH2 | 9.65E-03 |
| negative regulation of signaling pathway | HSPA5\|LRP1\|LIF\|HP\|HTRA1\|MIF\|THY1\|THBS1\|DKK3\|FSTL3\|PRDX2\|DAG1\|CALR\|CD44 | 9.65E-03 |
| actin filament organization | GSN\|CFL1\|FSCN1\|FLNA\|ACTN4\|ALDOA\|HSP90B1 | 9.78E-03 |
| regulation of signal transduction | APP\|ECM1\|HSP90AB1\|CSF1\|CD81\|SERPINE1\|HP\|PCSK9\|FGF2\|THBS1\|HPX\|PRDX2\|UCHL1\|LGALS1\|CDH2\|PRDX1\|PSAP\|DAG1\|TIMP2\|FLNA\|YWHAG\|IGFBP3\|ATP6AP2\|LIF\|MIF\|TF\|EEF1D\|CDH13\|CD44 | 1.03E-02 |
| integrin-mediated signaling pathway | COL3A1\|ITGBL1\|ADAM10\|ADAM9\|ITGAV\|CTGF | 1.04E-02 |
| actin cytoskeleton organization | GSN\|TPM1\|HP\|ACTN4\|MIF\|HSP90B1\|CAPZA1\|CFL1\|FSCN1\|FLNA\|CALR\|PFN1\|ALDOA | 1.05E-02 |
| regulation of immune response | C1S\|C1R\|KRT1\|CFI\|APOA1\|THY1\|CLU\|C3\|HPX\|COL3A1\|SERPING1\|MASP1\|CFB | 1.05E-02 |
| epithelial tube morphogenesis | C2CD3\|CSF1\|CTSZ\|CFL1\|DAG1\|HP\|PFN1\|FGF2\|CD44 | 1.08E-02 |
| regulation of signaling pathway | APP\|ECM1\|HSP90AB1\|C2CD3\|CSF1\|LRP1\|CD81\|HP\|HTRA1\|THY1\|PARK7\|FGF2\|THBS1\|FSTL3\|HPX\|PRDX2\|UCHL1\|LGALS1\|CDH2\|PRDX1\|DAG1\|TIMP2\|FLNA\|HSPA5\|ATP6AP2\|LIF\|VEGFC\|MIF\|INHBA\|DKK3\|TF\|EEF1D\|CDH13\|CALR\|CD44 | 1.10E-02 |
| regulation of blood coagulation | TF\|SERPINE2\|SERPINE1\|KRT1\|THBS1 | 1.10E-02 |
| negative regulation of cell projection organization | NRP1\|LGALS1\|ARHGDIA\|XYLT1\|THY1 | 1.10E-02 |
| regulation of signaling process | APP\|ECM1\|HSP90AB1\|CSF1\|CD81\|SERPINE1\|HP\|PCSK9\|FGF2\|THBS1\|HPX\|PRDX2\|UCHL1\|LGALS1\|CDH2\|PRDX1\|PSAP\|DAG1\|TIMP2\|FLNA\|YWHAG\|IGFBP3\|ATP6AP2\|LIF\|MIF\|TF\|EEF1D\|CDH13\|CD44 | 1.10E-02 |
| positive regulation of leukocyte chemotaxis | SERPINE1\|VEGFC\|ADAM10\|THBS1 | 1.10E-02 |
| sprouting angiogenesis | CDH13\|FGF2\|THBS1 | 1.21E-02 |
| hemidesmosome assembly | KRT14\|LAMC2\|LAMC1 | 1.21E-02 |
| alcohol metabolic process | GPI\|MOXD1\|TPI1\|B4GALT1\|MDH1\|MDH2\|HEXB\|GAA\|TALDO1\|PCSK9\|APOA1\|ENO1\|LDHB\|LDHA\|PKM\|PGLS\|ALDOA\|LDLR\|GAPDH | 1.25E-02 |
| Rho protein signal transduction | COL1A2\|ARHGDIA\|CFL1\|APOA1\|CDH13 | 1.33E-02 |
| positive regulation of phosphorylation | HPX\|CD81\|LIF\|VEGFC\|MIF\|FGF2\|THBS1\|CD44\|CTGF | 1.34E-02 |
| response to oxygen levels | MMP2\|PLOD2\|ACTN4\|ANGPTL4\|PLOD1\|SOD2\|THBS1\|SOD3\|CTGF\|HSP90B1 | 1.37E-02 |
| positive regulation of endothelial cell migration | SRPX2\|VEGFC\|FGF2\|THBS1 | 1.44E-02 |
| lysosome organization | HEXB\|GAA\|NCL\|TPP1 | 1.44E-02 |
| cardiac ventricle morphogenesis | SEMA3C\|GAA\|TPM1\|TNC | 1.44E-02 |
| neuron differentiation | NTNG1\|APP\|SPON2\|NRP1\|LIF\|PCSK9\|LAMB1\|THY1\|LAMC1\|SOD2\|CLU\|EXT1\|VCAN\|ALCAM\|NCL\|PSAP\|PTX3\|AGRN\|CD44 | 1.55E-02 |
| hemostasis | GPI\|PROCR\|TF\|COL3A1\|EFEMP2\|ANXA5\|PLAUR\|SERPING1 | 1.58E-02 |
| regulation of epithelial cell proliferation | GRN\|B4GALT1\|SERPINF1\|VEGFC\|LAMB1\|LAMC1\|FGF2 | 1.58E-02 |
| regulation of wound healing | TF\|SERPINE2\|SERPINE1\|KRT1\|THBS1 | 1.60E-02 |
| negative regulation of axonogenesis | NRP1\|ARHGDIA\|XYLT1\|THY1 | 1.64E-02 |
| negative regulation of coagulation | SERPINE2\|SERPINE1\|KRT1\|ANXA5 | 1.64E-02 |
| maintenance of location | PDIA3\|GAA\|ALB\|DAG1\|FLNA\|CALR\|HSP90B1 | 1.66E-02 |
| heart development | NRP1\|COL3A1\|C2CD3\|COL5A1\|SEMA3C\|GAA\|LMNA\|TPM1\|TNC\|HP\|SOD2\|FBN1 | 1.71E-02 |
| regulation of cell communication | APP\|ECM1\|HSP90AB1\|CSF1\|LRP1\|CD81\|SERPINE1\|HP\|HTRA1\|PCSK9\|THY1\|FGF2\|THBS1\|FSTL3\|HPX\|PRDX2\|UCHL1\|LGALS1\|CDH2\|DPYSL2\|PRDX1\|PSAP\|DAG1\|TIMP2\|FLNA\|YWHAG\|HSPA5\|IGFBP3\|ATP6AP2\|LIF\|MIF\|INHBA\|DKK3\|TF\|EEF1D\|CDH13\|CALR\|CD44 | 1.73E-02 |
| proteoglycan metabolic process | EXT1\|EXT2\|BGN\|XYLT1\|DCN | 1.73E-02 |
| vacuole organization | HEXB\|GAA\|NCL\|TPP1\|CTSD | 1.73E-02 |
| positive regulation of cellular component organization | C3\|ACTR3\|ANXA1\|ARHGDIA\|SERPINE1\|CFL1\|TPM1\|PCSK9\|ADAM9\|PTX3\|ACTN4\|CALR | 1.74E-02 |
| heart morphogenesis | C2CD3\|COL5A1\|SEMA3C\|GAA\|TPM1\|TNC\|HP | 1.74E-02 |
| positive regulation of response to external stimulus | C3\|TF\|SERPINE1\|VEGFC\|ADAM10\|CDH13\|THBS1 | 1.74E-02 |
| response to acid | GSN\|CFL1\|MMP3\|CTGF | 1.82E-02 |
| skeletal muscle fiber development | APP\|RCN1\|TNC\|AGRN | 1.82E-02 |
| response to unfolded protein | VCP\|HSP90AA1\|HSP90AB1\|HSPA5\|SERPINH1\|HSPB1 | 1.94E-02 |
| regulation of response to stress | SERPINE2\|SERPINF1\|SERPINE1\|KRT1\|XYLT1\|MIF\|THBS1\|C3\|HPX\|TF\|NT5E\|PRDX1\|SERPING1\|MASP1\|CD44 | 1.95E-02 |
| response to drug | COL18A1\|SEMA3C\|VEGFC\|GGH\|MIF\|PARK7\|SOD2\|YWHAZ\|THBS1\|PNP\|LGALS1\|LOX\|DPYSL2 | 1.95E-02 |
| response to amine stimulus | DPYSL2\|CFL1\|MMP3\|CTSB\|CTGF | 2.05E-02 |
| branching morphogenesis of a tube | NRP1\|B4GALT1\|CSF1\|CTSZ\|DAG1\|FGF2\|CD44 | 2.05E-02 |
| regulation of leukocyte chemotaxis | SERPINE1\|VEGFC\|ADAM10\|THBS1 | 2.05E-02 |
| regulation of intracellular protein kinase cascade | ECM1\|ATP6AP2\|LIF\|MIF\|FGF2\|THBS1\|HPX\|TF\|LGALS1\|EEF1D\|PRDX1\|DAG1\|TIMP2\|FLNA\|CD44 | 2.10E-02 |
| negative regulation of immune system process | PRDX2\|COL3A1\|SERPING1\|THY1\|MASP1\|INHBA\|THBS1 | 2.11E-02 |
| response to endogenous stimulus | SERPINF1\|SERPINE1\|MMP3\|HP\|PCSK9\|PLOD3\|GGH\|THBS1\|CTGF\|COL1A1\|TF\|LOX\|DPYSL2\|CFL1\|DAG1\|ADAM9\|CDH13\|IGFBP7\|LDLR\|CTSB | 2.13E-02 |
| positive regulation of peptidyl-serine phosphorylation | LIF\|MIF\|CD44 | 2.17E-02 |
| positive regulation of blood coagulation | TF\|SERPINE1\|THBS1 | 2.17E-02 |
| regulation of caspase activity | TF\|VCP\|HSPA5\|PSAP\|HP\|THBS1\|CTGF | 2.22E-02 |
| interspecies interaction between organisms | YWHAE\|CD81\|MMP1\|FBLN1\|HLA-A\|HPX\|PSMA3\|HNRNPK\|ANPEP\|ALB\|ZYX\|ITGAV\|VIM\|LDLR\|PPIA | 2.31E-02 |
| striated muscle cell development | APP\|RCN1\|TPM1\|TNC\|AGRN | 2.33E-02 |
| negative regulation of cell communication | HSPA5\|LRP1\|IGFBP3\|LIF\|HP\|HTRA1\|MIF\|THY1\|THBS1\|DKK3\|FSTL3\|PRDX2\|DAG1\|CALR\|CD44 | 2.37E-02 |
| adaptive immune response based on somatic recombination of immune receptors built from immunoglobulin superfamily domains | C3\|C1S\|C1R\|CFI\|SERPING1\|CLU | 2.42E-02 |
| positive regulation of protein amino acid phosphorylation | HPX\|CD81\|LIF\|VEGFC\|MIF\|FGF2\|CD44\|CTGF | 2.53E-02 |
| adaptive immune response | C3\|C1S\|C1R\|CFI\|SERPING1\|CLU | 2.59E-02 |
| cellular component assembly | APP\|VCP\|C2CD3\|HP\|LAMC2\|THY1\|LAMC1\|HSP90B1\|CDH2\|FLNA\|CTSD\|HSP90AA1\|GSN\|ACTN1\|ANXA5\|TPM1\|APOA1\|ACTN4\|MIF\|SOD2\|TICRR\|LOX\|CAPZA1\|KRT14\|FSCN1\|CDH13\|TPP1\|CALR\|ANGPTL4\|AGRN\|FMOD | 2.60E-02 |
| focal adhesion assembly | ACTN1\|HP\|THY1 | 2.60E-02 |
| morphogenesis of a branching structure | NRP1\|B4GALT1\|CSF1\|SEMA3C\|CTSZ\|DAG1\|FGF2\|CD44 | 2.63E-02 |
| regulation of protein modification process | YWHAE\|HSPA5\|CD81\|IGFBP3\|LIF\|HP\|VEGFC\|APOA1\|MIF\|FGF2\|CTGF\|PSMA5\|HPX\|PSMA6\|PSMA3\|CD44 | 2.65E-02 |
| response to hypoxia | MMP2\|PLOD2\|ACTN4\|ANGPTL4\|PLOD1\|SOD2\|THBS1\|SOD3\|HSP90B1 | 2.70E-02 |
| regulation of cell morphogenesis | ACTR3\|NRP1\|CDH2\|ARHGDIA\|FN1\|HP\|XYLT1\|THY1\|ALDOA | 2.70E-02 |
| cellular response to extracellular stimulus | COL1A1\|LDHA\|HSPA5\|AXL\|ALB\|PCSK9\|CTSD | 2.70E-02 |
| protein secretion | PNP\|ERP29\|LTBP2\|PDIA4 | 2.72E-02 |
| neuromuscular process controlling balance | APP\|HEXB\|GAA\|TPP1 | 2.72E-02 |
| positive regulation of leukocyte migration | SERPINE1\|VEGFC\|ADAM10\|THBS1 | 2.72E-02 |
| cardiac ventricle development | SEMA3C\|GAA\|TPM1\|TNC | 2.72E-02 |
| cellular response to external stimulus | COL1A1\|LDHA\|HSPA5\|AXL\|ALB\|PCSK9\|CTSD | 2.82E-02 |
| regulation of actin filament-based process | ACTR3\|GSN\|CAPZA1\|CFL1\|TPM1\|TNC\|HP | 2.97E-02 |
| developmental maturation | APP\|NCL\|MMP2\|PSAP\|TIMP1\|AGRN\|CD44 | 2.97E-02 |
| regulation of dephosphorylation | YWHAE\|RCN1\|HP\|HSP90B1 | 2.98E-02 |
| circulatory system process | RCN1\|COL1A2\|GAA\|TPM1\|ATP6AP2\|TNC\|SERPING1\|HBB\|VEGFC\|SOD2 | 2.98E-02 |
| blood circulation | RCN1\|COL1A2\|GAA\|TPM1\|ATP6AP2\|TNC\|SERPING1\|HBB\|VEGFC\|SOD2 | 2.98E-02 |
| positive regulation of phagocytosis | C3\|PTX3\|CALR | 2.98E-02 |
| regulation of ATPase activity | TPM2\|TPM1\|TNC | 2.98E-02 |
| negative regulation of cell-substrate adhesion | COL1A1\|LGALS1\|THBS1 | 2.98E-02 |
| regulation of protein catabolic process | VCP\|HSP90AB1\|PCSK9\|FLNA\|ADAM9\|TIMP1 | 2.98E-02 |
| response to external stimulus | PGLYRP2\|GSN\|HSPA5\|SERPINF1\|COL11A1\|PLAUR\|PCSK9\|MIF\|SOD2\|FGF2\|DCN\|COL1A1\|LDHA\|TF\|AXL\|ALB\|ENPP2\|IGFBP7\|CTSD\|CD44\|CTSB | 3.05E-02 |
| positive regulation of signal transduction | ECM1\|CSF1\|LIF\|MIF\|FGF2\|THBS1\|HPX\|TF\|LGALS1\|EEF1D\|FLNA\|CDH13\|CD44 | 3.15E-02 |
| morphogenesis of a branching epithelium | NRP1\|CSF1\|SEMA3C\|CTSZ\|DAG1\|FGF2\|CD44 | 3.17E-02 |
| generation of precursor metabolites and energy | GPI\|TPI1\|MDH1\|ATP6AP1\|MDH2\|GAA\|TXNL1\|ENO1\|SOD2\|LDHB\|LDHA\|PKM\|ALDOA\|GAPDH | 3.36E-02 |
| enzyme linked receptor protein signaling pathway | LIF\|LTBP2\|FSTL1\|FGF2\|CTGF\|TF\|COL3A1\|COL1A2\|AXL\|PDGFC\|FLNA\|ADAM9\|TKT\|AGRN\|FMOD | 3.40E-02 |
| positive regulation of coagulation | TF\|SERPINE1\|THBS1 | 3.40E-02 |
| protein-chromophore linkage | IGHA1\|NID1\|NID2 | 3.40E-02 |
| NADP metabolic process | TPI1\|TALDO1\|PGLS | 3.40E-02 |
| cardiac muscle contraction | GAA\|TPM1\|TNC | 3.40E-02 |
| branching involved in salivary gland morphogenesis | NRP1\|SEMA3C\|DAG1 | 3.40E-02 |
| cardiac chamber morphogenesis | SEMA3C\|GAA\|TPM1\|TNC | 3.54E-02 |
| positive regulation of signaling process | ECM1\|CSF1\|LIF\|MIF\|FGF2\|THBS1\|HPX\|TF\|LGALS1\|EEF1D\|FLNA\|CDH13\|CD44 | 3.56E-02 |
| coagulation | PROCR\|TF\|COL3A1\|EFEMP2\|ANXA5\|PLAUR\|SERPING1 | 3.62E-02 |
| blood coagulation | PROCR\|TF\|COL3A1\|EFEMP2\|ANXA5\|PLAUR\|SERPING1 | 3.62E-02 |
| response to carbohydrate stimulus | ACTR3\|LGALS1\|COL6A2\|THBS1\|CTSB\|CTGF | 3.65E-02 |
| cellular protein metabolic process | APP\|FKBP10\|HSP90AB1\|B4GALT1\|CD81\|RPLP1\|HEXB\|PCSK9\|PLOD3\|PLOD2\|PLOD1\|FGF2\|LOXL1\|LOXL2\|EEF1B2\|UCHL1\|CFL1\|RPLP2\|QSOX1\|RPS12\|DSP\|MANBA\|HSP90AA1\|ANXA1\|IGFBP3\|BGN\|PLAUR\|ADAM10\|RPSA\|DCN\|PSMA5\|PSMA6\|PSMA3\|LOX\|EEF1D\|ERP29\|ADAM9\|FKBP9\|TKT\|PPIB\|PPIA\|PPIC\|VCP\|NID1\|NID2\|THBS1\|HSP90B1\|PRDX2\|IGHA1\|LDLR\|HSPA5\|FN1\|TICRR\|COL3A1\|PTK7\|AXL\|QPCT\|CPE\|CALR\|P4HB | 3.67E-02 |
| positive regulation of molecular function | VCP\|CSF1\|HSPA5\|LRP1\|CD81\|TPM1\|HP\|APOA1\|ACTN4\|MIF\|THY1\|FGF2\|THBS1\|CTGF\|PRDX3\|PSMA5\|PRDX2\|PSMA6\|TF\|PSMA3\|PSAP\|ADAM9\|TPP1 | 3.83E-02 |
| gland morphogenesis | NRP1\|CSF1\|SEMA3C\|DAG1\|TNC\|CD44 | 3.83E-02 |
| regeneration | VCAN\|GSN\|AXL\|SERPINE1\|LIF\|PLAUR | 3.83E-02 |
| muscle fiber development | APP\|RCN1\|TNC\|AGRN | 3.83E-02 |
| neuromuscular junction development | APP\|TNC\|AGRN | 3.90E-02 |
| transforming growth factor beta receptor signaling pathway | COL3A1\|COL1A2\|ADAM9\|LTBP2\|FMOD | 3.91E-02 |
| positive regulation of intracellular protein kinase cascade | HPX\|TF\|ECM1\|LGALS1\|EEF1D\|LIF\|FLNA\|MIF\|FGF2\|THBS1\|CD44 | 4.01E-02 |
| regulation of neuron projection development | ACTR3\|NRP1\|LGALS1\|CDH2\|ARHGDIA\|XYLT1\|THY1 | 4.10E-02 |
| positive regulation of cell differentiation | ACTR3\|PNP\|SERPINE2\|CSF1\|SERPINF1\|ARHGDIA\|IGFBP3\|LIF\|VEGFC\|INHBA\|FGF2\|CLU | 4.10E-02 |
| iron ion homeostasis | HPX\|TF\|HP\|SOD2 | 4.10E-02 |
| biomineral formation | COL1A1\|DSP\|MINPP1\|ECM1 | 4.10E-02 |
| gland development | NRP1\|B4GALT1\|CSF1\|SEMA3C\|DAG1\|TNC\|APOA1\|FGF2\|CD44\|DKK3 | 4.15E-02 |
| response to cytokine stimulus | COL3A1\|SERPINE1\|MMP3\|XYLT1\|ADAM10\|ADAM9\|CTSB | 4.17E-02 |
| transmembrane receptor protein serine/threonine kinase signaling pathway | TF\|COL3A1\|COL1A2\|ADAM9\|LTBP2\|FMOD\|FSTL1 | 4.17E-02 |
| oxidation reduction | MOXD1\|MDH1\|MDH2\|TXNL1\|LOXL3\|PLOD3\|LOXL4\|PLOD2\|PLOD1\|SOD2\|PRDX6\|SOD3\|LOXL1\|LOXL2\|PRDX3\|LDHB\|PRDX2\|LDHA\|LOX\|PRDX1\|PXDN\|QSOX1\|GAPDH | 4.22E-02 |
| positive regulation of peptidyl-tyrosine phosphorylation | HPX\|CD81\|LIF\|MIF\|CD44 | 4.33E-02 |
| negative regulation of macromolecule metabolic process | YWHAE\|HSP90AB1\|SERPINE2\|LRP1\|SERPINE1\|PCSK9\|ENO1\|FGF2\|THBS1\|CST3\|YWHAQ\|BASP1\|CFL1\|FLNA\|ITGAV\|TIMP1\|IGFBP3\|MIF\|INHBA\|DKK3\|PSMA5\|PSMA6\|PSMA3\|SERPING1\|TPP1\|CALR\|MASP1 | 4.41E-02 |
| striated muscle contraction | GAA\|TPM1\|TNC\|ALDOA | 4.87E-02 |
| epithelial cell differentiation | DSP\|COL18A1\|ANXA1\|B4GALT1\|BASP1\|KRT2\|KRT14\|ADAM9\|FGF2 | 4.93E-02 |
| response to mechanical stimulus | COL1A1\|TF\|COL11A1\|DCN\|CTSB | 4.93E-02 |
| regulation of peptidyl-serine phosphorylation | LIF\|MIF\|CD44 | 4.93E-02 |
| regulation of phagocytosis | C3\|PTX3\|CALR | 4.93E-02 |
| response to amino acid stimulus | CFL1\|MMP3\|CTGF | 4.93E-02 |
| regulation of smooth muscle cell migration | TF\|IGFBP3\|SERPINE1 | 4.93E-02 |
| negative regulation of cellular protein metabolic process | PSMA5\|YWHAE\|PSMA6\|PSMA3\|HSP90AB1\|SERPINE2\|IGFBP3\|ITGAV\|CALR\|TIMP1 | 4.94E-02 |
