# Supplementary material for: Mass spectrometry analysis of adipose-derived stem cells reveals a significant effect of hypoxia on pathways regulating extracellular matrix
Source: Stem Cell Res Ther. 2016 Apr 14;7:52. doi: 10.1186/s13287-016-0310-7 (PMC4831147; doi:10.1186/s13287-016-0310-7)
Supplement: Additional file 3: Table S2. — Over-represented biological processes by gene ontology analysis of proteins identified in the peptidome fraction. (DOCX 39 kb) [file 13287_2016_310_MOESM3_ESM.docx]

**Supplemental Table 2. Overrepresented biological processes by gene ontology analysis of proteins identified in the peptidome fraction**

| Biological process | Genes coding for identified proteins (Gene names are given as HUGO gene nomenclature) | corr p-value |
| --- | --- | --- |
| extracellular matrix organization | POSTN\|LUM\|CYR61\|CTGF\|COL1A1\|CST3\|COL3A1\|COL1A2\|LOX\|COL5A1\|COL6A2\|SERPINH1\|TGFBI | 2.40E-11 |
| system development | MTPN\|SPARC\|AHNAK\|SERPINE1\|HP\|LTBP2\|ADM\|FGF2\|CLU\|THBS1\|CYR61\|CTGF\|ADAMTS4\|LGALS1\|CDH2\|BASP1\|LMNA\|SDF4\|TIMP1\|CTSB\|POSTN\|HSPA5\|IGFBP4\|KRT2\|SERPINF1\|MMP2\|KRT1\|TPM1\|FN1\|INHBA\|KRT10\|HSPG2\|KRT9\|COL1A1\|VCAN\|COL3A1\|COL1A2\|LOX\|COL5A1\|CDH11\|KRT14\|SDC1\|COL6A3\|PFN1\|FBN1 | 2.40E-11 |
| multicellular organismal development | SPARC\|SERPINE1\|HP\|ADM\|FGF2\|CLU\|CYR61\|CTGF\|ADAMTS4\|LGALS1\|CDH2\|BASP1\|SDF4\|TIMP1\|CTSB\|POSTN\|IGFBP4\|KRT2\|SERPINF1\|MMP2\|KRT1\|TPM1\|ADAM10\|PCOLCE\|HSPG2\|KRT9\|VCAN\|LOX\|CDH11\|COL6A3\|PFN1\|FBN2\|MTPN\|AHNAK\|LTBP2\|THBS1\|OLFML3\|LMNA\|HSPA5\|FN1\|INHBA\|KRT10\|COL1A1\|COL3A1\|COL1A2\|COL5A1\|KRT14\|SDC1\|FBN1 | 5.11E-11 |
| anatomical structure development | FBN2\|MTPN\|SPARC\|AHNAK\|SERPINE1\|HP\|LTBP2\|ADM\|FGF2\|CLU\|THBS1\|CYR61\|CTGF\|ADAMTS4\|LGALS1\|CDH2\|BASP1\|LMNA\|SDF4\|TIMP1\|CTSB\|POSTN\|HSPA5\|IGFBP4\|KRT2\|SERPINF1\|MMP2\|KRT1\|TPM1\|FN1\|INHBA\|KRT10\|HSPG2\|KRT9\|COL1A1\|VCAN\|COL3A1\|COL1A2\|LOX\|COL5A1\|CDH11\|KRT14\|SDC1\|COL6A3\|PFN1\|FBN1 | 7.08E-11 |
| extracellular structure organization | POSTN\|LUM\|CYR61\|CTGF\|COL1A1\|CST3\|COL3A1\|COL1A2\|CDH2\|LOX\|COL5A1\|COL6A2\|SERPINH1\|TGFBI | 1.52E-10 |
| developmental process | SPARC\|SERPINE1\|HP\|ADM\|FGF2\|CLU\|CYR61\|CTGF\|ADAMTS4\|LGALS1\|CDH2\|BASP1\|SDF4\|TIMP1\|CTSB\|POSTN\|IGFBP4\|KRT2\|SERPINF1\|MMP2\|KRT1\|TPM1\|ADAM10\|PCOLCE\|HSPG2\|KRT9\|VCAN\|LOX\|CDH11\|COL6A3\|PFN1\|FBN2\|MTPN\|AHNAK\|LTBP2\|THBS1\|OLFML3\|LMNA\|HSPA5\|FN1\|INHBA\|KRT10\|COL1A1\|TF\|COL3A1\|COL1A2\|COL5A1\|KRT14\|SDC1\|FBN1 | 1.52E-10 |
| organ development | MTPN\|SERPINE1\|HP\|ADM\|FGF2\|CLU\|THBS1\|CYR61\|CTGF\|CDH2\|BASP1\|LMNA\|SDF4\|TIMP1\|CTSB\|POSTN\|HSPA5\|KRT2\|SERPINF1\|MMP2\|KRT1\|TPM1\|FN1\|INHBA\|KRT10\|HSPG2\|KRT9\|COL1A1\|COL3A1\|COL1A2\|LOX\|COL5A1\|KRT14\|SDC1\|COL6A3\|PFN1\|FBN1 | 2.33E-10 |
| multicellular organismal process | SPARC\|SERPINE1\|HP\|ADM\|FGF2\|CLU\|CYR61\|CTGF\|ADAMTS4\|LGALS1\|CDH2\|BASP1\|SDF4\|TIMP1\|CTSB\|POSTN\|TPM4\|IGFBP4\|KRT2\|SERPINF1\|MMP2\|KRT1\|TPM1\|ADAM10\|PCOLCE\|HSPG2\|KRT9\|VCAN\|LOX\|CDH11\|COL6A3\|PFN1\|FBN2\|MTPN\|CSTB\|AHNAK\|LTBP2\|THBS1\|OLFML3\|LMNA\|SERPINH1\|HSPA5\|LUM\|FN1\|APOC3\|INHBA\|KRT10\|COL1A1\|TF\|COL3A1\|COL1A2\|COL5A1\|KRT14\|SDC1\|TGFBI\|FBN1 | 3.63E-09 |
| response to wounding | MTPN\|IGFBP4\|SERPINE1\|KRT1\|TPM1\|CFI\|FN1\|ADM\|FGF2\|CLU\|THBS1\|CTGF\|TF\|VCAN\|COL3A1\|LGALS1\|LOX\|COL5A1\|SDC1\|CTSB | 5.94E-09 |
| collagen fibril organization | COL1A1\|COL3A1\|COL1A2\|LOX\|COL5A1\|LUM\|SERPINH1 | 2.43E-08 |
| tissue development | MTPN\|POSTN\|KRT2\|SERPINE1\|KRT1\|TPM1\|HP\|ADM\|INHBA\|KRT10\|FGF2\|CTGF\|KRT9\|COL1A1\|COL3A1\|COL1A2\|COL5A1\|BASP1\|LMNA\|KRT14\|PFN1\|CTSB | 3.71E-08 |
| response to organic substance | HSPA5\|SERPINF1\|SERPINE1\|HP\|ADAM10\|HSPB1\|ADM\|CLU\|THBS1\|CYR61\|CTGF\|COL1A1\|TF\|COL3A1\|LGALS1\|LOX\|COL6A2\|SERPINH1\|SDC1\|SDF4\|PFN1\|B2M\|CTSB | 9.18E-08 |
| response to chemical stimulus | SERPINE1\|HP\|HSPB1\|ADM\|FGF2\|CLU\|THBS1\|CYR61\|CTGF\|LGALS1\|SERPINH1\|SDF4\|B2M\|CTSB\|TPM4\|HSPA5\|SERPINF1\|MMP2\|KRT1\|TPM1\|ADAM10\|COL1A1\|TF\|COL3A1\|LOX\|COL6A2\|KRT14\|SDC1\|PFN1 | 2.63E-07 |
| blood vessel development | MMP2\|FN1\|FGF2\|HSPG2\|THBS1\|CYR61\|CTGF\|COL1A1\|COL3A1\|COL1A2\|CDH2\|LOX\|COL5A1 | 5.39E-07 |
| collagen biosynthetic process | COL1A1\|COL3A1\|COL5A1\|SERPINH1 | 5.70E-07 |
| vasculature development | MMP2\|FN1\|FGF2\|HSPG2\|THBS1\|CYR61\|CTGF\|COL1A1\|COL3A1\|COL1A2\|CDH2\|LOX\|COL5A1 | 6.74E-07 |
| response to stress | MTPN\|SERPINE1\|CFI\|HP\|HSPB1\|ADM\|FGF2\|CLU\|THBS1\|CTGF\|CST3\|LGALS1\|SERPINH1\|CTSB\|TPM4\|HSPA5\|IGFBP4\|MMP2\|KRT1\|TPM1\|FN1\|INHBA\|COL1A1\|TF\|VCAN\|COL3A1\|LOX\|COL5A1\|ALB\|SDC1\|PFN1 | 9.12E-07 |
| epidermis development | COL1A1\|COL3A1\|COL1A2\|COL5A1\|KRT2\|KRT1\|KRT14\|INHBA\|KRT10\|CTGF\|KRT9 | 1.02E-06 |
| ectoderm development | COL1A1\|COL3A1\|COL1A2\|COL5A1\|KRT2\|KRT1\|KRT14\|INHBA\|KRT10\|CTGF\|KRT9 | 2.27E-06 |
| skeletal system development | POSTN\|SPARC\|IGFBP4\|MMP2\|LTBP2\|FGF2\|CTGF\|COL1A1\|ADAMTS4\|COL3A1\|COL1A2\|CDH11\|FBN1 | 5.46E-06 |
| regulation of peptidase activity | CST3\|CSTB\|TF\|HSPA5\|SERPINE1\|HP\|THBS1\|CTGF | 1.18E-05 |
| anatomical structure formation involved in morphogenesis | MTPN\|MMP2\|TPM1\|HP\|FN1\|ADM\|FGF2\|HSPG2\|THBS1\|CYR61\|CTGF\|COL1A1\|PFN1 | 2.03E-05 |
| negative regulation of cell adhesion | COL1A1\|LGALS1\|SERPINE1\|ADAM10\|TGFBI\|THBS1 | 2.38E-05 |
| anatomical structure morphogenesis | FBN2\|MTPN\|HSPA5\|MMP2\|SERPINE1\|TPM1\|HP\|FN1\|ADM\|INHBA\|FGF2\|CLU\|HSPG2\|THBS1\|CYR61\|CTGF\|COL1A1\|VCAN\|COL1A2\|CDH2\|COL5A1\|SDC1\|PFN1 | 2.51E-05 |
| negative regulation of peptidase activity | CST3\|CSTB\|HSPA5\|SERPINE1\|THBS1 | 3.43E-05 |
| collagen metabolic process | COL1A1\|COL3A1\|COL5A1\|MMP2\|SERPINH1 | 4.74E-05 |
| negative regulation of hydrolase activity | CST3\|CSTB\|HSPA5\|SERPINE1\|APOC3\|THBS1 | 6.38E-05 |
| multicellular organismal macromolecule metabolic process | COL1A1\|COL3A1\|COL5A1\|MMP2\|SERPINH1 | 7.23E-05 |
| skin development | COL1A1\|COL3A1\|COL1A2\|COL5A1\|KRT9 | 8.15E-05 |
| regulation of cell adhesion | COL1A1\|LGALS1\|SERPINE1\|TPM1\|ADAM10\|TGFBI\|THBS1\|CYR61 | 1.29E-04 |
| wound healing | MTPN\|TF\|COL3A1\|LOX\|COL5A1\|SERPINE1\|TPM1\|SDC1\|FGF2 | 1.29E-04 |
| multicellular organismal metabolic process | COL1A1\|COL3A1\|COL5A1\|MMP2\|SERPINH1 | 1.49E-04 |
| cellular component movement | TPM4\|KRT2\|TPM1\|FN1\|HSPB1\|FGF2\|THBS1\|CTGF\|VCAN\|CDH2\|COL5A1\|VIM\|CTHRC1 | 1.85E-04 |
| protein maturation | TF\|KRT1\|CFI\|SCG5\|SERPINH1\|CPE\|CLU | 2.31E-04 |
| fibril organization | CST3\|COL3A1\|COL5A1 | 2.94E-04 |
| response to steroid hormone stimulus | COL1A1\|TF\|LOX\|SERPINF1\|SERPINE1\|SDC1\|ADM\|THBS1\|CTGF | 3.02E-04 |
| protein maturation by peptide bond cleavage | TF\|KRT1\|CFI\|SCG5\|CPE\|CLU | 3.35E-04 |
| negative regulation of cellular component organization | CST3\|LGALS1\|TMSB4X\|HP\|APOC3\|CLU\|TMSB10\|THBS1 | 3.91E-04 |
| regulation of anatomical structure morphogenesis | CST3\|TF\|CDH2\|SERPINF1\|SERPINE1\|KRT1\|HP\|FN1\|FGF2\|THBS1 | 4.32E-04 |
| response to stimulus | MTPN\|CSTB\|SERPINE1\|CFI\|HP\|HSPB1\|ADM\|FGF2\|CLU\|THBS1\|CYR61\|CTGF\|CST3\|LGALS1\|SERPINH1\|SDF4\|B2M\|CTSB\|TPM4\|HSPA5\|IGFBP4\|SERPINF1\|MMP2\|KRT1\|TPM1\|FN1\|ADAM10\|INHBA\|COL1A1\|TF\|VCAN\|COL3A1\|LOX\|COL5A1\|COL6A2\|ALB\|KRT14\|SDC1\|TGFBI\|PFN1 | 4.57E-04 |
| regulation of angiogenesis | TF\|SERPINF1\|SERPINE1\|KRT1\|FGF2\|THBS1 | 4.57E-04 |
| response to oxidative stress | COL1A1\|TF\|TPM4\|SERPINE1\|KRT1\|TPM1\|SDC1\|CLU | 4.69E-04 |
| regulation of cellular component organization | SERPINE1\|TPM1\|HP\|FN1\|APOC3\|CLU\|THBS1\|CST3\|LGALS1\|CDH2\|COL5A1\|TMSB4X\|TMSB10 | 5.55E-04 |
| cell adhesion | POSTN\|HP\|FN1\|HSPG2\|THBS1\|CYR61\|CTGF\|VCAN\|COL3A1\|CDH2\|COL5A1\|COL6A2\|COL6A1\|CDH11\|COL6A3 | 5.64E-04 |
| biological adhesion | POSTN\|HP\|FN1\|HSPG2\|THBS1\|CYR61\|CTGF\|VCAN\|COL3A1\|CDH2\|COL5A1\|COL6A2\|COL6A1\|CDH11\|COL6A3 | 5.64E-04 |
| regulation of endopeptidase activity | TF\|HSPA5\|SERPINE1\|HP\|THBS1\|CTGF | 7.97E-04 |
| negative regulation of biological process | SERPINE1\|HP\|HSPB1\|ADM\|FGF2\|CLU\|THBS1\|CST3\|LGALS1\|BASP1\|TMSB4X\|IGFBP6\|TIMP1\|TMSB10\|CTSB\|HSPA5\|SERPINF1\|KRT1\|TPM1\|ADAM10\|APOC3\|INHBA\|COL1A1\|TF\|COL3A1\|ALB\|TGFBI | 7.97E-04 |
| positive regulation of response to stimulus | HPX\|TF\|SERPINE1\|KRT1\|CFI\|ADAM10\|CLU\|B2M\|THBS1 | 9.11E-04 |
| response to glucose stimulus | LGALS1\|COL6A2\|THBS1\|CTGF\|CTSB | 9.73E-04 |
| angiogenesis | MMP2\|FN1\|FGF2\|HSPG2\|THBS1\|CYR61\|CTGF | 9.73E-04 |
| response to corticosteroid stimulus | COL1A1\|SERPINF1\|SERPINE1\|SDC1\|ADM\|CTGF | 9.98E-04 |
| protein processing | TF\|KRT1\|CFI\|SCG5\|CPE\|CLU | 1.03E-03 |
| cell migration | VCAN\|CDH2\|COL5A1\|KRT2\|FN1\|FGF2\|THBS1\|CTGF\|CTHRC1 | 1.16E-03 |
| response to monosaccharide stimulus | LGALS1\|COL6A2\|THBS1\|CTGF\|CTSB | 1.16E-03 |
| response to hexose stimulus | LGALS1\|COL6A2\|THBS1\|CTGF\|CTSB | 1.16E-03 |
| negative regulation of cellular process | SERPINE1\|HP\|HSPB1\|ADM\|FGF2\|CLU\|THBS1\|CST3\|LGALS1\|BASP1\|TMSB4X\|IGFBP6\|TIMP1\|TMSB10\|CTSB\|HSPA5\|SERPINF1\|TPM1\|ADAM10\|APOC3\|INHBA\|COL1A1\|TF\|ALB\|TGFBI | 1.16E-03 |
| negative regulation of cell migration | SERPINE1\|TPM1\|HP\|FGF2\|THBS1 | 1.20E-03 |
| blood vessel morphogenesis | CDH2\|MMP2\|FN1\|FGF2\|HSPG2\|THBS1\|CYR61\|CTGF | 1.20E-03 |
| response to hormone stimulus | COL1A1\|TF\|LOX\|SERPINF1\|SERPINE1\|HP\|SDC1\|ADM\|THBS1\|CTGF\|CTSB | 1.42E-03 |
| negative regulation of cellular component movement | SERPINE1\|TPM1\|HP\|FGF2\|THBS1 | 1.58E-03 |
| negative regulation of locomotion | SERPINE1\|TPM1\|HP\|FGF2\|THBS1 | 1.58E-03 |
| response to protein stimulus | HSPA5\|SERPINH1\|HSPB1\|CLU\|CYR61\|CTSB | 1.58E-03 |
| regulation of biological quality | MTPN\|SERPINE1\|TPM1\|HP\|FN1\|ADAM10\|APOC3\|ADM\|INHBA\|FGF2\|HPX\|TF\|COL3A1\|COL1A2\|TMSB4X\|ALB\|SCG5\|CPE\|SH3BGRL3\|TIMP1\|P4HB\|TMSB10 | 1.58E-03 |
| positive regulation of blood coagulation | TF\|SERPINE1\|THBS1 | 1.60E-03 |
| regulation of hydrolase activity | CST3\|CSTB\|TF\|HSPA5\|SERPINE1\|TPM1\|HP\|APOC3\|THBS1\|CTGF | 1.66E-03 |
| regulation of blood coagulation | TF\|SERPINE1\|KRT1\|THBS1 | 1.80E-03 |
| response to inorganic substance | COL1A1\|TF\|SERPINE1\|TPM1\|KRT14\|SDC1\|B2M\|THBS1 | 1.82E-03 |
| cellular component organization | HP\|ADM\|DBI\|CLU\|THBS1\|CYR61\|CTGF\|CST3\|CDH2\|TMSB4X\|LMNA\|SERPINH1\|TMSB10\|POSTN\|LUM\|TPM1\|FN1\|APOC3\|TBCA\|KRT9\|COL1A1\|VCAN\|COL3A1\|COL1A2\|LOX\|COL5A1\|COL6A2\|KRT14\|TGFBI\|PFN1 | 2.01E-03 |
| response to carbohydrate stimulus | LGALS1\|COL6A2\|THBS1\|CTGF\|CTSB | 2.01E-03 |
| activation of plasma proteins involved in acute inflammatory response | TF\|KRT1\|CFI\|CLU | 2.01E-03 |
| positive regulation of chemotaxis | TF\|SERPINE1\|ADAM10\|THBS1 | 2.01E-03 |
| localization of cell | VCAN\|CDH2\|COL5A1\|KRT2\|FN1\|FGF2\|THBS1\|CTGF\|CTHRC1 | 2.01E-03 |
| cell motility | VCAN\|CDH2\|COL5A1\|KRT2\|FN1\|FGF2\|THBS1\|CTGF\|CTHRC1 | 2.01E-03 |
| negative regulation of cell-substrate adhesion | COL1A1\|LGALS1\|THBS1 | 2.09E-03 |
| positive regulation of angiogenesis | TF\|SERPINE1\|FGF2\|THBS1 | 2.25E-03 |
| regulation of wound healing | TF\|SERPINE1\|KRT1\|THBS1 | 2.25E-03 |
| regulation of coagulation | TF\|SERPINE1\|KRT1\|THBS1 | 2.25E-03 |
| positive regulation of coagulation | TF\|SERPINE1\|THBS1 | 2.39E-03 |
| response to endogenous stimulus | COL1A1\|TF\|LOX\|SERPINF1\|SERPINE1\|HP\|SDC1\|ADM\|THBS1\|CTGF\|CTSB | 2.83E-03 |
| response to reactive oxygen species | COL1A1\|TF\|SERPINE1\|TPM1\|SDC1 | 2.83E-03 |
| positive regulation of behavior | TF\|SERPINE1\|ADAM10\|THBS1 | 3.04E-03 |
| positive regulation of immune system process | HPX\|SERPINE1\|KRT1\|CFI\|ADAM10\|CLU\|B2M\|THBS1 | 3.09E-03 |
| acute inflammatory response | TF\|KRT1\|CFI\|FN1\|CLU | 3.21E-03 |
| regulation of immune system process | HPX\|COL3A1\|SERPINE1\|KRT1\|CFI\|ADAM10\|INHBA\|CLU\|B2M\|THBS1 | 3.48E-03 |
| regulation of caspase activity | TF\|HSPA5\|HP\|THBS1\|CTGF | 3.61E-03 |
| regulation of developmental process | MTPN\|SERPINF1\|SERPINE1\|KRT1\|HP\|FN1\|INHBA\|FGF2\|CLU\|THBS1\|CST3\|TF\|LGALS1\|CDH2 | 3.61E-03 |
| regulation of chemotaxis | TF\|SERPINE1\|ADAM10\|THBS1 | 3.85E-03 |
| positive regulation of multicellular organismal process | TF\|SERPINE1\|TPM1\|ADM\|INHBA\|FGF2\|THBS1\|CTGF | 3.95E-03 |
| intermediate filament-based process | KRT14\|VIM\|KRT9 | 4.00E-03 |
| regulation of response to stimulus | HPX\|TF\|COL3A1\|SERPINF1\|SERPINE1\|KRT1\|CFI\|ADAM10\|CLU\|B2M\|THBS1 | 4.15E-03 |
| regulation of cell growth | IGFBP4\|ADAM10\|IGFBP6\|INHBA\|FGF2\|CYR61\|CTGF | 4.15E-03 |
| locomotion | VCAN\|CDH2\|COL5A1\|KRT2\|FN1\|FGF2\|THBS1\|CYR61\|CTGF\|CTHRC1 | 4.25E-03 |
| defense response | CST3\|TF\|IGFBP4\|SERPINE1\|KRT1\|CFI\|HP\|FN1\|INHBA\|PFN1\|CLU\|THBS1 | 4.42E-03 |
| regulation of cell-substrate adhesion | COL1A1\|LGALS1\|THBS1\|CYR61 | 4.46E-03 |
| regulation of cell migration | TF\|SERPINE1\|TPM1\|HP\|ADAM10\|FGF2\|THBS1 | 4.46E-03 |
| positive regulation of leukocyte chemotaxis | SERPINE1\|ADAM10\|THBS1 | 4.86E-03 |
| hindbrain morphogenesis | MTPN\|HSPA5\|HP | 4.86E-03 |
| heart development | COL3A1\|COL5A1\|TPM1\|LMNA\|HP\|ADM\|FBN1 | 4.95E-03 |
| regulation of cellular component biogenesis | TMSB4X\|TPM1\|HP\|CLU\|TMSB10\|THBS1 | 5.00E-03 |
| organ morphogenesis | COL1A1\|COL1A2\|COL5A1\|MMP2\|TPM1\|HP\|SDC1\|ADM\|INHBA\|PFN1\|FGF2\|CTGF | 5.03E-03 |
| hindbrain development | MTPN\|HSPA5\|HP\|SDF4 | 5.38E-03 |
| regulation of cell proliferation | SPARC\|SERPINF1\|SERPINE1\|HP\|ADAM10\|ADM\|INHBA\|FGF2\|CLU\|THBS1\|CTGF\|TF\|IGFBP6\|TIMP1 | 6.10E-03 |
| peptide cross-linking | COL3A1\|FN1\|THBS1 | 6.53E-03 |
| negative regulation of cell death | TF\|HSPA5\|SERPINE1\|ALB\|HSPB1\|FGF2\|CLU\|THBS1\|CTSB | 6.69E-03 |
| regulation of programmed cell death | CSTB\|HSPA5\|SERPINE1\|HP\|HSPB1\|INHBA\|FGF2\|CLU\|THBS1\|CTGF\|TF\|LGALS1\|ALB\|CTSB | 6.83E-03 |
| negative regulation of cellular catabolic process | CST3\|APOC3\|TIMP1 | 7.05E-03 |
| regulation of cellular component movement | TF\|SERPINE1\|TPM1\|HP\|ADAM10\|FGF2\|THBS1 | 7.11E-03 |
| regulation of cell death | CSTB\|HSPA5\|SERPINE1\|HP\|HSPB1\|INHBA\|FGF2\|CLU\|THBS1\|CTGF\|TF\|LGALS1\|ALB\|CTSB | 7.15E-03 |
| odontogenesis | COL1A1\|COL1A2\|SDC1\|INHBA | 7.23E-03 |
| regulation of locomotion | TF\|SERPINE1\|TPM1\|HP\|ADAM10\|FGF2\|THBS1 | 7.28E-03 |
| negative regulation of multicellular organismal process | CST3\|SERPINE1\|KRT1\|APOC3\|ADM\|THBS1 | 7.42E-03 |
| regulation of leukocyte chemotaxis | SERPINE1\|ADAM10\|THBS1 | 7.46E-03 |
| ossification | COL1A1\|SPARC\|MMP2\|CDH11\|CTGF | 7.75E-03 |
| muscle cell differentiation | MTPN\|LGALS1\|TPM1\|LMNA\|SDC1 | 7.75E-03 |
| response to abiotic stimulus | COL1A1\|TF\|COL3A1\|KRT14\|HSPB1\|SDF4\|ADM\|THBS1\|CTSB | 7.91E-03 |
| cerebellum development | MTPN\|HSPA5\|SDF4 | 8.65E-03 |
| positive regulation of cell adhesion | LGALS1\|TPM1\|THBS1\|CYR61 | 8.71E-03 |
| regulation of behavior | TF\|SERPINE1\|ADAM10\|THBS1 | 8.71E-03 |
| negative regulation of angiogenesis | SERPINF1\|SERPINE1\|THBS1 | 9.25E-03 |
| positive regulation of leukocyte migration | SERPINE1\|ADAM10\|THBS1 | 9.25E-03 |
| regulation of response to external stimulus | TF\|SERPINF1\|SERPINE1\|KRT1\|ADAM10\|THBS1 | 1.08E-02 |
| tube closure | HP\|ADM\|PFN1 | 1.08E-02 |
| neural tube closure | HP\|ADM\|PFN1 | 1.08E-02 |
| positive regulation of cell migration | TF\|SERPINE1\|ADAM10\|FGF2\|THBS1 | 1.08E-02 |
| muscle structure development | MTPN\|LGALS1\|TPM1\|LMNA\|SDC1\|COL6A3\|CTSB | 1.09E-02 |
| bone development | COL1A1\|SPARC\|MMP2\|CDH11\|CTGF | 1.18E-02 |
| metencephalon development | MTPN\|HSPA5\|SDF4 | 1.25E-02 |
| regulation of localization | TF\|CDH2\|SERPINE1\|TPM1\|HP\|SCG5\|ADAM10\|APOC3\|DBI\|INHBA\|FGF2\|THBS1 | 1.26E-02 |
| positive regulation of phosphorus metabolic process | HPX\|HP\|FGF2\|THBS1\|CTGF | 1.27E-02 |
| positive regulation of phosphate metabolic process | HPX\|HP\|FGF2\|THBS1\|CTGF | 1.27E-02 |
| tissue morphogenesis | COL1A1\|COL1A2\|TPM1\|HP\|ADM\|PFN1\|FGF2 | 1.29E-02 |
| regeneration | MTPN\|VCAN\|SERPINE1\|ADM | 1.29E-02 |
| primary neural tube formation | HP\|ADM\|PFN1 | 1.29E-02 |
| iron ion homeostasis | HPX\|TF\|HP | 1.29E-02 |
| positive regulation of cellular component movement | TF\|SERPINE1\|ADAM10\|FGF2\|THBS1 | 1.35E-02 |
| positive regulation of locomotion | TF\|SERPINE1\|ADAM10\|FGF2\|THBS1 | 1.35E-02 |
| regulation of multicellular organismal process | MTPN\|SERPINF1\|SERPINE1\|KRT1\|TPM1\|APOC3\|ADM\|INHBA\|FGF2\|THBS1\|CTGF\|CST3\|TF\|LGALS1\|CDH2 | 1.44E-02 |
| regulation of apoptosis | CSTB\|HSPA5\|SERPINE1\|HP\|HSPB1\|INHBA\|CLU\|THBS1\|CTGF\|TF\|LGALS1\|ALB\|CTSB | 1.46E-02 |
| cell differentiation | MTPN\|KRT2\|SERPINF1\|TPM1\|FN1\|ADM\|INHBA\|FGF2\|CLU\|CTGF\|COL1A1\|VCAN\|LGALS1\|CDH2\|BASP1\|LMNA\|KRT14\|SDC1\|SDF4\|TIMP1 | 1.46E-02 |
| regulation of lipid transport | APOC3\|DBI\|THBS1 | 1.50E-02 |
| striated muscle cell differentiation | MTPN\|TPM1\|LMNA\|SDC1 | 1.57E-02 |
| negative regulation of protein complex assembly | TMSB4X\|CLU\|TMSB10 | 1.57E-02 |
| regulation of leukocyte migration | SERPINE1\|ADAM10\|THBS1 | 1.57E-02 |
| complement activation | KRT1\|CFI\|CLU | 1.57E-02 |
| aging | TF\|SERPINF1\|SERPINE1\|ADM\|CTGF | 1.57E-02 |
| positive regulation of immune response | HPX\|KRT1\|CFI\|CLU\|B2M | 1.61E-02 |
| positive regulation of response to external stimulus | TF\|SERPINE1\|ADAM10\|THBS1 | 1.61E-02 |
| tube development | LOX\|HP\|SDC1\|ADM\|PFN1\|FGF2\|CTGF | 1.71E-02 |
| neural tube formation | HP\|ADM\|PFN1 | 1.86E-02 |
| cellular developmental process | MTPN\|KRT2\|SERPINF1\|TPM1\|FN1\|ADM\|INHBA\|FGF2\|CLU\|CTGF\|COL1A1\|VCAN\|LGALS1\|CDH2\|BASP1\|LMNA\|KRT14\|SDC1\|SDF4\|TIMP1 | 1.88E-02 |
| regulation of hormone levels | SCG5\|CPE\|ADM\|INHBA\|FGF2 | 1.89E-02 |
| regulation of actin cytoskeleton organization | TMSB4X\|TPM1\|HP\|TMSB10 | 2.04E-02 |
| cellular process | SERPINE1\|HP\|HSPB1\|ADM\|DBI\|FGF2\|CLU\|CYR61\|CTGF\|CA1\|LGALS1\|CDH2\|BASP1\|TMSB4X\|SDF4\|SH3BGRL3\|TIMP1\|TMSB10\|CTSB\|POSTN\|TPM4\|IGFBP4\|KRT2\|SERPINF1\|TPM1\|ADAM10\|HSPG2\|KRT9\|VCAN\|LOX\|COL6A2\|COL6A1\|CDH11\|SCG5\|COL6A3\|PFN1\|PPIB\|PPIA\|PTMA\|MTPN\|FKBP2\|LTBP2\|THBS1\|CST3\|HPX\|LMNA\|SERPINH1\|CTHRC1\|HSPA5\|LUM\|FN1\|APOC3\|TBCA\|INHBA\|COL1A1\|TF\|COL3A1\|COL1A2\|COL5A1\|FASN\|ALB\|KRT14\|CPE\|SDC1\|TGFBI\|P4HB\|VIM\|RPS21 | 2.13E-02 |
| embryonic epithelial tube formation | HP\|ADM\|PFN1 | 2.18E-02 |
| response to glucocorticoid stimulus | SERPINF1\|SERPINE1\|SDC1\|ADM | 2.22E-02 |
| inflammatory response | TF\|IGFBP4\|KRT1\|CFI\|FN1\|CLU\|THBS1 | 2.23E-02 |
| epithelial tube formation | HP\|ADM\|PFN1 | 2.23E-02 |
| negative regulation of catabolic process | CST3\|APOC3\|TIMP1 | 2.23E-02 |
| regulation of immune response | HPX\|COL3A1\|KRT1\|CFI\|CLU\|B2M | 2.23E-02 |
| regulation of actin filament-based process | TMSB4X\|TPM1\|HP\|TMSB10 | 2.23E-02 |
| response to temperature stimulus | TF\|HSPB1\|ADM\|THBS1 | 2.23E-02 |
| cell-substrate adhesion | COL3A1\|HP\|FN1\|CTGF | 2.30E-02 |
| tube formation | HP\|ADM\|PFN1 | 2.58E-02 |
| transmembrane receptor protein serine/threonine kinase signaling pathway | TF\|COL3A1\|COL1A2\|LTBP2 | 2.91E-02 |
| response to cytokine stimulus | COL3A1\|SERPINE1\|ADAM10\|CTSB | 2.91E-02 |
| epithelium development | BASP1\|KRT2\|KRT14\|HP\|ADM\|PFN1\|FGF2 | 2.99E-02 |
| protein metabolic process | FKBP2\|CFI\|HP\|FGF2\|CLU\|THBS1\|ADAMTS4\|HPX\|SERPINH1\|CTSB\|HSPA5\|MMP2\|KRT1\|FN1\|ADAM10\|TBCA\|INHBA\|TF\|COL3A1\|LOX\|SCG5\|CPE\|P4HB\|PPIB\|PPIA\|RPS21 | 3.01E-02 |
| hormone metabolic process | SCG5\|CPE\|ADM\|FGF2 | 3.15E-02 |
| muscle cell development | TPM1\|LMNA\|SDC1 | 3.21E-02 |
| regulation of protein metabolic process | HPX\|MTPN\|HSPA5\|SERPINE1\|HP\|HSPB1\|TIMP1\|FGF2\|THBS1\|CTGF | 3.24E-02 |
| transforming growth factor beta receptor signaling pathway | COL3A1\|COL1A2\|LTBP2 | 3.28E-02 |
| positive regulation of developmental process | TF\|SERPINF1\|SERPINE1\|INHBA\|FGF2\|CLU\|THBS1 | 3.29E-02 |
| integrin-mediated signaling pathway | COL3A1\|ADAM10\|CTGF | 3.29E-02 |
| response to starvation | HSPA5\|ALB\|ADM | 3.29E-02 |
| response to biotic stimulus | TF\|HSPA5\|SERPINE1\|SERPINH1\|HSPB1\|ADM\|CLU\|B2M | 3.29E-02 |
| response to peptide hormone stimulus | COL1A1\|HP\|ADM\|CTGF\|CTSB | 3.29E-02 |
| response to external stimulus | COL1A1\|TF\|HSPA5\|SERPINF1\|ALB\|ADM\|FGF2\|CYR61\|CTSB | 3.29E-02 |
| response to mechanical stimulus | COL1A1\|TF\|CTSB | 3.29E-02 |
| regulation of growth | IGFBP4\|ADAM10\|IGFBP6\|INHBA\|FGF2\|CYR61\|CTGF | 3.29E-02 |
| regulation of cellular protein metabolic process | HPX\|MTPN\|HSPA5\|HP\|HSPB1\|TIMP1\|FGF2\|THBS1\|CTGF | 3.29E-02 |
| negative regulation of cytoskeleton organization | TMSB4X\|HP\|TMSB10 | 3.29E-02 |
| positive regulation of hydrolase activity | TF\|HSPA5\|TPM1\|HP\|CTGF | 3.53E-02 |
| anti-apoptosis | TF\|HSPA5\|HSPB1\|CLU\|THBS1 | 3.60E-02 |
| epithelial tube morphogenesis | HP\|ADM\|PFN1\|FGF2 | 3.63E-02 |
| regulation of cellular component size | MTPN\|TMSB4X\|ADAM10\|INHBA\|FGF2\|TMSB10 | 3.63E-02 |
| response to molecule of bacterial origin | TF\|SERPINE1\|ADM\|B2M | 3.67E-02 |
| negative regulation of apoptosis | TF\|HSPA5\|SERPINE1\|ALB\|HSPB1\|CLU\|THBS1 | 3.70E-02 |
| response to unfolded protein | HSPA5\|SERPINH1\|HSPB1 | 3.74E-02 |
| regulation of catalytic activity | CST3\|CSTB\|TF\|HSPA5\|SERPINE1\|TPM1\|HP\|APOC3\|FGF2\|THBS1\|CTGF\|CTSB | 3.76E-02 |
| negative regulation of cell proliferation | SERPINF1\|HP\|ADM\|IGFBP6\|INHBA\|FGF2\|THBS1 | 3.82E-02 |
| negative regulation of programmed cell death | TF\|HSPA5\|SERPINE1\|ALB\|HSPB1\|CLU\|THBS1 | 3.90E-02 |
| positive regulation of cellular protein metabolic process | HPX\|HSPA5\|HP\|FGF2\|THBS1\|CTGF | 3.93E-02 |
| positive regulation of phosphorylation | HPX\|FGF2\|THBS1\|CTGF | 3.94E-02 |
| nervous system development | MTPN\|HSPA5\|AHNAK\|SERPINF1\|HP\|ADM\|INHBA\|FGF2\|CLU\|VCAN\|LGALS1\|CDH2\|SDF4\|PFN1 | 3.98E-02 |
| morphogenesis of embryonic epithelium | HP\|ADM\|PFN1 | 4.08E-02 |
| negative regulation of catalytic activity | CST3\|CSTB\|HSPA5\|SERPINE1\|APOC3\|THBS1 | 4.40E-02 |
| neural tube development | HP\|ADM\|PFN1 | 4.44E-02 |
| cellular response to chemical stimulus | COL1A1\|TF\|LGALS1\|HSPA5\|SERPINE1\|TPM1\|HP | 4.69E-02 |
| positive regulation of protein metabolic process | HPX\|HSPA5\|HP\|FGF2\|THBS1\|CTGF | 4.81E-02 |
| positive regulation of intracellular protein kinase cascade | HPX\|TF\|LGALS1\|FGF2\|THBS1 | 4.86E-02 |
| regulation of cytoskeleton organization | TMSB4X\|TPM1\|HP\|TMSB10 | 4.86E-02 |
| response to ethanol | SDF4\|PFN1\|CTSB | 4.86E-02 |
| cellular response to nutrient levels | COL1A1\|HSPA5\|ALB | 4.86E-02 |
| cartilage development | COL1A1\|FGF2\|CTGF | 4.86E-02 |
| humoral immune response | KRT1\|CFI\|CLU | 4.86E-02 |
| regulation of anatomical structure size | MTPN\|TMSB4X\|ADAM10\|INHBA\|FGF2\|TMSB10 | 4.86E-02 |
| regulation of response to stress | HPX\|TF\|SERPINF1\|SERPINE1\|KRT1\|THBS1 | 4.86E-02 |
| positive regulation of protein modification process | HPX\|HSPA5\|HP\|FGF2\|CTGF | 4.93E-02 |
