# Supplementary material for: Mass spectrometry analysis of adipose-derived stem cells reveals a significant effect of hypoxia on pathways regulating extracellular matrix
Source: Stem Cell Res Ther. 2016 Apr 14;7:52. doi: 10.1186/s13287-016-0310-7 (PMC4831147; doi:10.1186/s13287-016-0310-7)
Supplement: Additional file 4: Table S3. — Proteins in the peptidome fraction regulated by hypoxia. (DOCX 16 kb) [file 13287_2016_310_MOESM4_ESM.docx]

**Supplemental Table 3. Proteins in the peptidome fraction regulated by hypoxia**

| Fold change^1^ | Protein |
| --- | --- |
| -23.1 | Carbonic anhydrase 1 |
| -1.7 | Collagen type 3 alpha-1 chain |
| -1.4 | Peptidyl-prolyl cis-trans isomerase A |
| 2.0 | Transforming growth factor beta-induced protein ig-h3 |
| 2.2 | Adrenomedullin |

^1^ Fold change represents protein levels in hypoxia relative to levels in normoxia
